# Patterns of antibiotic use, pathogens, and prediction of mortality in hospitalized neonates and young infants with sepsis: A global neonatal sepsis observational cohort study (NeoOBS)

**DOI:** 10.1371/journal.pmed.1004179

**Published:** 2023-06-08

**Authors:** Neal J. Russell, Wolfgang Stöhr, Nishad Plakkal, Aislinn Cook, James A. Berkley, Bethou Adhisivam, Ramesh Agarwal, Nawshad Uddin Ahmed, Manica Balasegaram, Daynia Ballot, Adrie Bekker, Eitan Naaman Berezin, Davide Bilardi, Suppawat Boonkasidecha, Cristina G. Carvalheiro, Neema Chami, Suman Chaurasia, Sara Chiurchiu, Viviane Rinaldi Favarin Colas, Simon Cousens, Tim R. Cressey, Ana Carolina Dantas de Assis, Tran Minh Dien, Yijun Ding, Nguyen Trong Dung, Han Dong, Angela Dramowski, Madhusudhan DS, Ajay Dudeja, Jinxing Feng, Youri Glupczynski, Srishti Goel, Herman Goossens, Doan Thi Huong Hao, Mahmudul Islam Khan, Tatiana Munera Huertas, Mohammad Shahidul Islam, Daniel Jarovsky, Nathalie Khavessian, Meera Khorana, Angeliki Kontou, Tomislav Kostyanev, Premsak Laoyookhon, Sorasak Lochindarat, Mattias Larsson, Maia De Luca, Surbhi Malhotra-Kumar, Nivedita Mondal, Nitu Mundhra, Philippa Musoke, Marisa M. Mussi-Pinhata, Ruchi Nanavati, Firdose Nakwa, Sushma Nangia, Jolly Nankunda, Alessandra Nardone, Borna Nyaoke, Christina W. Obiero, Maxensia Owor, Wang Ping, Kanchana Preedisripipat, Shamim Qazi, Lifeng Qi, Tanusha Ramdin, Amy Riddell, Lorenza Romani, Praewpan Roysuwan, Robin Saggers, Emmanuel Roilides, Samir K. Saha, Kosmas Sarafidis, Valerie Tusubira, Reenu Thomas, Sithembiso Velaphi, Tuba Vilken, Xiaojiao Wang, Yajuan Wang, Yonghong Yang, Liu Zunjie, Sally Ellis, Julia A. Bielicki, A. Sarah Walker, Paul T. Heath, Mike Sharland

**Affiliations:** 1 Center for Neonatal and Paediatric Infection (CNPI), Institute of Infection & Immunity, St George’s University of London, London, United Kingdom; 2 Medical Research Council Clinical Trials Unit at University College London, London, United Kingdom; 3 Department of Neonatology, Jawaharlal Institute of Postgraduate Medical Education & Research (JIPMER), Pondicherry, India; 4 Clinical Research Department, KEMRI-Wellcome Trust Research Programme, Kilifi, Kenya; 5 Centre for Tropical Medicine & Global Health, Nuffield Department of Medicine, University of Oxford, Oxford, United Kingdom; 6 The Childhood Acute Illness & Nutrition (CHAIN) Network, Nairobi, Kenya; 7 Newborn Division and WHO-CC, All India Institute of Medical Sciences, New Delhi, India; 8 Child Health Research Foundation (CHRF), Dhaka Shishu Hospital, Dhaka, Bangladesh; 9 Global Antibiotic Research and Development Partnership (GARDP), Geneva, Switzerland; 10 Department of Paediatrics and Child Health, School of Clinical Medicine, Faculty of Health Sciences, University of the Witwatersrand, Johannesburg, South Africa; 11 Department of Paediatrics and Child Health, Faculty of Medicine and Health Sciences, Stellenbosch University, South Africa; 12 Pediatric Infectious Diseases Unit, Santa Casa de São Paulo, São Paulo, Brazil; 13 Penta Foundation, Padova, Italy; 14 Queen Sirikit National Institute of Child Health, Bangkok, Thailand; 15 Department of Pediatrics, Ribeirão Preto Medical School, University of São Paulo, São Paulo, Brazil; 16 All India Institute of Medical Sciences, Department of Paediatrics, New Delhi, India; 17 Academic Hospital Paediatric Department, Bambino Gesù Children’s Hospital, Rome, Italy; 18 Faculty of Epidemiology and Population Health, Department of Infectious Disease Epidemiology, London School of Hygiene & Tropical Medicine, London, United Kingdom; 19 PHPT/IRD-MIVEGEC, Faculty of Associated Medical Sciences, Chiang Mai University, Chiang Mai, Thailand; 20 Vietnam National Children’s Hospital, Hanoi, Vietnam and Surgical Intensive Care Unit, Vietnam National Children’s Hospital, Hanoi, Vietnam; 21 Beijing Obstetrics and Gynecology Hospital, Capital Medical University, Beijing, China; 22 Neonatology Department, Seth GS Medical College and King Edward Memorial Hospital, Mumbai, India; 23 Department of Neonatology, Lady Hardinge Medical College and Kalawati Saran Children’s Hospital, New Delhi, India; 24 Department of Neonatology, Shenzhen Children’s Hospital, Shenzhen, China; 25 Laboratory of Medical Microbiology, University of Antwerp, Antwerp, Belgium; 26 Neonatal Unit, Department of Pediatrics, Queen Sirikit National Institute of Child Health, College of Medicine, Rangsit University, Bangkok, Thailand; 27 Neonatology Dept, School of Medicine, Faculty of Health Sciences, Aristotle University and Hippokration General Hospital, Thessaloniki, Greece; 28 Department of Global Public Health, Karolinska Institutet, Stockholm, Sweden; 29 Department of Paediatrics and Child Health, College of Health Sciences, Makerere University and MUJHU Care, Kampala, Uganda; 30 Makerere University - Johns Hopkins University Research Collaboration, Kampala, Uganda; 31 Amsterdam UMC, University of Amsterdam, Emma Children’s Hospital, Department of Global Health, Amsterdam, the Netherlands; 32 Chiangrai Prachanukroh Hospital, Chiang Rai, Thailand; 33 World Health Organization, Maternal, Newborn, Child and Adolescent Health Department, Geneva, Switzerland; 34 Department of Infectious Diseases, Shenzhen Children’s Hospital, Shenzhen, China; 35 Department of Paediatrics and Child Health, Charlotte Maxeke Johannesburg Academic Hospital, Johannesburg, South Africa; 36 Infectious Diseases Unit, 3rd Dept Pediatrics, School of Medicine, Faculty of Health Sciences, Aristotle University and Hippokration General Hospital, Thessaloniki, Greece; 37 School of Clinical Medicine, Faculty of Health Sciences, University of the Witwatersrand, Johannesburg, South Africa; 38 Department of Neonatology, Beijing Children’s Hospital, Capital Medical University, National Centre for Children’s Health, Beijing, China; 39 Department of Neonatology, Children’s Hospital, Capital Institute of Pediatrics, Yabao Road, Chaoyang District, Beijing, China

## Abstract

**Background:**

There is limited data on antibiotic treatment in hospitalized neonates in low- and middle-income countries (LMICs). We aimed to describe patterns of antibiotic use, pathogens, and clinical outcomes, and to develop a severity score predicting mortality in neonatal sepsis to inform future clinical trial design.

**Methods and findings:**

Hospitalized infants <60 days with clinical sepsis were enrolled during 2018 to 2020 by 19 sites in 11 countries (mainly Asia and Africa). Prospective daily observational data was collected on clinical signs, supportive care, antibiotic treatment, microbiology, and 28-day mortality. Two prediction models were developed for (1) 28-day mortality from baseline variables (baseline NeoSep Severity Score); and (2) daily risk of death on IV antibiotics from daily updated assessments (NeoSep Recovery Score). Multivariable Cox regression models included a randomly selected 85% of infants, with 15% for validation.

A total of 3,204 infants were enrolled, with median birth weight of 2,500 g (IQR 1,400 to 3,000) and postnatal age of 5 days (IQR 1 to 15). 206 different empiric antibiotic combinations were started in 3,141 infants, which were structured into 5 groups based on the World Health Organization (WHO) AWaRe classification. Approximately 25.9% (*n* = 814) of infants started WHO first line regimens (Group 1—Access) and 13.8% (*n* = 432) started WHO second-line cephalosporins (cefotaxime/ceftriaxone) (Group 2—“Low” Watch). The largest group (34.0%, *n* = 1,068) started a regimen providing partial extended-spectrum beta-lactamase (ESBL)/pseudomonal coverage (piperacillin-tazobactam, ceftazidime, or fluoroquinolone-based) (Group 3—“Medium” Watch), 18.0% (*n* = 566) started a carbapenem (Group 4—“High” Watch), and 1.8% (*n* = 57) a Reserve antibiotic (Group 5, largely colistin-based), and 728/2,880 (25.3%) of initial regimens in Groups 1 to 4 were escalated, mainly to carbapenems, usually for clinical deterioration (*n* = 480; 65.9%).

A total of 564/3,195 infants (17.7%) were blood culture pathogen positive, of whom 62.9% (*n* = 355) had a gram-negative organism, predominantly *Klebsiella pneumoniae* (*n* = 132) or *Acinetobacter* spp. (*n* = 72). Both were commonly resistant to WHO-recommended regimens and to carbapenems in 43 (32.6%) and 50 (71.4%) of cases, respectively. MRSA accounted for 33 (61.1%) of 54 *Staphylococcus aureus* isolates.

Overall, 350/3,204 infants died (11.3%; 95% CI 10.2% to 12.5%), 17.7% if blood cultures were positive for pathogens (95% CI 14.7% to 21.1%, *n* = 99/564). A baseline NeoSep Severity Score had a C-index of 0.76 (0.69 to 0.82) in the validation sample, with mortality of 1.6% (3/189; 95% CI: 0.5% to 4.6%), 11.0% (27/245; 7.7% to 15.6%), and 27.3% (12/44; 16.3% to 41.8%) in low (score 0 to 4), medium (5 to 8), and high (9 to 16) risk groups, respectively, with similar performance across subgroups. A related NeoSep Recovery Score had an area under the receiver operating curve for predicting death the next day between 0.8 and 0.9 over the first week. There was significant variation in outcomes between sites and external validation would strengthen score applicability.

**Conclusion:**

Antibiotic regimens used in neonatal sepsis commonly diverge from WHO guidelines, and trials of novel empiric regimens are urgently needed in the context of increasing antimicrobial resistance (AMR). The baseline NeoSep Severity Score identifies high mortality risk criteria for trial entry, while the NeoSep Recovery Score can help guide decisions on regimen change. NeoOBS data informed the NeoSep1 antibiotic trial (ISRCTN48721236), which aims to identify novel first- and second-line empiric antibiotic regimens for neonatal sepsis.

**Trial registration:**

ClinicalTrials.gov, (NCT03721302).

## Abstract 2

James Neal Russell and colleagues report a global neonatal sepsis observational cohort study (NeoOBS) exploring patterns of antibiotic use, pathogens and prediction of mortality in hospitalised neonates and young infants with sepsis.

## Author summary

**Why was this study done?:**

- Increasing trends in antimicrobial resistance (AMR) disproportionately affect neonates and young infants with sepsis in LMIC settings and undermine the effectiveness of WHO-recommended antibiotics.
- Despite this, longitudinal data on antibiotic management strategies and outcomes of hospitalized neonates and young infants with sepsis in low- and middle-income country (LMIC) settings are extremely limited, impeding the design of robust antibiotic trials.
- There is limited data to define risk stratification, inclusion, and escalation criteria in trials of sepsis in hospitalized neonates and young infants.

**What did the researchers do and find?:**

- In this large global, prospective, hospital-based observational study across 4 continents, including LMIC settings, there was a high mortality among infants with culture positive sepsis (almost 1 in 5), and a significant burden of antibiotic resistance.
- This study highlights wide variations in standard of care (SOC) for sepsis in neonates and young infants, with more than 200 different antibiotic combinations, significant divergence from WHO-recommended regimens, and frequent switching of antibiotics.
- A NeoSep Severity Score that defined patterns of mortality risk at baseline was developed from 4 non-modifiable and 6 modifiable factors that are feasible to measure across a range of LMIC hospital contexts.
- A NeoSep Recovery Score including the same modifiable factors (with the addition of cyanosis) predicted mortality on the following day during treatment.

**What do these findings mean?:**

- These data demonstrate that patterns of routine antibiotic use are now markedly divergent from global guidance.
- There is an urgent need for large pragmatic randomized controlled trials to address optimal empiric first- and second-line antibiotic treatment strategies in LMIC hospital settings with a significant AMR burden.
- The wide range of multiple antibiotic regimens routinely used as SOC suggests the need for novel trial designs.
- The NeoSep Severity Score and NeoSep Recovery Score informed inclusion and escalation criteria in the NeoSep1 antibiotic trial (ISRCTN48721236) that aims to identify novel first- and second-line empiric antibiotic regimens for neonatal sepsis.

## Introduction

Sepsis is responsible for a significant burden of disease in neonates and young infants, both as a primary cause of death and as a frequent contributor [1,2]. Access to facility-based delivery and care, including antibiotics, has not reduced mortality sufficiently to achieve the Sustainable Development Goal targets in many low- and middle-income countries (LMICs) [3]. Antimicrobial resistance (AMR) increasingly threatens to undermine the effectiveness of antibiotics and potentially slow progress in reducing mortality, particularly in LMICs [4–10], with AMR-attributable neonatal deaths recently estimated between 140,000 [10] and 214,000 annually [11].

Recent large-scale antibiotic trials in neonates and young infants in LMIC settings have largely focused on simplification of first-line antibiotic regimens with oral amoxicillin and short course gentamicin [12,13]. These have been based in primary healthcare settings and included populations with case fatality below 2%. An increasing global proportion of newborns are delivered in facilities [14], where sepsis case fatality rates and the burden of AMR are greater. Despite this, there is limited high-quality evidence generated from LMIC neonatal inpatient settings to guide empiric antibiotic treatment [4,15]. Published observational data largely involve single-center studies reporting non-systematically collected microbiological data, which are rarely accompanied by detailed clinical and antibiotic use data. Data regarding global antibiotic use often rely on point prevalence surveys, with limited information on patterns of switching and duration [16].

NeoOBS is a prospective multicountry observational study in which we collected detailed daily longitudinal data on clinical features, microbiology, antibiotic use, switching, and outcomes of neonatal sepsis in hospital settings, predominantly in LMICs. The objectives were to describe variation in clinical presentation and patterns of hospital-based antibiotic use and to develop 2 linked clinically based scores adapted from the World Health Organization (WHO) possible serious bacterial infection (pSBI) criteria, relevant to hospitalized neonates with sepsis in LMICs: (1) a sepsis severity score to predict 28-day mortality from factors known at sepsis presentation; and (2) a recovery score to predict the daily risk of death on treatment with IV antibiotics using daily updated assessments of clinical status. The aim of the study was to inform inclusion criteria, empiric and second-line treatment, and criteria for switching antibiotics in the design of hospital-based neonatal antibiotic trials [17].

## Methods

### Study design and participants

Hospitalized infants <60 days of age with a new episode of clinically suspected sepsis were enrolled between 2018 and 2020 in 19 hospital sites across 11 countries in Asia (Bangladesh, China, India, Thailand, and Vietnam), Africa (Kenya, South Africa, and Uganda), Europe (Italy and Greece), and South America (Brazil). Sites were selected after conducting a feasibility study [18] to represent diverse regions and to include secondary and tertiary referral hospitals, public facilities, and facilities with varying proportions of in-born and out-born infants, with access to microbiology.

Infants were eligible if the local physician had decided to treat the infant with antibiotics for a new episode of sepsis meeting the inclusion criteria (S1 Fig), derived by combining clinical and laboratory criteria from WHO pSBI [19] and EMA Criteria for neonatal sepsis trials [20]. To allow for variation in access to laboratory testing, and ensure generalizability to varying LMIC hospital contexts, laboratory values were not mandatory. A minimum of 2 clinical, or 1 clinical and 1 laboratory sepsis criteria, were required for inclusion, and up to 200 infants per site were enrolled according to a sampling frame adapted to local case volume and activity (see S1 Appendix). Infants were excluded if an alternative primary diagnosis other than sepsis was suspected, or a serious non-infective comorbidity was expected to cause death within 72 h. Previous antibiotic use was not an exclusion criterion as long as a new antibiotic regimen was being started after a blood culture for a distinct new episode of sepsis. Sepsis episodes occurring >48 h after admission, defined by time of blood culture, were defined as healthcare-associated infections (HAIs).

Ethical approval was obtained from St. George’s, University of London (SGUL) Research Ethics Committee and sites’ local, central or national ethics committees and other relevant local bodies, where required. This study is reported using (1) the Strengthening the Reporting of Observational Studies in Epidemiology (STROBE) guideline (S1 STROBE Checklist), with its design guided by the STROBE-NI framework [21]; and (2) the Transparent Reporting of a multivariable prediction model for Individual Prognosis Or Diagnosis (TRIPOD) guidelines [22]. The study was registered with ClinicalTrials.gov (NCT03721302).

### Procedures

After written consent from parents, baseline demographic and clinical data were collected, followed by prospective daily collection of observational data including multiple clinical parameters, laboratory investigations, and microbiological results. Antibiotic data were collected daily including drug, dose, route, duration, switching, and reasons given for any changes. Clinical data collection was required up to 48 h after the completion of antibiotic therapy or discharge if sooner. Aside from a mandatory blood culture at enrolment and daily monitoring of vital signs, all clinical observations and investigations were performed according to routine local site practices. Infants were followed until 28 days after enrolment in-person if still hospitalized or by telephone post-discharge. A final diagnosis was documented by clinicians, as were primary and secondary causes of death, and any clinical illness or readmission occurring after discharge and within 28 days of enrolment.

Data were collected by research and clinical staff based on clinical observation and routine source documentation (e.g., medical and nursing notes, vital signs, and prescription charts), and entered and managed using REDCap electronic data capture tools [23] hosted at SGUL (details on data monitoring in S1 Appendix).

### Microbiology/laboratory assessments

Laboratory analysis was performed in each site following local practice, with standard operating procedures developed to optimize procedures including blood culture technique, and antibiotic susceptibility testing. A locally defined algorithm was used to classify contaminants and pathogens by site clinicians and microbiologists. External validation of the capability of laboratories to detect multidrug resistant (MDR) gram-negative pathogens from each site was evaluated objectively by testing an external quality assurance (EQA) panel sent from the central laboratory at Laboratory of Medical Microbiology (LMM) at the University of Antwerp (UA).

European Committee on Antimicrobial Susceptibility Testing (EUCAST) or Clinical and Laboratory Standard Institute (CLSI) guidelines and interpretive algorithms were used to interpret reported antibiotic susceptibility testing data for all organisms. EUCAST 2019 breakpoints table (v9.0) and guidelines were used as this was when most data was reported. In most cases, where susceptibility to a particular antibiotic was reported, this was used to determine susceptibility. If the organism was considered intrinsically resistant to a particular antibiotic according to EUCAST, then it was coded as resistant regardless of reported susceptibility. If susceptibility to a particular antibiotic was not reported, susceptibility results of a different antibiotic in the same class were used to determine susceptibility (e.g., susceptibility of organism to another aminoglycoside if gentamicin susceptibility was not reported); if no other antibiotic in that class had reported susceptibility, then susceptibility was coded as unknown.

### Analysis of antibiotic patterns of use

The initial antibiotic regimen was defined as the first new antibiotic(s) started within 24 h from baseline blood culture (including 3 h pre baseline culture). To structure the analysis and reporting of the multiple regimens used, a novel method of grouping antibiotics was derived, based on the Essential Medicine List for Children (EMLc) AWaRe classification (Access, Watch, Reserve) [24], with the “Watch” category divided into 3 distinct groups of “Low/Medium/High Watch” based on inclusion in current WHO guidelines (Low Watch) and likelihood of resistance generation with regimens outside WHO recommendations (Medium or High Watch) [25]. Antibiotic groups were defined by the main “stem” in the antibiotic combination: Group 1 antibiotics included a first-line WHO-recommended penicillin-based regimen (e.g., ampicillin and gentamicin) (Access), Group 2 included third-generation cephalosporin (e.g., cefotaxime/ceftriaxone)-based WHO regimens (“Low” Watch), Group 3 included regimens with partial anti-extended-spectrum beta-lactamase (ESBL) or pseudomonal activity (e.g., piperacillin-tazobactam/ceftazidime/fluoroquinolone-based) (“Medium” Watch), and Group 4 included carbapenems (e.g., meropenem) (“High” Watch). Group 5 antibiotics included Reserve antibiotics targeting carbapenem-resistant organisms (CROs) (e.g., colistin). Aminoglycosides (e.g., gentamicin/amikacin), glycopeptides (e.g., vancomycin/teicoplanin), and metronidazole used in combination regimens were classified as additional coverage and did not define the main antibiotic “stem” for the grouping. All antifungals and antivirals were excluded from the antimicrobial treatment data as these were not relevant to the analysis. Escalation of treatment was defined as a switch to a higher group antibiotic, and de-escalation was defined as switching to a lower group or discontinuation of the “stem” antibiotic while continuing with an additional coverage antibiotic.

### Statistical analysis

Baseline characteristics are presented as raw numbers, without any weighting (S1 Appendix). The prespecified primary outcome was mortality through 28 days post-enrolment, analyzed using Kaplan–Meier and Cox proportional hazards regression with site-level random effects. Time was measured from the initial blood culture sample, censoring at the earliest of day 28, withdrawal or last contact if lost post-discharge. Aligned with the WHO pSBI criteria, we developed 2 prediction models and corresponding risk scores: (1) a baseline NeoSep Severity Score to predict 28-day mortality from factors known at sepsis presentation; and (2) a NeoSep Recovery Score to predict the daily risk of death while treated with IV antibiotics from daily updated assessments of clinical status.

#### NeoSep Severity Score

We constructed a Cox regression model for 28-day mortality based on baseline clinical parameters known at sepsis presentation (i.e., before availability of microbiological results) and used this to develop a neonatal sepsis severity score. Baseline was defined as up to 24 h from the time blood cultures were taken. Candidate predictors selected for initial consideration had missing values in <10% of the included infants and had been found to be predictive in other studies [26] or were a priori determined to be clinically important and not highly correlated with other factors (S1 Table). Factors that are not usually available in low-income settings including laboratory results (missing in >10%), and clinical signs with prevalence <5% were not considered. All analyses were based on available data, and imputation methods were not used. A 15% randomly selected sample per site was reserved for model validation and not used in any model development. This validation procedure was predefined because of expected large differences between sites in mortality, instead of randomly choosing 1 or 2 sites for validation which then might have turned out to be unrepresentative. In the remaining 85% of infants, model development used backwards elimination (exit *p* = 0.05) to identify independent predictors of mortality, considering both categorical (e.g., presence of sepsis signs) and continuous predictors (birth weight, gestational age, time in hospital, age at baseline, oxygen saturation, respiratory rate, heart rate, and temperature), taking into account the potential for nonlinear relationships (e.g., where both high and low values are associated with poor outcomes) by using fractional polynomials (FPs). FPs were implemented using the mfp command in Stata with power (−2, −1, −0.5, 0, 0.5, 1, 2) and significance level of 0.05 for testing between FP models of different degrees. Initial variable selection was done on complete cases that comprised 2,313/2,726 (85%) infants in the training set; final models were re-fitted to complete cases for the included factors (2,705/2,726 (99%) infants in the derivation sample). Interactions between birth weight and clinical predictors were examined and 1 strong interaction (*p* < 0.001) was found with ventilation support; however, this was not included in the final model in favor of simplicity for use of the prediction model in clinical practice and because including additional points for the interaction made very little difference to the C-statistic. The proportional hazards assumption was checked based on Schoenfeld residuals, and no deviations were found (*p* > 0.05). A points-based risk score, where each predictor of death is assigned a number of points, was then developed from model coefficients: For each of the categorical factors, coefficients were divided by the smallest of the coefficients (lethargy) and rounded to the nearest integer. For the continuous factors, a clinically relevant reference value was chosen (birth weight: 3,000 g; temperature: 37°C; gestational age: 39 weeks; time in hospital: 14 days), and the number of points associated with lower/higher values was then based on the difference to the reference. This initial score was then further simplified to provide a more feasible and pragmatic scale for use in LMIC by assigning 1 score point for a predictor if its value in the initial score was <3, 2 score points if its initial value was 3 to 5 points, and 3 score points if its initial value was ≥6 points. For each participant, points were added with a higher score indicating a higher risk of death. Harrell’s C-index with bootstrapped confidence intervals was used as a measure of discrimination of the prognostic Cox models, and the Hosmer–Lemeshow goodness of fit test was applied after running a logistic model with death as the independent and the score as the dependent variable as a measure of calibration.

We compared the NeoSep Severity Score with a score based on WHO pSBI [19], allocating 1 point for each of the following 6 signs: fever (≥38°C), low body temperature (<35°C), movement only on stimulation, feeding poorly, fast breathing (≥60 breaths per minute on days 0 to 6), and severe chest in-drawing. Whereas the first 5 signs were directly reported/measured in NeoOBS, the latter was only reported as part of a composite respiratory sign (severe chest wall in-drawing, increased oxygen requirement, or need for ventilation); for the score calculation, 1 point was given if this was reported.

#### NeoSep Recovery Score

To estimate the association between daily risk of death after initiating IV antibiotics and time-updated factors, and hence develop a recovery score, we used Cox proportional hazards regression with time-varying independent factors. We used the same samples as described above for model derivation and validation. Infants were censored after stop of all IV antibiotics (cause-specific model). We started model building with all clinical predictors included in the baseline severity score as time-updated factors; unmodifiable infant and birth characteristics were excluded because they cannot evolve and would have restricted interpretation of the recovery score (e.g., preterm babies would have a minimum score of 1 and could have never reached “full recovery” with a score of 0). Forward selection (entry *p* = 0.05) was then used to identify any additional independent time-updated clinical predictors. FPs were used for continuous factors as described above. For vital parameters, the last value was used per assessment period. To avoid selecting factors representing the mechanism of dying rather than being predictors of subsequent death, clinical parameters reported on the previous day were used as predictors of death on the current day in all time-updated models. A points-based risk score was then derived similarly to the baseline severity score as described above. Discrimination was assessed using time-updated area under the receiver operator curves (AUROCs), ignoring censoring as this was low (<2% overall). To examine the potential usefulness of the recovery score for informing the decision to potentially switch to second-line antibiotics in future empiric clinical trials, we focused on day 2 (48 to 72 h) post baseline, a key decisional time point during clinical management when culture results become available and response to treatment is commonly evaluated.

#### Analysis of antibiotic use

Cumulative incidence of antibiotic escalation or stop of all IV antibiotics was estimated with death as competing risk. Multivariable logistic regression with backwards elimination (exit *p* = 0.05) adjusted for site was used to analyze factors associated with starting groups 3 to 5 versus groups 1 to 2 regimens in sites with at least 10% of infants in either. Candidate factors were known at sepsis presentation and included birth weight, gestational age, postnatal age, time in hospital, central line or indwelling catheter, intravenous (IV) antimicrobials in previous 24 h, signs of meningitis, previous positive culture, previous surgery, and the NeoSep Recovery Score. FPs were used to take into account the potential for nonlinear relationships. To analyze whether time from baseline culture to the start of new antibiotic regimen or type of pathogen were associated with mortality, respectively, we adjusted for the NeoSep Severity Score. Analyses used Stata version 16.1.

## Results

A total of 3,204 infants (90.4% neonates aged <28 days, *n* = 2,895; 42.1% female, *n* = 1,348) were recruited from 20 August 2018 to 29 February 2020 (Table 1 and S2 Fig). Sites included varying populations of infants and levels of care (S3–S6 Figs and S2 Table). The median postnatal age was 5 (IQR 1 to 15) days, and 3,088 (96.4%) infants had been born in a hospital/facility (1,550 in the enrolling facility), 1,412 (44.3%) by cesarean section (969 as an emergency), and 71 (2.2%) had previously been treated for an episode of culture-positive sepsis. The median (IQR) gestational age at birth was 37 (31 to 39) weeks, with birth weight 2,500 g (1,400 to 3,000 g). At enrollment, 69.1% (*n* = 2,215) infants had been hospitalized since birth, and 30.9% (*n* = 989) were admitted from the community. A total of 2,759 (86.1%) were recruited in a neonatal unit. Among 309 (9.6%) infants enrolled aged ≥28 days, the majority (*n* = 181; 58.6%) were either ex-premature (*n* = 146; 47.4%) and/or had been admitted during the neonatal period (*n* = 136, 44.2%).

**Table 1:** Baseline characteristics.

Infant characteristics			*N* = 3,204
	WHO region of enrolment:	Africa	998 (31.1%)
		Americas	79 (2.5%)
		Southeast Asia	1,201 (37.5%)
		Europe	121 (3.8%)
		Western Pacific	805 (25.1%)
	Age at baseline (days), median (IQR)		5 (1, 15)
	Sex	Male	1,854 (57.9%)
		Female	1,348 (42.1%)
		Indeterminate/intersex	2 (0.1%)
	Birth weight (grams), median (IQR)		2,500 (1,400, 3,200)
	Gestational age at birth (weeks), median (IQR)		37 (31, 39)
	Estimated gestational age at birth, categories		
		Extremely preterm (<28 weeks)	227 (7.1%)
		Very preterm (28 to <32 weeks)	607 (19.0%)
		Moderately preterm (32 to <37 weeks)	694 (21.7%)
		Term (≥37 weeks)	1,674 (52.3%)
Birth history	Birth status	Hospitalized since birth	2,215 (69.1%)
		Admitted from home/community	989 (30.9%)
	Time from admission to enrolment (hours), median (IQR)		22 (1, 126)
	Mode of delivery:	Vaginal	1,774 (55.4%)
		Emergency cesarean section	969 (30.2%)
		Elective cesarean section	443 (13.8%)
	Congenital anomalies		265 (8.3%)
Common subgroups	Early-onset (age <48 h)		1,066 (39.3%)
	Late onset community presenting, term		708 (26.1%)
	Late onset healthcare associated, preterm		936 (34.5%)
	Other		494 (15.4%)
Antibiotics	On IV antibiotics in previous 24 h		742 (23.2%)
Previous surgery	Abdominal surgery or for congenital malformations		95 (3.0%)
Supportive care at baseline	IV fluid (supportive/feeding)		2,497 (77.9%)
	Thermal care	Incubator care	984 (30.7%)
		Heated mattress	115 (3.6%)
		Overhead heater	(33.7%)
		Kangaroo mother care	163 (5.1%)
	Oxygen supplementation		1,894 (59.1%)
	Ventilation support:	Non-invasive ventilation	719 (22.4%)
		Invasive ventilation	701 (21.9%)
	Phototherapy		522 (16.3%)
	Transfusion red blood cells		221 (6.9%)
	Transfusion platelets		64 (2.0%)
	Fresh frozen plasma		118 (3.7%)
	Feeding:	Breast/formula/other milk	1,953 (61.0%)
		TPN	527 (16.4%)
		Nasogastric tube	1,432 (44.7%)
Baseline culture	Negative		2,502 (78.3%)
	Contaminant (presumed non-pathogen)		117 (3.7%)
	Pathogen		564 (17.7%)
	Indeterminate		12 (0.4%)
Pathogens	*Klebsiella pneumoniae*		132 (4.1%)
	Coagulase-negative Staphylococci		84 (2.6%)
	*Acinetobacter* spp. *		72 (2.3%)
	*Staphylococcus aureus*		54 (1.7%)
	*Escherichia coli*		47 (1.5%)
	*Enterobacter* spp.		39 (1.2%)
	*Serratia* spp.		20 (0.6%)
	*Streptococcus agalactiae*		19 (0.6%)
	Other gram-negative bacteria **		65 (2.0%)
	Other gram-positive bacteria ***		42 (1.3%)
	Fungi ****		21 (0.7%)
Vital parameters	Oxygen saturation (%), median (IQR)		96 (93, 98)
	Respiratory rate, median (IQR)		52 (44, 60)
	Heart rate, median (IQR)		148 (138, 161)
	Temperature (°C)	<35.5	58 (1.8%)
		35.5–37.9	2,736 (85.6%)
		≥38–<39	320 (10.0%)
		≥39	84 (2.6%)
Clinical signs at baseline (≥5%)	Respiratory signs ^‡^		2,107 (65.8%)
	Difficulty feeding ͳ		1,465 (45.7%)
	Lethargy/reduced movement	Neither	2,073 (64.7%)
		Lethargy only	803 (25.1%)
		Reduced/no movement	328 (10.2%)
	Abdominal distension		777 (24.3%)
	Evidence of shock		683 (21.3%)
	Apnoea		550 (17.2%)
	Jaundice requiring phototherapy		494 (15.4%)
	Grunting		435 (13.6%)
	Hypotonia/floppiness		435 (13.6%)
	Cyanosis		384 (12.0%)
	Irritability		322 (10.0%)
	Vomiting		287 (9.0%)
	Convulsions		236 (7.4%)

*Acinetobacter baumannii: *n* = 64; *Acinetobacter iwoffii*: *n* = 2; unspecified: *n* = 6.

**Enterococcus faecalis: *n* = 14; *Enterococcus faecium*: *n* = 10; *Bacillus* spp.: *n* = 4; *Streptococcus pneumoniae*: *n* = 3; *Streptococcus pyogenes*: *n* = 3; *Aerococcus viridans*: *n* = 1; *Corynebacterium* spp.: *n* = 1; *Dermabacter hominis*: *n* = 1; *Listeria monocytogenes*: *n* = 1; *Streptococcus gallolyticus*: *n* = 1; *Streptococcus salivarius*: *n* = 1; *Streptococcus vestibularis*: *n* = 1; *Streptococcus viridans*: *n* = 1.

***Elizabethkingia meningoseptica: *n* = 15; *Burkholderia* spp.: *n* = 12; *Citrobacter* spp.: *n* = 10; *Elizabethkingia anophelis*: *n* = 7; *Klebsiella oxytoca*: *n* = 7; *Pseudomonas aeruginosa*: *n* = 5; *Proteus mirabilis*: *n* = 2; *Campylobacter coli*: *n* = 1; *Klebsiella* spp.: *n* = 1; *Morganella morgannii*: *n* = 1; *Pseudomonas putida*: *n* = 1; *Pseudomonas stutzeri*: *n* = 1; *Salmonella* spp.: *n* = 1; *Sphingomonas paucimobilis*: *n* = 1; *Stenotrophomonas maltophilia*: *n* = 1.

****Candida albicans: *n* = 13; *Candida parapsilosis*: *n* = 2; *Candida pelliculosa*: *n* = 2; *Candida auris*: *n* = 1; *Candida tropicalis*: *n* = 1; *Kodamaea ohmeri*: *n* = 1; *Wickerhamomyces anomalus*: *n* = 1.

^**‡**^Severe chest wall in-drawing, increased requirement for oxygen, or respiratory support.

^ͳ^Observed or reported, including feeding intolerance.

IV, intravenous.

The most common previously identified risk factors for sepsis other than prematurity were preterm premature rupture of membranes (14.5%, *n* = 466), prolonged rupture of membranes (>18 h) (10.2%, *n* = 328), pre-labor rupture of membranes at term (9.4%, *n* = 300), presence of an indwelling central vascular catheter (8.2%, *n* = 262), intrapartum fever >38°C (3.5%, *n* = 112), chorioamnionitis (2.8%, *n* = 75), known maternal Group B streptococcus (GBS) colonization (1.4%, *n* = 46) and previous surgery (abdominal or for congenital malformations: 3.0%, *n* = 95). Of note, 8.3% (*n* = 265) had a congenital anomaly (S3 Table), and 7% (*n* = 220) of infants were exposed to maternal HIV. A total of 1,318 (41.1%) sepsis episodes were classified as healthcare-associated (occurring >48 h after hospital admission).

### Clinical and laboratory findings

A median of 4 (IQR 2 to 5) clinical signs were present at baseline, the most common being respiratory (65.8%, *n* = 2,107), difficulty feeding (45.7%, *n* = 1,464), lethargy or reduced movement (35.3%, *n* = 1,131), abdominal distension (24.3%, *n* = 777), and evidence of shock (21.3%, *n* = 683), with prevalence decreasing over time (Table 1 and S7 Fig). Signs suggestive of meningitis were reported in <10% of infants (irritability, convulsions, abnormal posturing, bulging fontanelle) (S8 Fig). The availability of laboratory values varied by site. At baseline, a base excess <-10 mmol/L was documented in 352 of 1,492 with results available (23.6%), lactate >2 mmol in 1,034/1,283 (80.6%), raised CRP >10 mg/L in 1,306/2,286 (57.1%), abnormal white blood cell count (<4 or >20 × 10^9^ cells/L) in 875/2,800 (31.3%), and thrombocytopenia (<150 × 10^9^ cells/L) in 619/2,776 (22.3%) (S4 Table).

### Patterns of empiric antibiotic use

A total of 1,180 (36.8%) infants had a history of previous intravenous antibiotic exposure, and 742 (23.2%) had been receiving an intravenous antibiotic in the previous 24 h before starting new antibiotics for the new episode of sepsis (85.3% treatment (*n* = 633) and 14.2% prophylaxis (*n* = 105)). Median time from baseline culture being taken to new intravenous antibiotic treatment being started for the distinct sepsis episode was 1 h (IQR 0 to 3); 2,913 (90.9%) started within 8 h, and 228 (7.1%) within 8 to 24 h (S9 Fig); 63 (2.0%) infants did not start any new antibiotic in the first 24 h.

**Fig 1:**
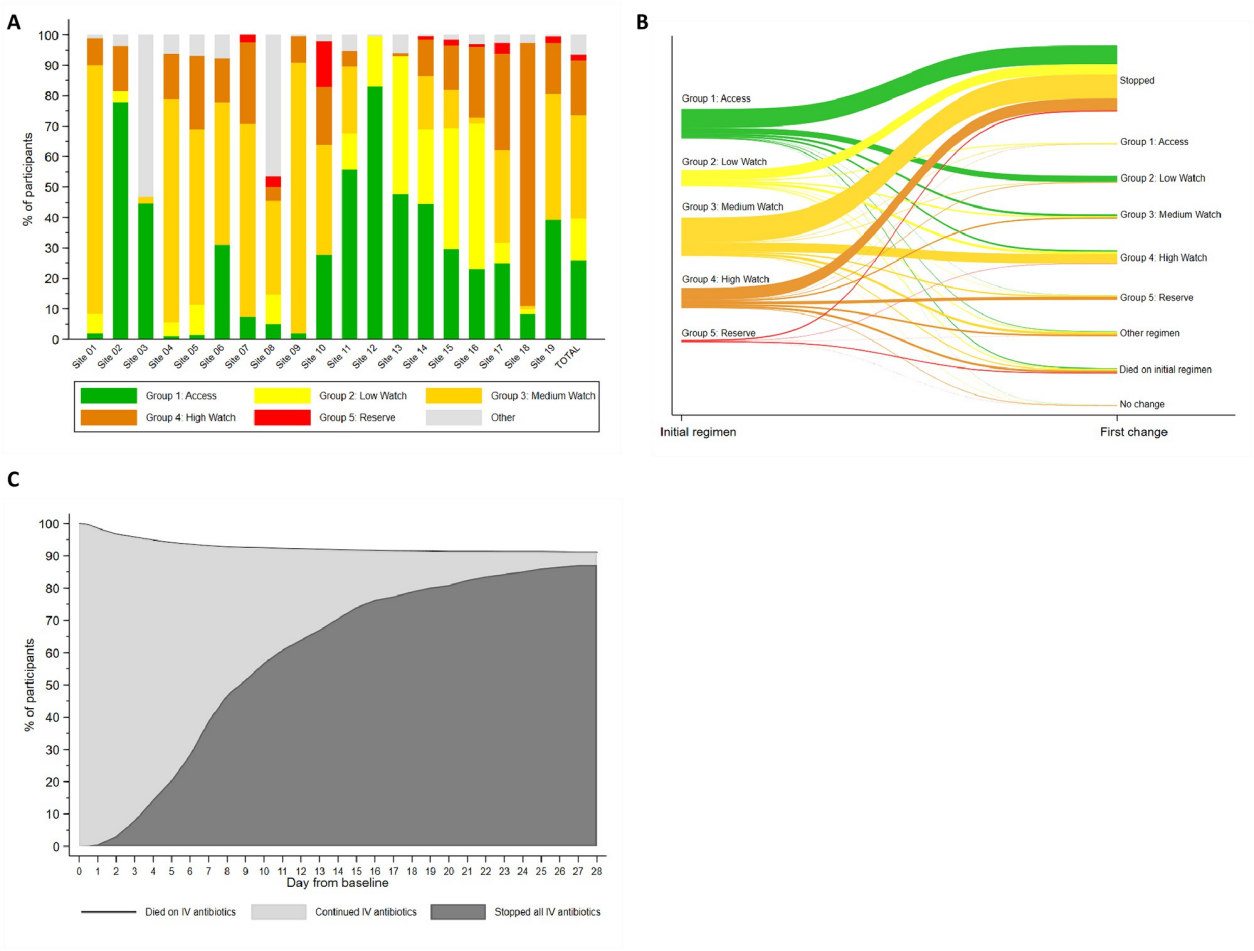
Antibiotic treatment. (A) Initial empiric baseline therapy overall and by site. (B) First change of initial regimen, by baseline regimen. (C) Cumulative incidence of stopping all IV antibiotics (death on IV antibiotics as competing risk). Group 1 = First-line WHO-recommended penicillin-based regimen (e.g., ampicillin and gentamicin) (Access). Group 2 = third-generation cephalosporin (e.g., cefotaxime/ceftriaxone)-based WHO regimens (“Low” Watch). Group 3 = regimens with partial anti-ESBL or pseudomonal activity (e.g., piperacillin-tazobactam/ceftazidime/fluoroquinolone-based) (“Medium” Watch). Group 4 = Carbapenems (“High” Watch). Group 5 = Reserve antibiotics targeting carbapenem-resistant organisms (e.g., colistin). ESBL, extended-spectrum beta-lactamase; IV, intravenous.

There were 206 different combinations of empiric antibiotics started at baseline in this hospital-based cohort. The most frequent regimens are reported in S5 Table, and these were grouped as described in the methods. Of 3,141 infants who started new antibiotics within 24 h, 25.9% (*n* = 814) were started on a WHO Neonatal Sepsis Guideline recommended first-line penicillin-based regimen (Group 1—Access), and 13.8% (*n* = 432) were started on a WHO second-line cefotaxime or ceftriaxone-based combination (Group 2—“Low” Watch) (Fig 1A). The largest group (34.0%, *n* = 1,068) were started on a regimen providing partial ESBL/pseudomonal coverage (piperacillin-tazobactam, ceftazidime, or fluoroquinolone-based) (Group 3—“Medium” Watch). Of Group 3 regimens, ceftazidime ± amikacin (*n* = 436; 13.9%) and piperacillin/tazobactam ± amikacin (*n* = 410; 13.1%) were most common. Approximately 18.0% (*n* = 566) of initial regimens were carbapenem-based (Group 4—“High” Watch), among whom meropenem ± vancomycin (*n* = 447; 14.2%) was the most common combination; 1.8% (*n* = 57) of initial regimens were classified as Group 5 antibiotics targeting CROs, predominantly colistin-based (Group 5—Reserve) (S10–S14 Figs). An “Other” group (*n* = 204) consisted of more rarely used local regimens not on the WHO EMLc, or regimens which did not include a new antibiotic “stem” that was used to define Groups 1 to 5 (e.g., aminoglycoside or glycopeptide given alone or in combination with each other) (S15 Fig). Cefoperazone/sulbactam (*n* = 99) was the most common antibiotic in this category, but was used as initial regimen only in India (*n* = 86; 14.5%), China (*n* = 11; 1.9%), and Vietnam (*n* = 2; 1.0%).

There was wide variation between sites in the frequency of empiric use of different antibiotic groups (Fig 1A). Some sites used predominantly Group 1 antibiotics as the initial regimen, and others often started immediately with Group 3 or 4 regimens, or a mixture of all groups. The most frequently prescribed empiric regimens used for HAIs were meropenem ± vancomycin (28.2%, 361/1,280), followed by piperacillin/tazobactam ± amikacin (17.5%, *n* = 224), ceftazidime ± amikacin (6.7%, *n* = 86), colistin (4.3%, *n* = 55), and cefoperazone/sulbactam (3.9%, *n* = 50). Ampicillin + gentamicin was the most common regimen for non-HAI (19.7%, 366/1,861), followed by ceftazidime ± amikacin (18.8%, *n* = 350) and piperacillin/tazobactam ± amikacin (10.0%, *n* = 186) (S6 Table).

Adjusting for site, predictors of starting empiric therapy with Groups 3 to 5 rather than Groups 1 to 2 antibiotics included lower birth weight (OR = 0.57 per additional kg, 95% CI 0.47 to 0.69), presence of a central vascular catheter (OR = 3.48, 95% CI 1.74 to 6.94), previous antibiotics at enrollment (OR = 5.71, 95% CI 3.73 to 8.77), previous culture positive sepsis (OR = 25.71, 95% CI 3.00 to 220.7), longer time in hospital (48 versus 0 hours: OR = 4.41, 95% CI 3.41 to 5.69), previous surgery (OR = 5.18, 95% CI 1.65 to 16.28), and higher sepsis severity (OR = 1.27 per additional score point, 95% CI 1.16 to 1.40) (see S7 Table).

### Antibiotic switching

After initial therapy, 728/2,880 (25.3%) who started on Group 1 to 4 regimens were escalated to a higher group regimen, the majority switching within the first days of treatment (S16–S18 Figs), and 258/814 (31.7%) infants escalated from Group 1 (ampicillin/gentamicin) regimens, the majority of whom switched to Group 2 (cefotaxime/ceftriaxone-based) regimens (61.6%, *n* = 159) (Fig 1B). A total of 101/432 (23.4%) infants escalated from Group 2 regimens, mostly directly to a Group 4 carbapenem-based regimen (62.4%, *n* = 63) rather than a Group 3 partial ESBL/pseudomonal activity regimen (32.7%, *n* = 33); 287/1,068 (26.9%) escalated from Group 3 regimens, and of 566 infants starting treatment with carbapenems (Group 4), 82 (14.5%) escalated therapy to a colistin-containing regimen. Common reported reasons for first escalation of antibiotics overall included clinical deterioration (65.9%, *n* = 480), microbiology results (15.4%, *n* = 112: *n* = 48 pathogen identification, *n* = 37 susceptibility, *n* = 27 gram stain), and worsening inflammatory biomarkers (9.8%, *n* = 71). De-escalation of antibiotics was rare (173/2,937; 5.9%).

Cumulative incidence of stopping intravenous treatment within 7 days from baseline culture was 38.9% (95% CI 37.2% to 40.6%) overall (Fig 1C); 45.8% (95% CI 43.8% to 47.6%) in pathogen-negative and 6.9% (95% CI 5.0% to 9.2%) in pathogen-positive cases. After stopping intravenous antibiotics, 350/2,803 (12.5%) switched to oral therapy, and 289 of 2,803 infants who had stopped (10.3%), restarted intravenous antibiotics during the original hospital stay, and a further 84 infants after discharge. A total of 115 (3.4%) were still on uninterrupted antibiotic treatment at day 28. Median total number of days on intravenous antibiotics during the 28 days follow-up was 8 (IQR 6 to 14) days (S19 Fig). Intramuscular use of antibiotics was only reported for 2 infants (<0.1%).

### Microbiology

**Fig 2:**
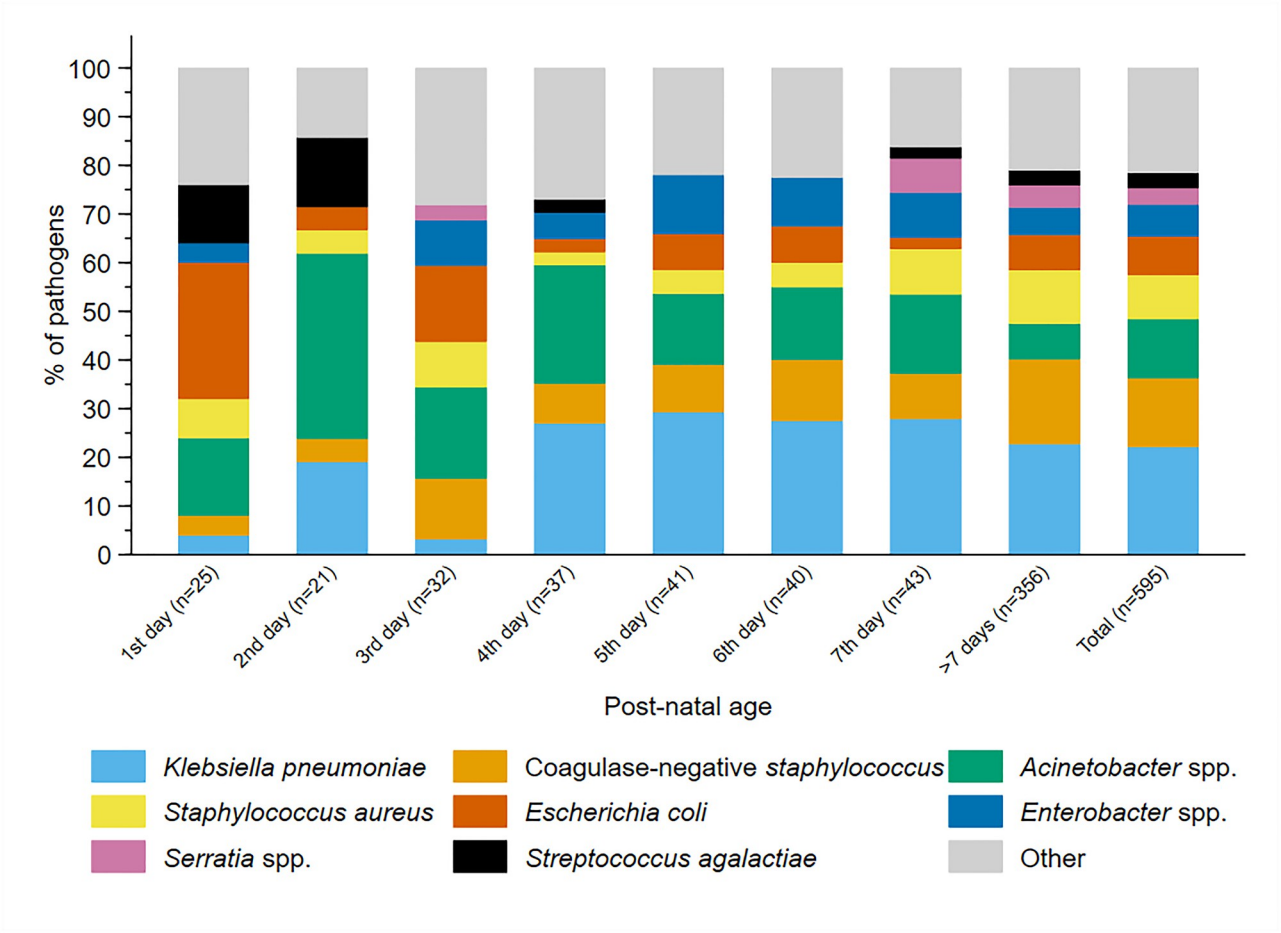
Pathogens isolated in baseline blood culture, overall and by infants’ day of life.

Initial blood culture results were available for 3,195 (99.7%) infants; 693 (21.7%) grew at least 1 organism. Organisms identified as significant pathogens were isolated in 564/693 (>1 pathogen in 29) blood cultures, contaminants (presumed non-pathogens) in 117, and indeterminate in 12 cultures (Table 1). Gram-negative and gram-positive pathogens were found in 62.9% (355/564) and 34.8% (196/564) of infants, respectively (*n* = 8 with both), and fungal pathogens in 21. Among infants with a significant pathogen, *Klebsiella pneumoniae* (23.4%, *n* = 132), Coagulase-negative staphylococci (CoNS) (14.9%, *n* = 84), *Acinetobacter* species (12.8%, *n* = 72), *S*. *aureus* (9.6%, *n* = 54), and *Escherichia coli* (8.3%, *n* = 47) were the most common (Table 1 and S20 Fig) but with differences between sites (S21 Fig). *Streptococcus agalactiae* was found in only 19 babies (3.4%). All the common pathogens were identified in both early and late onset sepsis (Fig 2), although *E*. *coli* was more common in the first 3 days of life while *K*. *pneumoniae* was more frequent in late onset sepsis (S8 Table).

**Fig 3:**
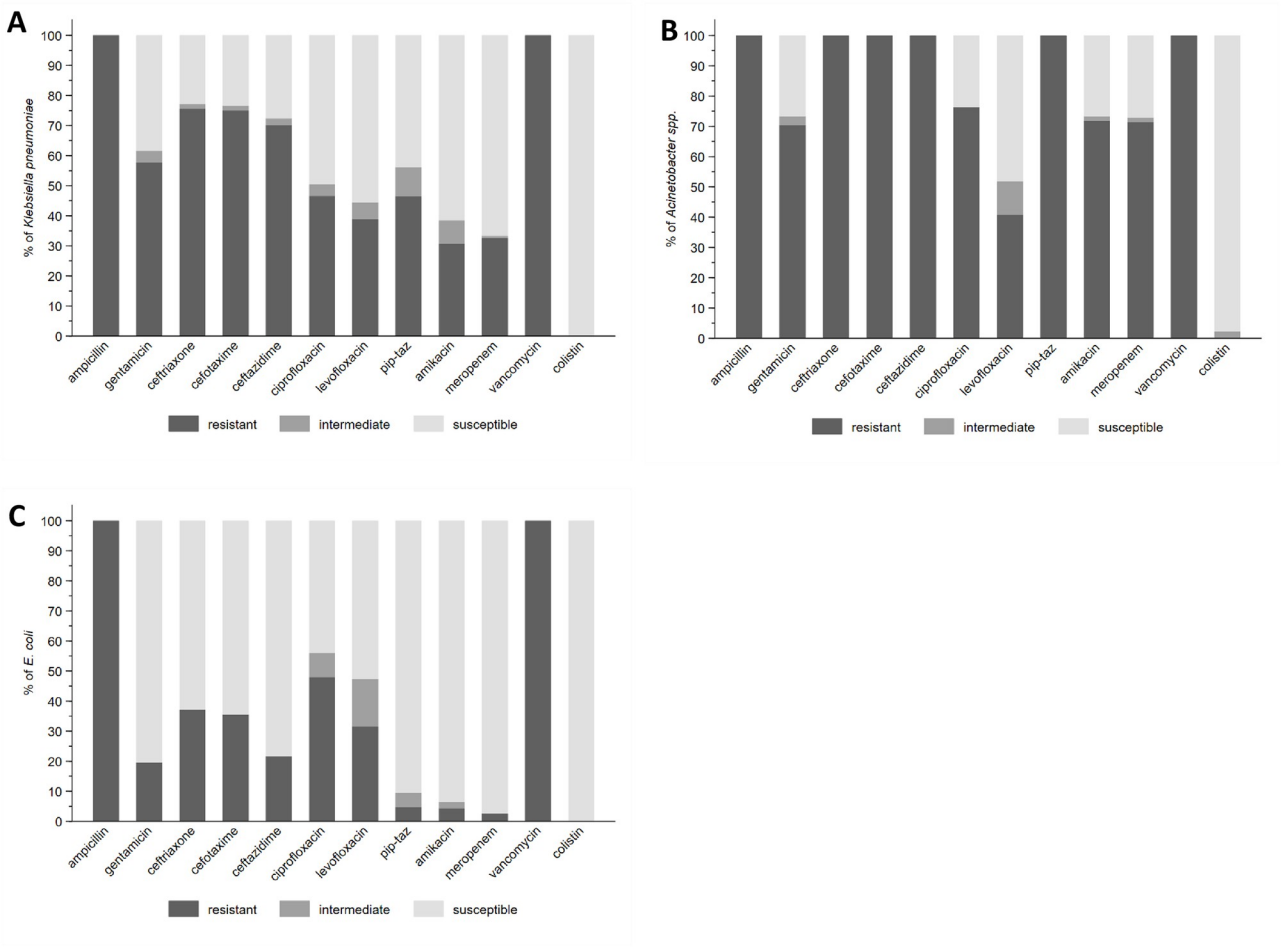
Antibiotic resistance among leading pathogens in baseline blood culture. (A) *K*. *pneumoniae* (*n* = 132). (B) *Acinetobacter* spp. (*n* = 72). (C) *E*. *coli* (*n* = 47). pip-taz = piperacillin/tazobactam.

Approximately 58% (75/130 tested) of *K*. *pneumoniae* isolates were resistant to gentamicin, 75.0% (96/128) to commonly used third-generation cephalosporins (cefotaxime/ceftriaxone), 46.5% (53/114) to piperacillin-tazobactam, 46.6% (48/103) to ciprofloxacin, and 32.6% (43/132) to meropenem (S9 Table). *Acinetobacter* species were resistant to meropenem in 71.4% (50/70) of cases. *E*. *coli* retained greater susceptibility to third-generation cephalosporins (64.4%, 29/45 susceptible) and was susceptible in 90.4% (38/42) of cases to piperacillin-tazobactam. Among gram-negatives, there were important differences in susceptibility among aminoglycosides, with amikacin providing significantly better activity than gentamicin (e.g., 61.5% versus 38.5% susceptibility among *K*. *pneumoniae*) (Fig 3 and S9 Table).

Among 54 *S*. *aureus* isolates, 33 (61.1%) were methicillin resistant. *S*. *aureus* and CoNS were susceptible to vancomycin in all of 42 and 73 isolates tested, respectively, and all 19 *Streptococcus agalactiae* isolates were susceptible to ampicillin. Other rarer and more site-specific pathogens with high rates of resistance to antibiotics included *Serratia species*, *Burkholderia species*, and *Elizabethkingia meningoseptica* (S9 Table).

A total of 1,226/3,204 (38.3%) infants had a cerebrospinal fluid (CSF) culture performed at baseline or during the subsequent 7 days, and 73/1,226 (6.0%) were culture-positive, 47 with a pathogen, and 26 contaminant/indeterminate. Gram-negative organisms also dominated in CSF cultures (S10 Table).

### Mortality

**Fig 4:**
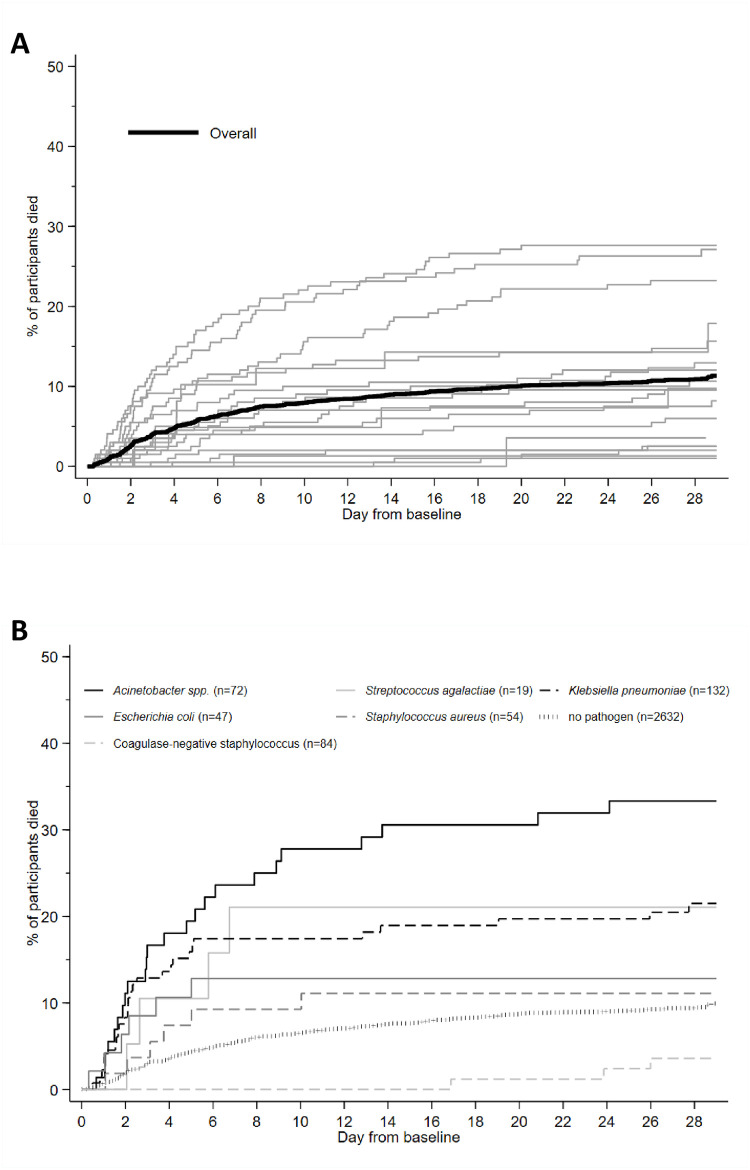
Mortality. (A) Overall mortality and mortality by site. (B) Mortality by pathogen in baseline blood culture. Figures present unadjusted Kaplan–Meier estimates.

Median follow-up time was 29 (IQR 28 to 29) days. Overall, 350 infants (11.3%; 95% CI 10.2% to 12.5%) died within 28 days of baseline blood culture. Mortality at 28 days was unknown in 62 infants (5 withdrew in hospital, 57 were lost post-discharge). There was significant variation in mortality between different sites (Fig 4A). Mortality among infants with a pathogen-positive baseline culture was 17.7% (99/564; 95% CI 14.7% to 21.1%) compared with 9.9% (250/2,631; 95% CI 8.8% to 11.2%) in infants without pathogens (*p* < 0.001). Mortality was higher in infants with gram-negative (21.3%; 95% CI 17.4% to 25.9%) or fungal pathogens (38.1%; 95% CI 21.2% to 61.9%) than with gram-positive pathogens (8.5%; 95% CI 5.3% to 13.5%; *p* < 0.001). Mortality was 33.3% (95% CI 23.7% to 45.5%) in infants with *Acinetobacter* spp., 21.5% (95% CI 15.4% to 29.6%) with *K*. *pneumoniae*, 21.1% (95% CI 8.5% to 46.8%) with *Streptococcus agalactiae*, 12.8% (95% CI 6.0% to 26.3%) with *E*. *coli*, 11.1% (95% CI 5.2% to 23.1%) with *S*. *aureus*, and 3.6% (95% CI 1.2% to 10.8%) with CoNS (Fig 4B and S11 Table). Overall, gram-negative infections accounted for 75/99 (75.8%) of culture-positive deaths, especially *K*. *pneumoniae* (*n* = 28; 28.3%) and *Acinetobacter* spp. (*n* = 24; 24.2%).

There was no significant association between time from blood culture to start of new antibiotics and mortality, neither overall (HR = 1.01 per additional 2 h [95% CI 0.96 to 1.07]; *p* = 0.60) nor in the subgroup with no previous antibiotic exposure (HR = 1.03 [95% CI 0.95 to 1.11]; *p* = 0.53) (S22 Fig).

### Baseline predictors of 28-day mortality

**Fig 5:**
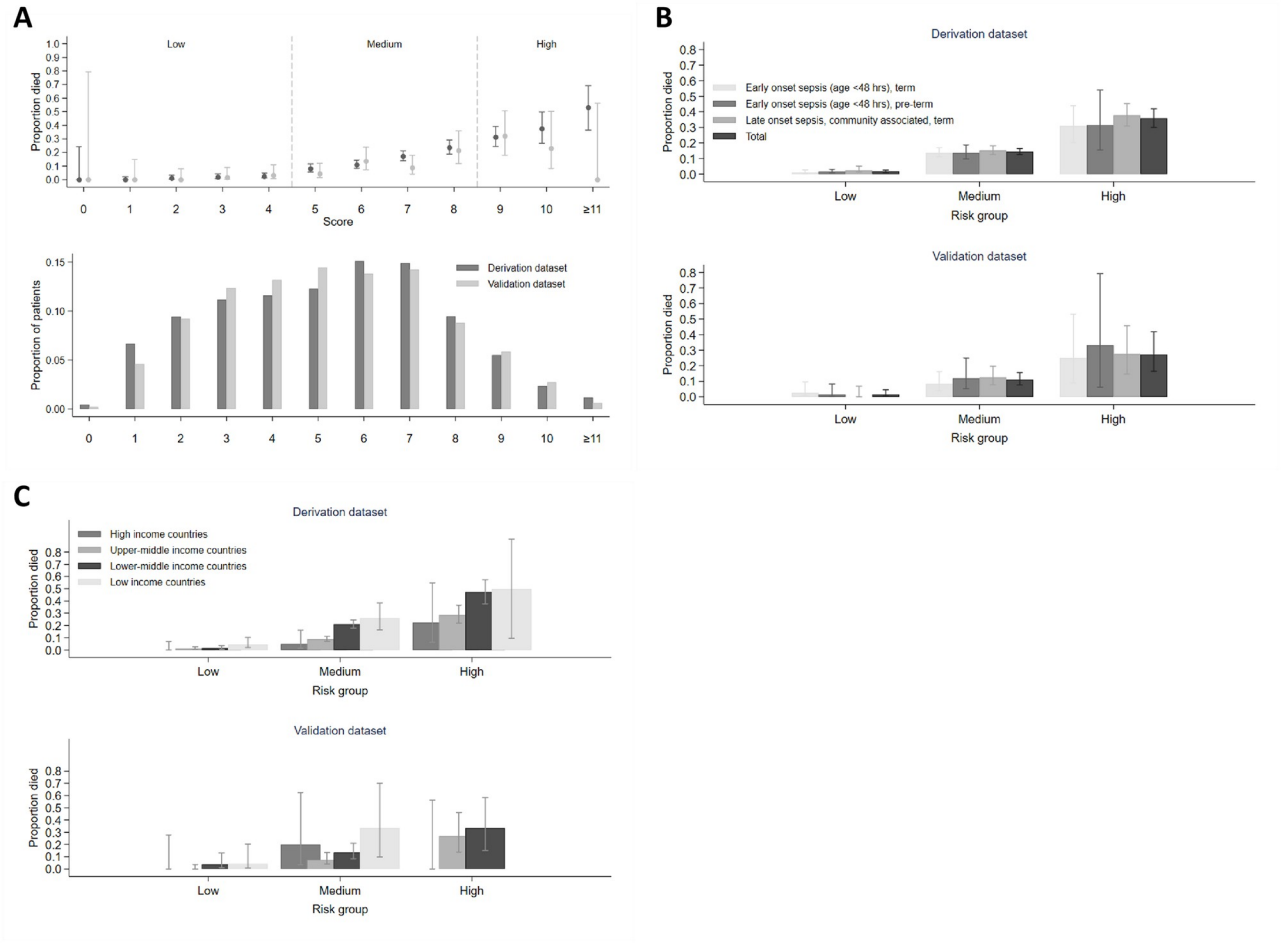
NeoSep Severity Score at baseline. (A) Distribution of the Severity Score at baseline (bottom), and the proportion (95% CI) of infants who eventually died within 28 days per score point (top) in the derivation (dark gray) and in the validation dataset (light gray). (B) Mortality (95% CI) in risk groups based on the Severity Score and selected sepsis subgroups. (C) Mortality (95% CI) in risk groups based on the Severity Score and region.

A prediction model was derived from 2,726 participants (*n* = 308 died) and validated in 478 (*n* = 42 died) (S12 and S13 Tables). Ten clinical factors known at presentation independently predicted mortality in the final model, including infant characteristics (birth weight, gestational age, duration of time in hospital, and congenital anomalies), level of respiratory support, and clinical signs (abnormal temperature, abdominal distension, lethargy/no or reduced movement, difficulty feeding, and evidence of shock) (Table 2). Risk was increased with both low (<35°C) and high temperature (≥38°C) with evidence of even higher risk if ≥39°C. The C-statistic for this model was 0.79 (95% CI: 0.77 to 0.81) and 0.81 (0.73 to 0.85) in the derivation and validation sample, respectively. A NeoSep Severity Score developed from these baseline predictors had a maximum 16 points (Table 2), with C-statistics 0.77 (95% CI: 0.75 to 0.80) and 0.76 (0.69 to 0.82) in the derivation and validation samples, respectively, and a good fit in the validation sample (Hosmer–Lemeshow *p* = 0.53). Defining mortality risk thresholds at 5% and 25% in the derivation sample categorized scores as low (score 0 to 4), medium (5 to 8), and high (9 to 16) risk, with mortality in the derivation sample of 1.6% (17/1,071; 95% CI: 1.0% to 2.5%), 14.4% (203/1,409; 12.7% to 16.3%), and 35.8% (88/246; 30.0% to 41.9%), and in the validation sample of 1.6% (3/189; 95% CI: 0.5% to 4.6%), 11.0% (27/245; 7.7% to 15.6%), and 27.3% (12/44; 16.3% to 41.8%) (Fig 5A).

**Table 2:** Predictors of mortality and risk scores.

		NeoSep Severity Score (at presentation)				NeoSep Recovery Score (daily on IV antibiotics)			
Factor		Coefficient (SE)	*p*-value	HR (95% CI)	Severity score points¶	Coefficient (SE)	*p*-value	HR (95% CI)	Recovery score points¶
Birth weight (kg) ‡	birth_wt^-2-0.181326	0.754 (0.162)	<0.001	1 kg: 2.13 (1.55, 2.92) 2 kg: 1.21 (1.12, 1.31) 3 kg: ref	**<1 kg: 2** **1–2 kg: 1**				
Time in hospital	Per additional 24 h	−0.039 (0.011)	<0.001	0.96 (0.94, 0.98)	**≤ 10 days: 1**				
Gestational age	Per additional week	−0.064 (0.022)	0.004	0.94 (0.90, 0.98)	**< 37 weeks: 1**				
Congenital anomalies	Presence	1.032 (0.191)	<0.001	2.81 (1.93, 4.08)	**2**				
Maximum respiratory support:	Oxygen supplementation CPAP, BiPAP, HFNC Invasive ventilation	1.055 (0.256) 1.551 (0.270) 2.318 (0.265)	<0.001 <0.001 <0.001	2.87 (1.74, 4.74) 4.72 (2.78, 8.00) 10.2 (6.05, 17.1)	**2** **3** **3**	2.221 (0.421) 2.721 (0.422) 3.883 (0.415)	<0.001 <0.001 <0.001	9.21 (4.04, 21.0) 15.2 (6.65, 34.8) 48.6 (21.5, 110)	**2** **3** **3**
Temperature (°C) ‡	Term 1: temperature^2–1371.2072 term 2: temperature^2*ln(temperature)-4952.4209	−0.740 (0.240) 0.181 (0.058)	0.002 0.002	35.5°C: 1.17 (0.78, 1.75) 37°C: ref 38°C: 1.41 (1.18, 1.69) 39°C: 2.91 (1.76, 4.81)	**<35.5°C: 1** **≥38 –<39°C: 1** **≥39°C: 2**	-0.839 (0.228) 0.205 (0.056)	<0.001 <0.001	35.5°C: 1.32 (0.97, 1.79) 37°C: ref 38°C: 1.43 (1.07, 1.92) 39°C: 3.07 (1.50, 6.31)	**<35.5°C: 1** **≥38 –<39°C: 1** **≥39°C: 2**
Abdominal distension	Presence	0.468 (0.131)	<0.001	1.60 (1.24, 2.07)	**1**	0.407 (0.146)	0.005	1.50 (1.13, 2.00)	**1**
Lethargy, no or reduced movement:	Lethargy only No/reduced movement (± lethargy)	0.246 (0.142) 0.695 (0.198)	0.083 <0.001	1.28 (0.97, 1.69) 2.00 (1.36, 2.95)	**1** **2**	0.617 (0.170) 1.130 (0.203)	<0.001 <0.001	1.85 (1.33, 2.59) 3.10 (2.08, 4.61)	**1** **2**
Difficulty feeding	Presence	0.406 (0.141)	0.004	1.50 (1.14, 1.98)	**1**	0.763 (0.163)	<0.001	2.15 (1.56, 2.95)	**1**
Evidence of shock	Presence	0.527 (0.143)	<0.001	1.69 (1.28, 2.24)	**1**	0.921 (0.167)	<0.001	2.51 (1.81, 3.48)	**1**
Cyanosis	Presence					0.624 (0.203)	0.002	1.87 (1.25, 2.78)	**1**
Maximum number of score points possible					**16**				**11**

This table shows results derived from 2 different multivariable Cox proportional hazards models with site-level random effects: (1) for a baseline NeoSep Severity Score to predict 28-day mortality from factors known at sepsis presentation; and (2) for a NeoSep Recovery Score to predict the daily risk of death while treated with IV antibiotics from daily updated assessments of clinical status.

^‡^ Birth weight and temperature were analyzed as continuous variables using FPs with powers −2 for birth weight, and powers 2 2 for temperature. For illustrative reasons, in this table we report HRs for specific values.

^¶^ For each model, score points for each factor were derived by dividing its coefficient by the smallest significant coefficient of any categorical factor, rounding to the nearest integer, and giving 1 score point for <3 raw points, 2 score points for 3–5 raw points, and 3 score points for ≥6 raw points. Shaded factors: not a significant predictor (cyanosis: baseline severity score) or deliberately not included in model (unmodifiable factors excluded from recovery score: birth weight, time in hospital, gestational age, congenital anomalies).

BiPAP, Bilevel Positive Airway Pressure; CI, confidence interval; CPAP, Continuous Positive Airway Pressure; FP, fractional polynomial; HFNC, High Flow Nasal Cannula; HR, hazard ratio; SE, standard error.

The association between the NeoSep Severity Score and mortality was similar within multiple subgroups, for example, in early or late-onset, community or healthcare-associated, high-, middle- or low-income settings (Fig 5B and 5C), term versus preterm infants, blood culture-positive versus culture-negative cases (S23 Fig).

A score based on WHO pSBI criteria had a C-statistic of 0.63 (95% CI 0.56 to 0.70) in the validation sample, lower than the NeoSep Severity Score. Because the WHO pSBI criteria do not include any infant/birth characteristics, to ensure an appropriate comparison, we also calculated a modified NeoSep Severity Score excluding these factors that had a C-statistic of 0.72 (95% CI 0.65 to 0.77) (S24 Fig).

### Predictors of daily risk of dying of sepsis while on intravenous antibiotics in hospital: NeoSep Recovery Score

**Fig 6:**
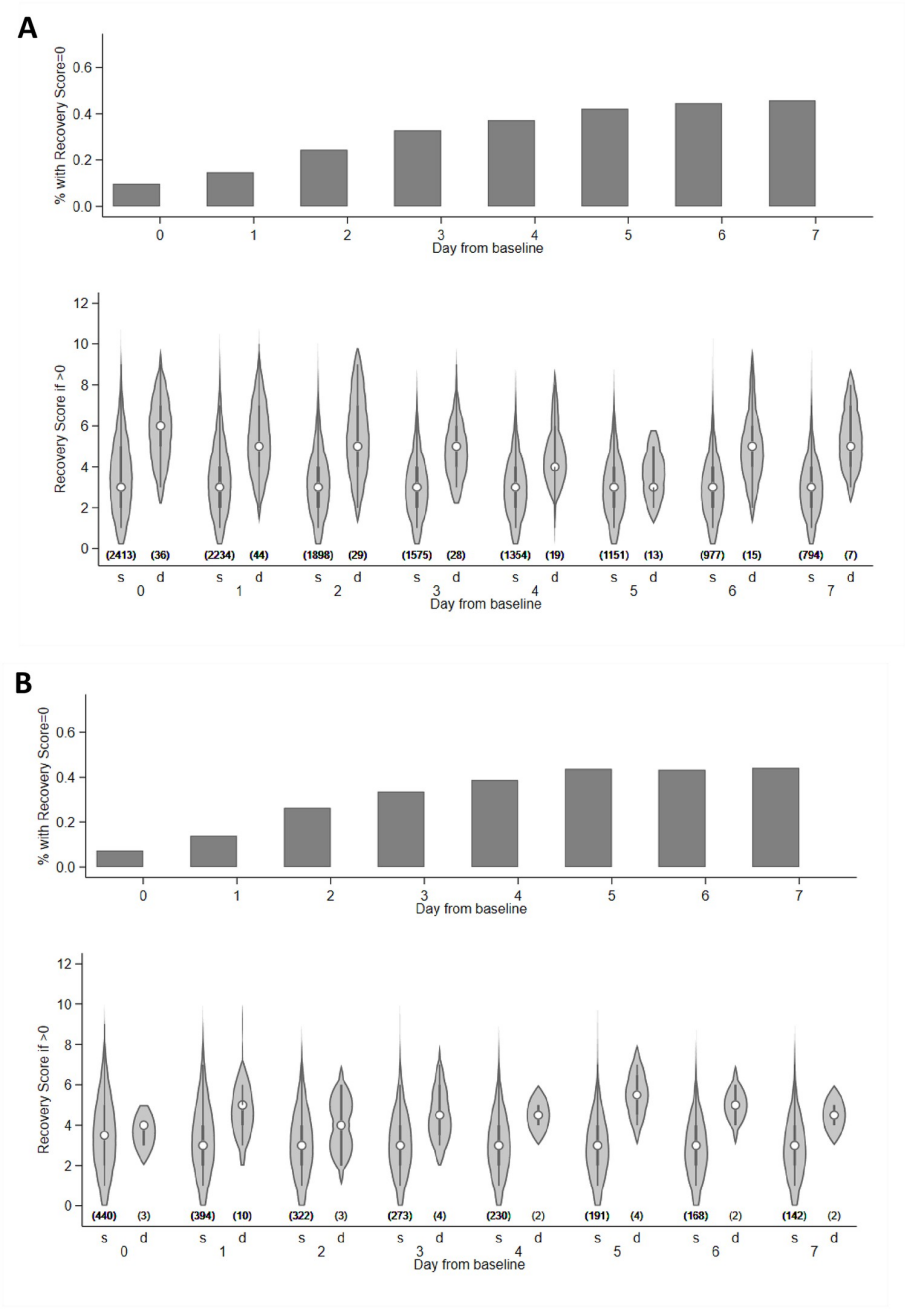
Distribution of the NeoSep Recovery Score. Distribution of the Recovery Score, based on clinical signs in the last 24 h, in the derivation (A) and validation (B) data. Numbers in parentheses: number of infants per group. s = survived, d = died the next day. Infants who died on same day are excluded for the relevant time points. The violin plots show the distribution of the Recovery Score as density plots plus median, interquartile range, and upper- and lower-adjacent values in infants with a score > 0.

**Fig 7:**
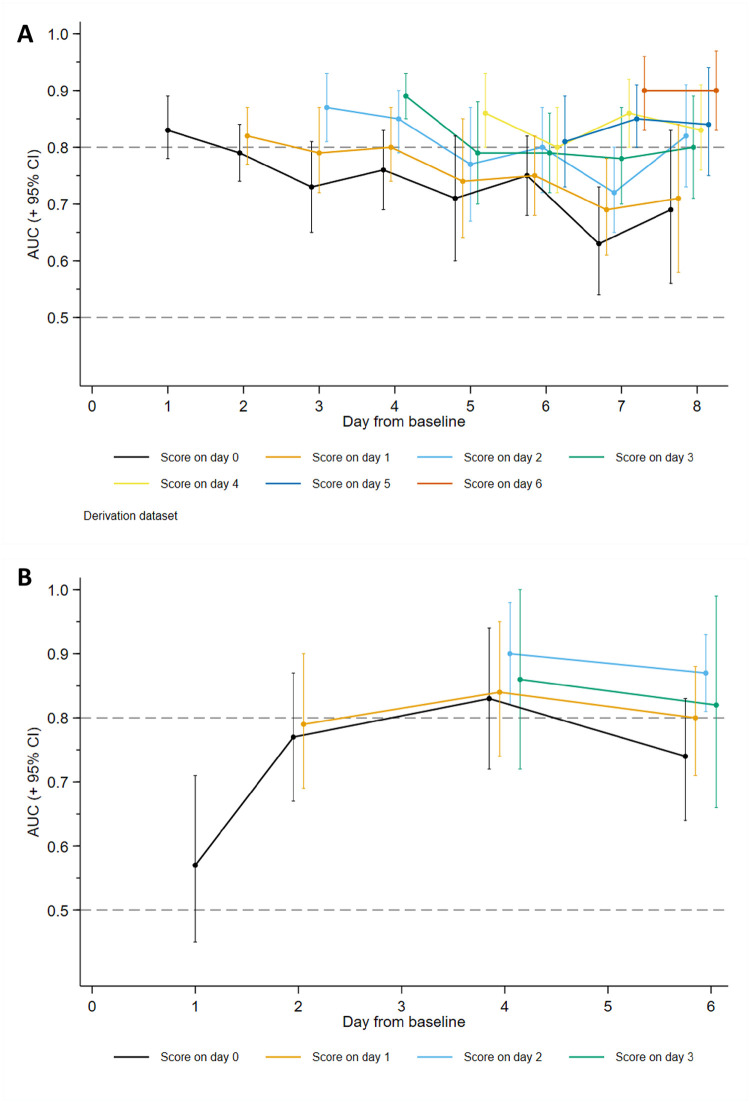
NeoSep Recovery Score: Time-updated area under the ROC curve. Time-updated area under the ROC curve (AUROCC) plus 95% CI in the derivation (A) and validation (B) data. Lines represent the trend in predictive value over time of the recovery score on a particular day for death in subsequent days. AUC (+ 95% CI) = area under the curve (+ 95% confidence interval), ROC = receiver operating characteristic.

Seven of the time-varying factors independently predicted mortality in the final model, including all the non-modifiable signs in the NeoSep Severity Score (respiratory support and the 5 clinical signs), plus cyanosis, which had not added independent information in the baseline model (*p* = 0.228) (Table 2). A NeoSep Recovery Score developed from these time-updated predictors had a maximum 11 points and discriminated well between infants who died or survived the next day (Fig 6A and 6B). The AUROC of the recovery score on one day, for predicting death the next day, ranged between 0.8 and 0.9 over the first week post baseline in the derivation sample (Fig 7A). As expected, it slowly decreased over subsequent days. AUROC over time in the validation sample was similar although based on fewer numbers (Fig 7B).

The NeoSep Recovery Score on day 2 had an AUROC for dying in the following 5 days of 0.82 (95% CI 0.78 to 0.85) and 0.85 (95% CI 0.78 to 0.93) in the derivation and validation samples, respectively. A score ≥4 was the most discriminative, whether this was an increase from baseline, lack of initial response, or an improvement from a higher score down to 4, with sensitivity and specificity of 0.74 (95% CI 0.64 to 0.82) and 0.74 (0.72 to 0.75) in the derivation, and 0.87 (0.60 to 0.98) and 0.76 (0.71 to 0.79) in the validation samples.

Twenty-nine infants (derivation: *n* = 27; validation: *n* = 2) died between day 3 and 7 despite having had a score <4 on day 2. Of these, 6 deaths were not classified as infection-related, 10 had a subsequent increase in score to ≥4 before dying, 9 had at least 2 unmodifiable risk factors included as predictors of mortality in the NeoSep Severity Score (most commonly congenital abnormalities and very low birth weight), and 1 had congenital varicella; the remaining 4 had a day 2 score of 2 or more.

Of note, the change in score from baseline to day 2 had poorer discrimination than the absolute score on day 2, with an AUROC for dying in the following 5 days of 0.68 (95% CI 0.63 to 0.73) and 0.62 (95% CI 0.48 to 0.77) in the derivation and validation samples, respectively. Combining change in score using various cut-offs with <4/≥4 absolute score on day 2 also did not improve discrimination (S26 Fig).

## Discussion

In this large hospital-based observational study in 11 countries across Africa, Asia, Europe, and Latin America, we observed wide variation in antibiotic prescribing beyond WHO recommendations for neonates and young infants with sepsis, with over 200 different empiric combinations, frequent empiric escalation, and rare de-escalation of therapy. Approximately 10% of pathogen-negative and 18% of pathogen-positive infants died. Gram-negative infections were associated with the majority of pathogen-positive deaths, and leading pathogens such as *K*. *pneumoniae*, *Acinetobacter* spp., and *S*. *aureus* were often resistant to WHO regimens. Mortality was independently associated with a range of factors, developed into a sepsis severity score which discriminated well between survivors and non-survivors at baseline. A recovery score was developed accounting for evolving clinical signs after baseline in order to assess response to antibiotics and also performed well at predicting death the following day. The NeoOBS study highlights the urgent need for clinical trials to inform antibiotic use for neonatal sepsis globally, describes the standard of care (SOC) against which novel antibiotic regimens should be compared, and provides severity and recovery scores that can be used for inclusion and antibiotic escalation criteria in future trial design.

Our data on antibiotic use are similar to recent point prevalence surveys in LMIC settings [16], but this study provides evidence on patterns of switching and escalation of therapy that is rarely available in the literature. A third of infants that had started on WHO-recommended regimens escalated to broader spectrum antibiotic regimens, and escalation overall was most commonly to a carbapenem. There was also considerable use of “carbapenem-sparing” regimens with “partial” ESBL and/or antipseudomonal activity that varied between centers (piperacillin-tazobactam, ceftazidime, and quinolone-based regimens, often in combination with amikacin). Evidence on carbapenem-sparing regimens is limited in neonates [27], but *Pseudomonas* infections were rare in NeoOBS and in the recent published literature [5,6]. Notably, a small but important proportion of infants in some sites received treatment for proven or suspected carbapenem-resistant infection with colistin, for which a recommended approach to dosing and combination therapy remains unclear, and CSF penetration and side effect profile are suboptimal [28]. Some countries are also using less widely used combinations such as cefoperazone/sulbactam, for which neonatal data are limited [29,30]. There was almost no de-escalation of therapy.

AMR was common and gram-negative pathogens (*Klebsiella* and *Acinetobacter)* were largely resistant to WHO-recommended regimens. Among gram positives, MRSA accounted for over half of *S*. *aureus* isolates. However, the extent to which increasing prevalence of AMR is associated with excess mortality, and whether this may be modifiable with different antibiotic treatment strategies is unclear in neonates and young infants. AMR has been demonstrated as an independent predictor of mortality in adults with bloodstream infections, including in LMIC settings [31]. However, data in neonates and young infants in many LMIC settings are scarce [15,32]. A limited number of largely retrospective observational studies have shown an increase in mortality particularly in association with resistant gram-negative infections [33–36], such as those due to organisms producing ESBL [37–40] and CROs [41–44], and some suggest an increase in mortality in the absence of appropriate antimicrobial therapy [33,45–48]. A recent large multicenter neonatal sepsis study (BARNARDS) demonstrated high resistance to ampicillin + gentamicin (97% and 70%, respectively) among gram-negative infections; however, analysis of the influence of antibiotic treatment on outcomes was confounded by country effects, and limited clinical data prevented adjusting for other important confounders [9]. Appropriate analyses of the causal relationship between discordant antibiotic treatment and mortality would need to consider baseline confounders as well as time-dependent confounding [49], but to our knowledge, this has not been done for neonatal sepsis in LMIC settings to an extent that could inform changes to global guidance [9,36,43,50,51]. Relevant analyses in the NeoOBS cohort are ongoing. Ultimately, evidence from randomized controlled trials is required to determine the most appropriate antibiotic regimens in different contexts.

The NeoSep Severity and Recovery Scores provided good discrimination. A score of 5 or higher at baseline was associated with 28-day mortality over 10%, and a recovery score 2 days after antibiotic initiation of 4 or higher had both sensitivity and specificity of 74% for mortality over the following 5 days. The NeoSep scores include clinical signs that have been designed to closely align with the WHO pSBI criteria (compared in S14 Table) used in recent community-based studies, including AFRINEST [52] and SATT [53,54]. However, the NeoSep scores include additional factors more relevant to hospital settings which offer further predictive value, such as clinical evidence of shock, and need for oxygen and/or respiratory support. Importantly, the study population also included a more heterogenous mix of both preterm (including <1,500 g) and term infants, with early and late onset, and community and healthcare-associated sepsis, and the Severity and Recovery Scores performed similarly across all these subgroups. Of note, convulsions were uncommon in the study (7%) and were not associated with mortality despite being a pSBI criteria for critical illness, potentially due to low power. Previous severity scores derived from hospital-based LMIC cohorts have been based on smaller studies assessing general illness severity rather than sepsis specifically [55–60]. High-income setting-based general illness severity scores are more widely used [55–57], and some have been assessed in small populations of septic neonates, but they are often unfeasible to apply globally [61,62]. The nSOFA score is a recent sepsis-specific score developed on 60 very low birth weight (<1,500 g) infants, but relies on measurement of parameters that are often unavailable in LMIC settings (e.g., use of inotropes/vasoactive drug use) [63]. The NeoSep scores are based on clinical variables that are relevant and feasible to collect globally across a range of LMIC hospital settings.

There are limitations to the generalizability of the data. The sites were selected to be heterogenous, varying in patient volume and case-mix, and with wide variation in levels of supportive care. Sites also varied in their ability to screen, using different screening schedules depending on staff levels and number of patients, particularly during nights or weekends. Given the very large numbers of infants presenting to many of the sites (see S1 Table), including out of hours, project funding was not available to prospectively screen and record the status of all admitted infants and reasons for not being enrolled. We would note, however, that the design enrolled participants who could feasibly be recruited to an antibiotic trial. Most sites in NeoOBS represented secondary or tertiary hospitals in urban settings, in some cases receiving referrals after prior treatment in other hospitals. In many cases, these facilities provided a higher level of care than is typical in many low-resource settings. AMR may be more important and a wider range of antibiotics may be available in some of the settings in this study compared to district hospital settings [64,65]. This is an important bias in most studies on AMR in low-resource settings, where the need for high-quality microbiology means certain settings are overrepresented.

A significant proportion (36.8%) of infants had previously received antibiotics before the new sepsis episode, which may influence culture yield and pathogen characteristics. Selection bias was also possible beyond site selection, with prospective recruitment potentially leading to a milder phenotype as infants who die rapidly with sepsis are more difficult to enroll, possibly leading to underestimation of mortality. There was wide variation in mortality between sites, highlighting major limitations of basing antibiotic recommendations on observational data alone. Analyses to determine the impact of antibiotic use on outcomes are confronted with important biases [49], including the influence of initial sepsis severity on antibiotic choice and timing of first administration, antibiotic availability and affordability, inter-site population heterogeneity, local guidelines and microbiology, and varying levels of supportive care.

In developing the NeoSep Severity and Recovery Scores, we attempted to account for inter-site variation by using multivariable models with site-level random-effects, excluding factors with a high proportion of missing values. We also examined performance of the Severity Score across different sites in high-, middle-, and low-income contexts and demonstrated reproducible risk trends across all settings. Of note, the scores were validated on a predefined reserved 15% internal sample, including only 42 deaths (and relatively few infants with high-risk scores). External validation would strengthen the applicability of the scores.

In the context of increasing resistance to WHO-recommended therapy for neonatal sepsis in LMIC settings, and a lack of evidence to guide optimal management due to the limitations of observational data, further randomized antibiotic trials are urgently needed. This study has demonstrated that there is no single accepted “standard of care” in neonatal sepsis in LMIC hospital settings, and any novel regimens will need to be compared to multiple widely used empiric regimens including current WHO-recommended guidance. NeoOBS data, including the severity and recovery scores, have informed the design of the NeoSep1 trial (ISRCTN 48721236) which will use a network meta-analytic approach to rank novel off-patent antibiotic combinations compared to WHO-recommended and other commonly used regimens, combined with a SMART (Sequential Multiple Assessment Randomized Trial) design to allow randomization to both empiric first- and second-line treatment [66].

## Supporting information

S1 Appendix Supplementary methods: Sample size determination. (PDF)

S1 STROBE checklist STROBE checklist. (PDF)

S1 TRIPOD checklist TRIPOD checklist. (DOCX)

S1 Dataset Data used to develop NeoSep Severity Score. (XLSX)

S2 Dataset Data used to develop NeoSep Recovery Score. (XLSX)

S1 Fig Clinical and laboratory sepsis enrolment criteria. (PDF)

S2 Fig Study flow diagram. (PDF)

S3 Fig Birth weight by site. Boxes show 25th percentile (lower hinge), median (line), and 75th percentile (upper hinge); whiskers show lower and upper adjacent values as defined by Tukey.
(TIF)

S4 Fig Hospitalized since birth vs. admitted, by site. Proportion of infants hospitalized since birth and proportion admitted from home/community, per site.
(TIF)

S5 Fig Postnatal age at enrolment by site. Boxes show 25th percentile (lower hinge), median (line), and 75th percentile (upper hinge); whiskers show lower and upper adjacent values as defined by Tukey.
(TIF)

S6 Fig Frequency of supportive measures across sites. This figure shows the number of sites (out of 19 overall) per percentage category of participants receiving a particular supportive measure at enrolment.
(TIF)

S7 Fig Prevalence of clinical signs/respiratory support over time. Prevalence of clinical symptoms that are part of the NeoSep Severity Score and use of respiratory support over time in infants on IV antibiotics.
(TIF)

S8 Fig Prevalence of clinical signs at enrolment (≤5%). Prevalence of less common clinical symptoms at enrolment (≤5%).
(TIF)

S9 Fig Time to start IV antibiotics after baseline culture. Time to first new IV antibiotic, by antibiotic exposure at enrolment. *P*-value derived from a log-rank test.
(TIF)

S10 Fig Group 1 antibiotics. Group 1: First-line WHO-recommended penicillin-based regimen (Access): Most common antibiotics used as initial regimen.
(TIF)

S11 Fig Group 2 antibiotics. Group 2: Third-generation cephalosporin-based WHO regimens (“Low” Watch), most common antibiotics used as initial regimen.
(TIF)

S12 Fig Group 3 antibiotics. Group 3: Regimens with partial anti-extended-spectrum beta-lactamase (ESBL) or pseudomonal activity (“Medium” Watch), most common antibiotics used as initial regimen. Pip/taz = piperacillin/tazobactam.
(TIF)

S13 Fig Group 4 antibiotics. Group 4: Carbapenems (“High” Watch), most common antibiotics used as initial regimen.
(TIF)

S14 Fig Group 5 antibiotics. Group 5: Reserve antibiotics targeting carbapenem resistant organisms (e.g., colistin), most common antibiotics used as initial regimen.
(TIF)

S15 Fig “Other” group antibiotics. Other antibiotics used as initial regimen.
(TIF)

S16 Fig Antibiotic use on day 1 and on day 4 post baseline. (TIF)

S17 Fig Antibiotic use on day 4, by initial regimen. (TIF)

S18 Fig Daily antibiotic regimens, by initial regimen and overall. Cross-sectional analysis. Ignoring (unknown) treatment after transfer/readmission to another hospital.
(TIF)

S19 Fig Days on antibiotics during follow-up. Peak at days 27/28 due to infants still on IV antibiotics at the end of follow-up.
(TIF)

S20 Fig Prevalence of pathogens isolated from baseline blood culture. Prevalence of pathogens in participants with a baseline blood culture.
(TIF)

S21 Fig Pathogens isolated from baseline blood culture, by site. (TIF)

S22 Fig Time to death, by time to start new IV antibiotics after baseline culture. *P*-value derived from log-rank test.
(TIF)

S23 Fig NeoSep Severity Score performance in subgroups. (A) Mortality (95% CI) in risk groups based on the Severity Score and pathogen/no pathogen at baseline. (B) Mortality (95% CI) in risk groups based on the Severity Score and preterm/term birth.
(TIF)

S24 Fig Comparison between WHO pSBI signs and NeoSep Severity Score for predicting 28-day mortality. Score based on WHO signs of Clinical Severe Infection, compared with a modified NeoSep Severity Score excluding unmodifiable infant/birth characteristics. pSBI = Possible serious bacterial infection.
(PDF)

S25 Fig NeoSep Recovery Score over time (cross-sectional analysis). Dashed line: for each score point, indicates those babies who eventually die on antibiotics; * Restart of antibiotics ignored.
(TIF)

S26 Fig NeoSep Recovery Score on day 2 and change in score from baseline to day 2. (A) Derivation data. (B) Validation data.
(TIF)

S1 Table Candidate factors for the development of the NeoSep Severity Score. * Severe chest wall in-drawing, increased requirement for oxygen or respiratory support; ͳ Observed or reported, including feeding intolerance.
(PDF)

S2 Table Numbers of births and admissions in each site (per 6 months). (PDF)

S3 Table Congenital anomalies. Proportion of infants with congenital anomalies at enrolment.
(PDF)

S4 Table Laboratory results from blood at baseline. IQR = interquartile range, CRP = C-reactive protein.
(PDF)

S5 Table Most common initial antibiotic regimens. Most common initial regimens in infants who started new IV antibiotics within 24 h from baseline blood culture.
(PDF)

S6 Table Most common initial antibiotic regimens, by time from admission. HAI = healthcare-associated infection (occurring ≥48 h after admission).
(PDF)

S7 Table Factors associated with non-WHO–recommended regimens (Groups 3–5). Note: OR = odds ratio, derived from logistic regression models adjusted for center. Time in hospital was analyzed as continuous variable using fractional polynomials with powers 1 1. For illustrative reasons, in this table we report odds ratios for specific values.
(PDF)

S8 Table Organisms isolated from blood at baseline, by time from admission. Note: * Organisms isolated in only 1 infant per group.
(PDF)

S9 Table Susceptibility result for pathogens in baseline blood culture. Note: S = susceptible; I = intermediate; R = resistant. Denominators across drugs variables due to available susceptibility results.
(PDF)

S10 Table Organisms isolated from CSF in first 7 days from baseline. Note: *n* = 73 with positive CSF culture in first 7 days.
(PDF)

S11 Table Pathogens and mortality. Note: Results are hazard ratio (95% confidence interval). All models are adjusted for site (random effect). * Adjusted for birth weight, gestational age, time in hospital, congenital anomalies, and site; ** adjusted for all factors in NeoSep Severity Score (as in * plus abdominal distension, difficulty in feeding, evidence of shock, lethargy/no movement, temperature, and level of respiratory support); *** including Coagulase-negative staphylococcus. ref. = reference category.
(PDF)

S12 Table Characteristics of participants in derivation and validation samples. ^1^ Outcome for developing NeoSep Severity Score; ^2^ outcome for developing NeoSep Recovery Score.
(PDF)

S13 Table Unadjusted analyses. Notes: This table shows results of unadjusted Cox proportional hazards models for selected factors with site-level random effects: (1) for a baseline NeoSep Severity Score to predict 28-day mortality from factors known at sepsis presentation; and (2) for a NeoSep Recovery Score to predict the daily risk of death while treated with IV antibiotics from daily updated assessments of clinical status.^‡^ Birth weight and temperature were analyzed as continuous variables using fractional polynomials with powers −2 for birth weight, and powers 2 2 for temperature. For illustrative reasons, in this table we report HRs for specific values. Shaded factors: deliberately not included in model (unmodifiable factors excluded from recovery score: birth weight, time in hospital, gestational age, congenital anomalies). CPAP = Continuous Positive Airway Pressure, BiPAP = Bilevel Positive Airway Pressure, HFNC = High Flow Nasal Cannula, HR = hazard ratio, CI = confidence interval, coef = coefficient.
(PDF)

S14 Table Comparison of factors in NeoSep Sepsis Severity score with existing scores and sepsis criteria. CRIB = Clinical Risk Index for Babies Score; NMR score = Neonatal Mortality Risk score; SAWS = Simplified Age-Weight-Sex score; HR = Heart Rate; LBW = Low birth weight; SpO_2_ = Oxygen Saturation.
(PDF)

## Abbreviations

AMR: antimicrobial resistance
AUROC: area under the receiver operator curve
CLSI: Clinical and Laboratory Standard Institute
CRO: carbapenem-resistant organism
CSF: cerebrospinal fluid
EMLc: Essential Medicine List for Children
EQA: external quality assurance
ESBL: extended-spectrum beta-lactamase
EUCAST: European Committee on Antimicrobial Susceptibility Testing
FP: fractional polynomial
HAI: healthcare-associated infection
LMIC: low- and middle-income country
MDR: multidrug resistant
pSBI: possible serious bacterial infection
SMART: Sequential Multiple Assessment Randomized Trial
SOC: standard of care
WHO: World Health Organization
