# Supplementary material for: Patterns of antibiotic use, pathogens, and prediction of mortality in hospitalized neonates and young infants with sepsis: A global neonatal sepsis observational cohort study (NeoOBS)
Source: PLoS Med. 2023 Jun 8;20(6):e1004179. doi: 10.1371/journal.pmed.1004179 (PMC10249878; doi:10.1371/journal.pmed.1004179)
Supplement: S1 Appendix — (PDF) [file pmed.1004179.s001.pdf]

S1 Appendix. Supplementary methods.

Data monitoring

On-site study initiation visits were performed and online training was made available via The Global Health Network (URL: https://tghn.org/) for study processes including recruitment, data collection and entry, and laboratory procedures, standard procedures for measuring clinical signs, and blood culture sampling. Translation of study documents and manual of procedures into local languages was performed where necessary. Routine data queries were raised continuously throughout the study and data was verified off-site against paper CRFs uploaded securely in REDCap™ for up to 10% of total participants per site and against source documentation on site for up to an additional 10% of total participants.

Sample size determination, as defined in the NeoOBS study protocol

In order to ensure a population which represents the diversity of neonatal sepsis management, the study will be conducted in a purposive sample of 4-5 centres from each of the 4 WHO regions with a majority of lower and middle-income countries, selected to represent intensity of available care and admission intensity.
Within each site, recruiting 200 infants with significant sepsis provides >80% power to detect differences in the estimated mortalities of 50% for blood culture positive cases vs. 10% for blood culture negative cases, assuming an inflation factor of 15% to allow for loss to follow-up (2 sided alpha = 0.05). This is based on preliminary data from feasibility studies and the DenIS study suggesting that a conservative estimate of blood culture positivity rate (per admission to NICU) is around 5% (i.e. 1 in 20 admissions to a neonatal unit results in a positive blood culture), and observing 50% mortality in blood-culture positive vs. 10% in blood culture negative. If a greater proportion of infants screened for clinical sepsis are blood culture positive, power to detect these difference increases (e.g. to >95% if 14% are blood culture positive) and equivalently power to detect smaller differences within sites also increases.

Dealing with sites in the statistical analysis

There were considerable differences between sites in many factors. This raised the question of how to deal with it in the analysis, for example, a) weighting each baby equally, b) weighting each site equally, or c) using techniques such as inverse variance weighting.
For the description of baseline characteristics, we presented raw numbers, that is weighted each baby equally (e.g. number of positive infants divided by the total number of infants across all sites). This deemed appropriate because, by design, the sample size was relatively homogeneous across sites, targeting approximately 200 babies in each site with 15 of 19 participating sites recruiting this number. Therefore, practically, the difference between the three methods above are small. Whilst statistically the third method has advantages, what it loses is transparency – for example all baseline tables would either need to have both the raw number and percentage for transparency plus the estimated % combined across sites using meta-analytic techniques (which would be close but not exactly the same, potentially causing confusion), or the latter only with no numbers (when many reporting guidelines request numbers accompany percentages).
The second consideration relates to what population generalisation is aimed at – another site or another baby. When the study was set up, it was recognised that neonatal sepsis was a condition with very diverse presentations and referral pathways (e.g. % inborn vs outborn), dependent on geography (including both background resistance rates but also degree of urbanisation and hospital level) as well as maternal factors and baby factors, both at birth and at presentation. Whilst sites were selected globally to deliberately represent

diverse regions and hospitals serving varying populations of neonates (% in-born/out-born etc.), the clear importance of many baby-level factors on mortality meant that it was felt more appropriate to consider the unit at which we summarised characteristics as the baby, rather than the site, recognising that in practice there is no perfect solution given the underlying diversity of the condition, and lack of an obvious “reference” to weight back to. The decision to use site as a shared frailty term in the Cox model was driven by the need to adjust these models for unmeasured background management (including staffing levels, infection prevention and control amongst many others), which will have a major impact on mortality not otherwise captured in maternal and baby characteristics.
