# Supplementary material for: Patterns of antibiotic use, pathogens, and prediction of mortality in hospitalized neonates and young infants with sepsis: A global neonatal sepsis observational cohort study (NeoOBS)
Source: PLoS Med. 2023 Jun 8;20(6):e1004179. doi: 10.1371/journal.pmed.1004179 (PMC10249878; doi:10.1371/journal.pmed.1004179)
Supplement: S1 STROBE checklist — (PDF) [file pmed.1004179.s002.pdf]

S1 Checklist: STROBE Checklist

	Item No	Recommendation	Section and paragraph number
**Title and abstract**	1	(*a*) Indicate the study's design with a commonly used term in the title or the abstract	Title
		(*b*) Provide in the abstract an informative and balanced summary of what was done and what was found	Abstract
**Introduction**			
Background/rationale	2	Explain the scientific background and rationale for the investigation being reported	Introduction, paragraphs 1-3
Objectives	3	State specific objectives, including any prespecified hypotheses	Introduction, paragraph 3
**Methods**			
Study design	4	Present key elements of study design early in the paper	Title;<br>Introduction, paragraph 3
Setting	5	Describe the setting, locations, and relevant dates, including periods of recruitment, exposure, follow-up, and data collection	Methods, paragraph 1<br>S1 Table
Participants	6	(*a*) Give the eligibility criteria, and the sources and methods of selection of participants. Describe methods of follow-up	Methods, paragraphs 2+ 4
		(*b*) For matched studies, give matching criteria and number of exposed and unexposed	n/a
Variables	7	Clearly define all outcomes, exposures, predictors, potential confounders, and effect modifiers. Give diagnostic criteria, if applicable	Methods, paragraphs 4-13
Data sources/ measurement	8	For each variable of interest, give sources of data and details of methods of assessment (measurement). Describe comparability of assessment methods if there is more than one group	Methods, paragraphs 4-8
Bias	9	Describe any efforts to address potential sources of bias	Methods, paragraphs 4-5
Study size	10	Explain how the study size was arrived at	S1 Appendix: Sample size determination
Quantitative variables	11	Explain how quantitative variables were handled in the analyses. If applicable, describe which groupings were chosen and why	Methods/Statistical analysis, paragraphs 2+5
Statistical methods	12	(*a*) Describe all statistical methods, including those used to control for confounding	Methods/Statistical analysis, paragraphs 1-5

		(*b*) Describe any methods used to examine subgroups and interactions	Methods/Statistical analysis, paragraph 2
		(*c*) Explain how missing data were addressed	Methods/Statistical analysis, paragraph 2
		(*d*) If applicable, explain how loss to follow-up was addressed	Methods/Statistical analysis, paragraphs 1,2,4
		(*e*) Describe any sensitivity analyses	n/a
**Results**			
Participants	13*	(a) Report numbers of individuals at each stage of study—e.g. numbers potentially eligible, examined for eligibility, confirmed eligible, included in the study, completing follow-up, and analysed	S2 Figure
		(b) Give reasons for non-participation at each stage	-
		(c) Consider use of a flow diagram	S2 Figure
Descriptive data	14*	(a) Give characteristics of study participants (eg demographic, clinical, social) and information on exposures and potential confounders	Results, paragraphs 1-3; S3-8 Figures
		(b) Indicate number of participants with missing data for each variable of interest	Results, paragraphs 4, 10, 14
		(c) Summarise follow-up time (eg, average and total amount)	Results, paragraph 14
Outcome data	15*	Report numbers of outcome events or summary measures over time	Results, paragraph 14
Main results	16	(*a*) Give unadjusted estimates and, if applicable, confounder-adjusted estimates and their precision (eg, 95% confidence interval). Make clear which confounders were adjusted for and why they were included	Results, paragraph 14 Table 2
		(*b*) Report category boundaries when continuous variables were categorized	-
		(*c*) If relevant, consider translating estimates of relative risk into absolute risk for a meaningful time period	-
Other analyses	17	Report other analyses done—eg analyses of subgroups and interactions, and sensitivity analyses	Results, paragraphs 15-22
**Discussion**			

Key results	18	Summarise key results with reference to study objectives	Discussion, paragraph 1
Limitations	19	Discuss limitations of the study, taking into account sources of potential bias or imprecision. Discuss both direction and magnitude of any potential bias	Discussion, paragraphs 5-7
Interpretation	20	Give a cautious overall interpretation of results considering objectives, limitations, multiplicity of analyses, results from similar studies, and other relevant evidence	Discussion, paragraphs 4,8
Generalisability	21	Discuss the generalisability (external validity) of the study results	Discussion, paragraphs 5,6,7
**Other information**			
Funding	22	Give the source of funding and the role of the funders for the present study and, if applicable, for the original study on which the present article is based	With submission
