# Supplementary material for: Patterns of antibiotic use, pathogens, and prediction of mortality in hospitalized neonates and young infants with sepsis: A global neonatal sepsis observational cohort study (NeoOBS)
Source: PLoS Med. 2023 Jun 8;20(6):e1004179. doi: 10.1371/journal.pmed.1004179 (PMC10249878; doi:10.1371/journal.pmed.1004179)
Supplement: S1 Fig — (PDF) [file pmed.1004179.s006.pdf]

S1 Fig. Clinical and laboratory sepsis enrolment criteria for the NeoOBS study.

Clinical criteria	Laboratory criteria
• Grunting<br>• Apnoea<br>• Abnormal heart rate (>180/min or <100/min)<br>• Severe chest in-drawing or increased oxygen requirement or need for respiratory support<br>• Abnormal temperature (>37.5°C or <36.5°C)<br>• Capillary refill time (CRT) >3 sec or mottled skin or other evidence of shock<br>• Irritability<br>• Convulsions<br>• Abdominal distension<br>• Abnormal posturing<br>• Hypotonia/floppiness<br>• Lethargy or drowsiness<br>• Cyanosis<br>• Bulging fontanelle<br>• No movement or movement only when stimulated<br>• Difficulty feeding or feeding intolerance<br>• Multiple or severe skin pustules<br>• Petechial rash<br>• Pus from umbilical stump	• White blood cells (WBC) < 4.0 or > 20.0 x 10^9 cells/L<br>• Absolute neutrophil count <1.5 x 10^9 cells/L<br>• Immature-to-total (IT) polymorph ratio of > 0.2<br>• C-reactive protein >10 mg/L or >1 mg/dL<br>• Acidosis: base excess (BE) < −10 mmol/L or blood lactate > 2 mmol/L<br><br>To be identified with significant sepsis, infants must meet at least TWO criteria, ONE of which must be clinical
