# Supplementary figures and images for: Patterns of antibiotic use, pathogens, and prediction of mortality in hospitalized neonates and young infants with sepsis: A global neonatal sepsis observational cohort study (NeoOBS)
Source: PLoS Med. 2023 Jun 8;20(6):e1004179. doi: 10.1371/journal.pmed.1004179 (PMC10249878; doi:10.1371/journal.pmed.1004179)

**S2 Fig. Study flow diagram.**

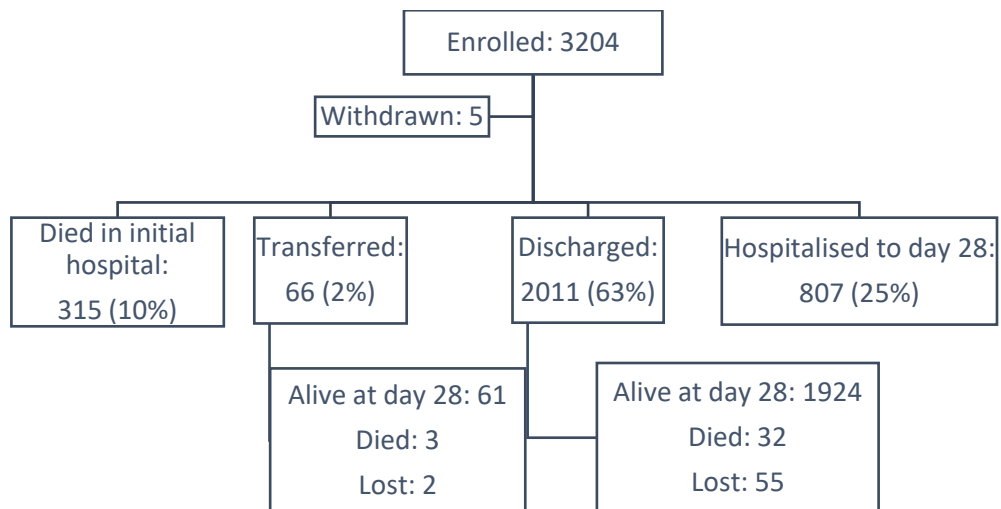

Supplement: S2 Fig — (PDF) [file pmed.1004179.s007.pdf]

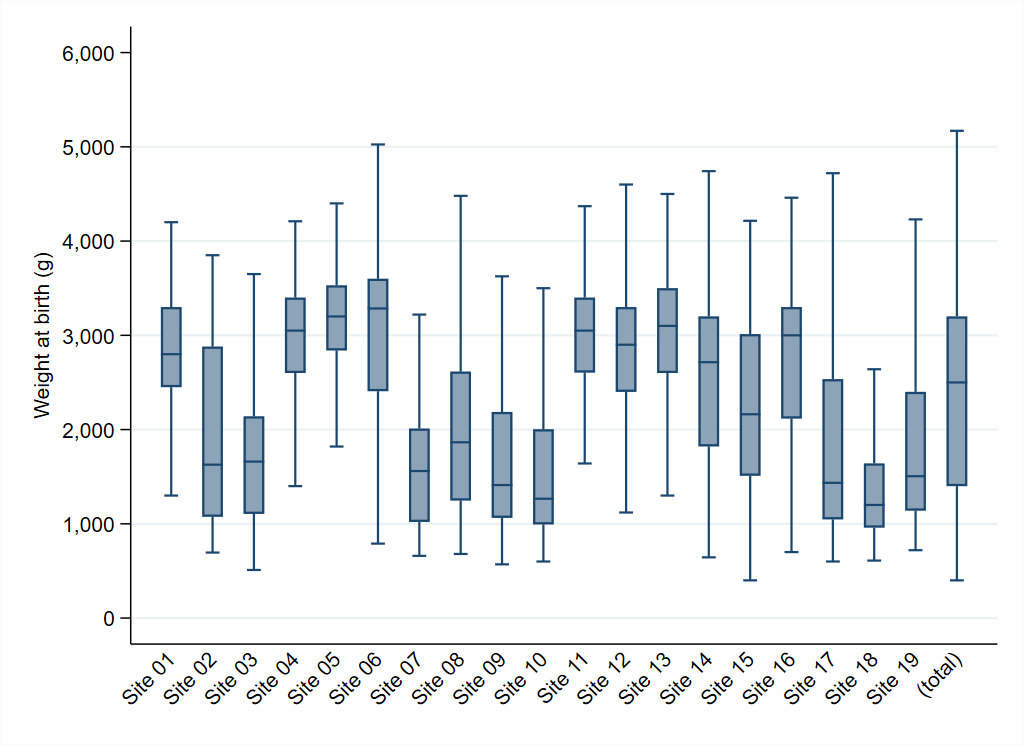

Supplement: S3 Fig — Boxes show 25th percentile (lower hinge), median (line), and 75th percentile (upper hinge); whiskers show lower and upper adjacent values as defined by Tukey. (TIF) [file pmed.1004179.s008.tif]

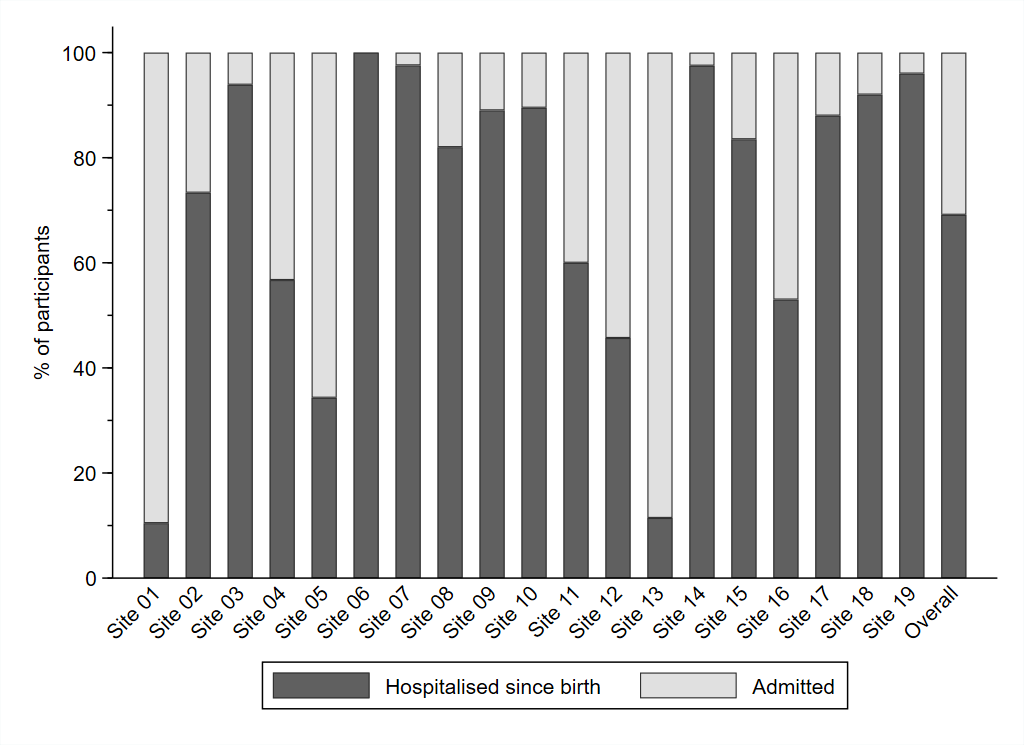

Supplement: S4 Fig — Proportion of infants hospitalized since birth and proportion admitted from home/community, per site. (TIF) [file pmed.1004179.s009.tif]

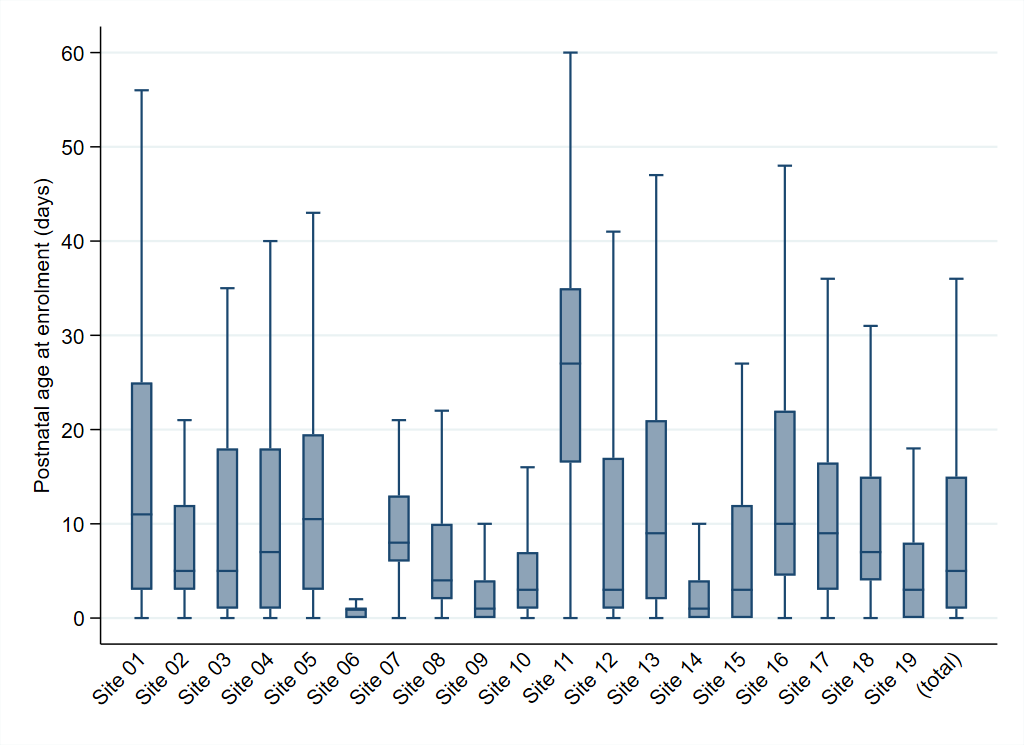

Supplement: S5 Fig — Boxes show 25th percentile (lower hinge), median (line), and 75th percentile (upper hinge); whiskers show lower and upper adjacent values as defined by Tukey. (TIF) [file pmed.1004179.s010.tif]

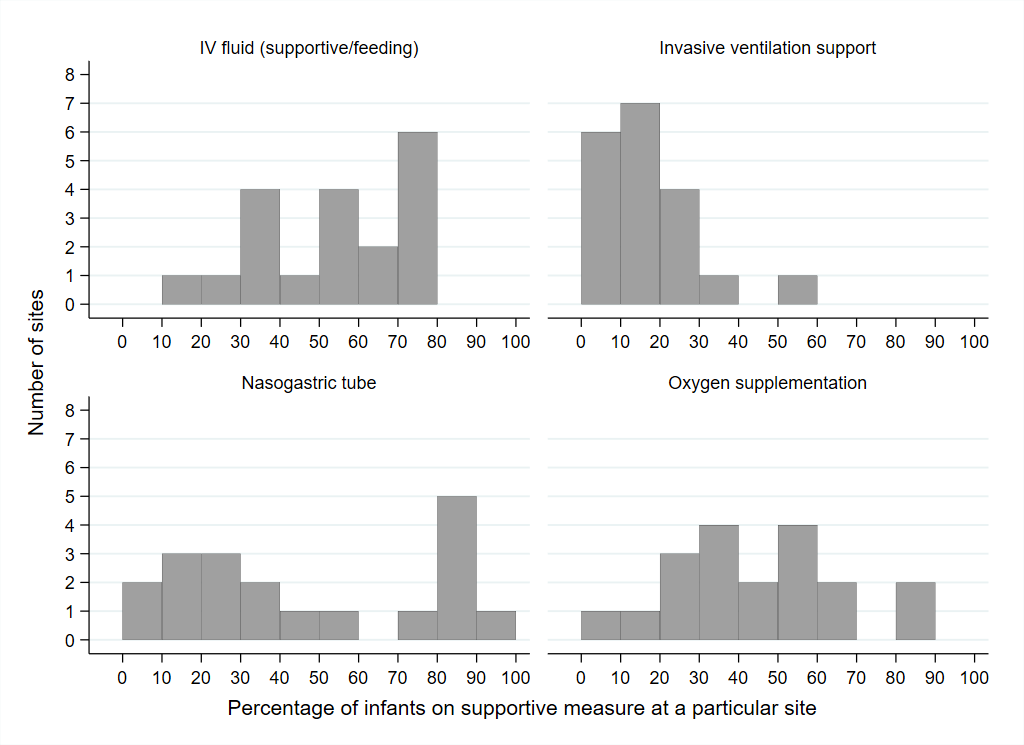

Supplement: S6 Fig — This figure shows the number of sites (out of 19 overall) per percentage category of participants receiving a particular supportive measure at enrolment. (TIF) [file pmed.1004179.s011.tif]

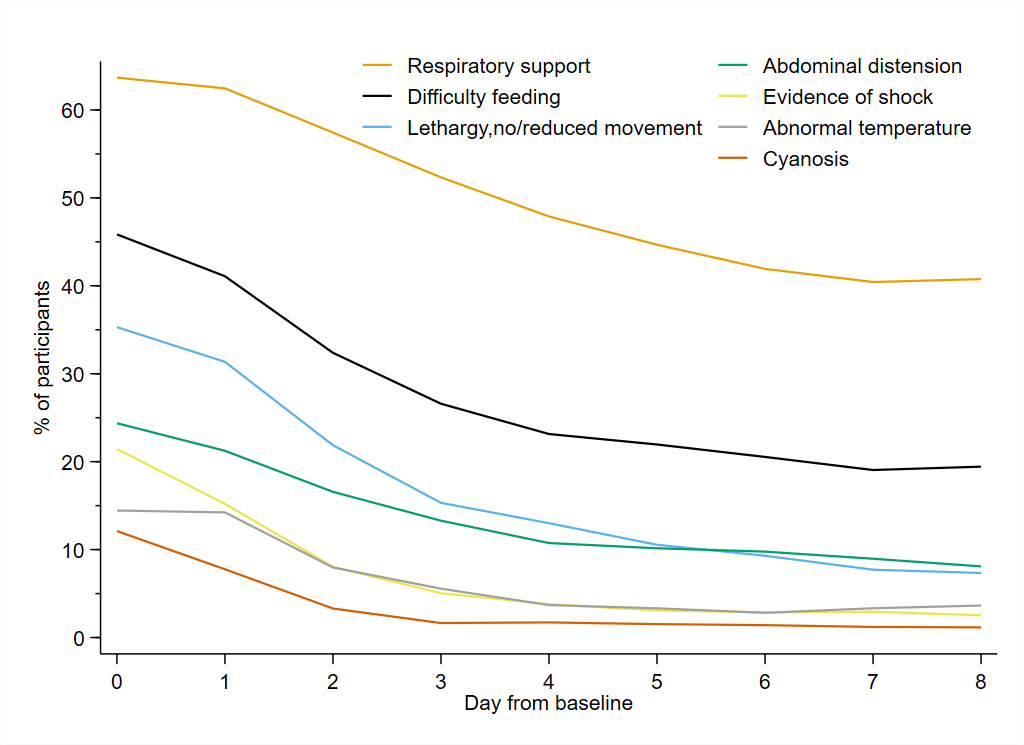

Supplement: S7 Fig — Prevalence of clinical symptoms that are part of the NeoSep Severity Score and use of respiratory support over time in infants on IV antibiotics. (TIF) [file pmed.1004179.s012.tif]

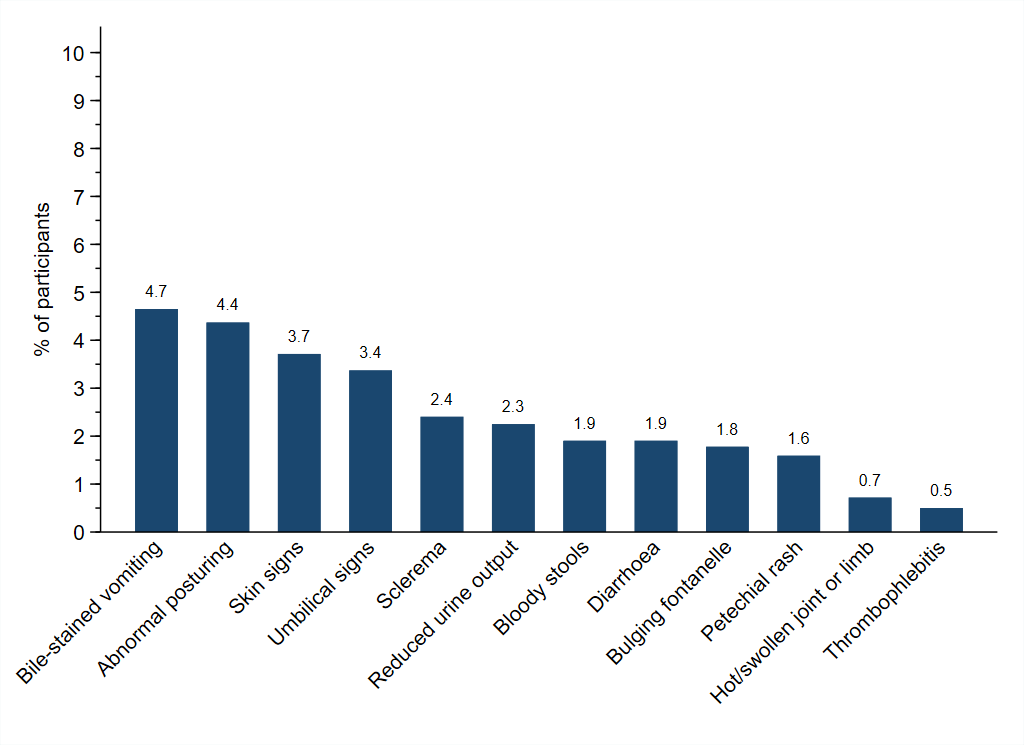

Supplement: S8 Fig — Prevalence of less common clinical symptoms at enrolment (≤5%). (TIF) [file pmed.1004179.s013.tif]

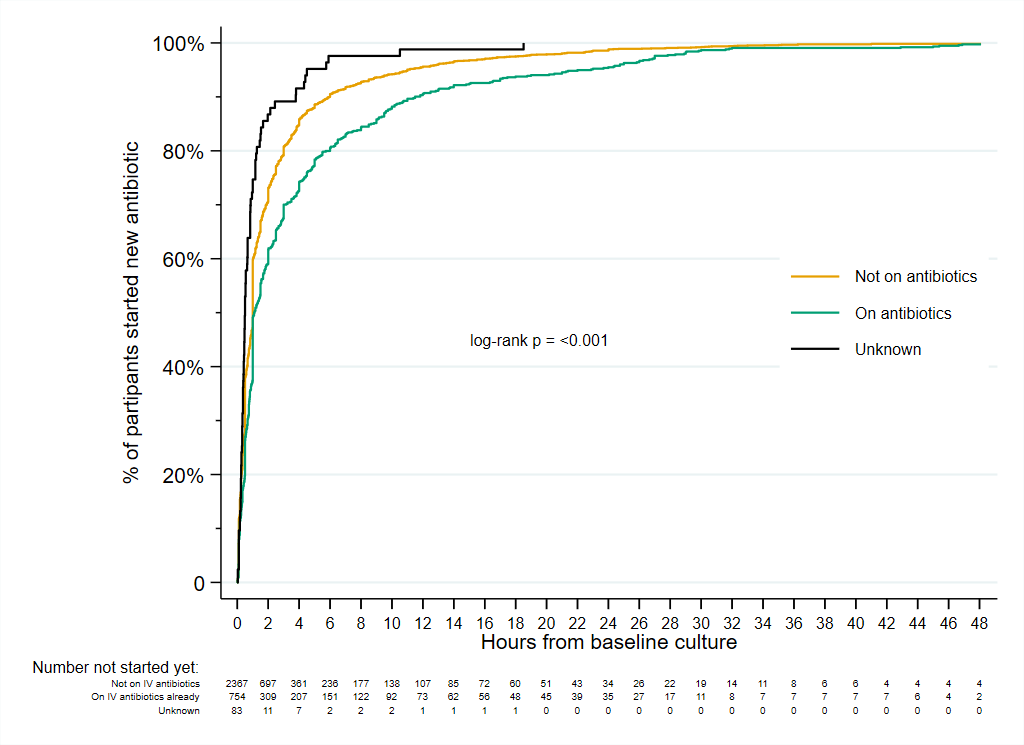

Supplement: S9 Fig — Time to first new IV antibiotic, by antibiotic exposure at enrolment. P-value derived from a log-rank test. (TIF) [file pmed.1004179.s014.tif]

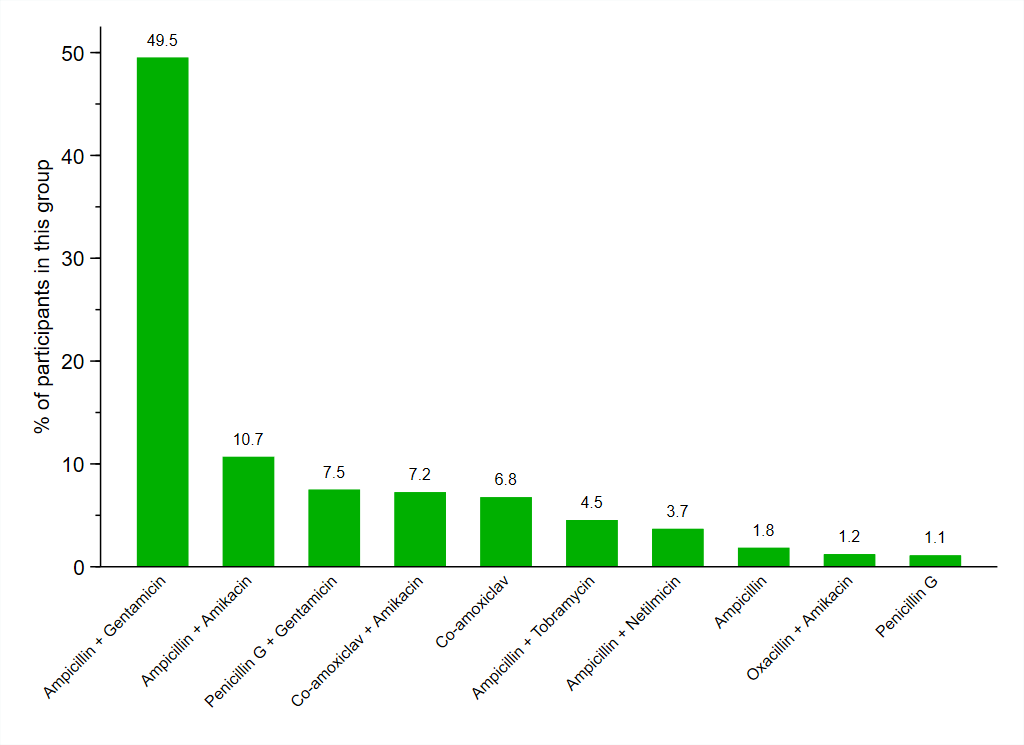

Supplement: S10 Fig — Group 1: First-line WHO-recommended penicillin-based regimen (Access): Most common antibiotics used as initial regimen. (TIF) [file pmed.1004179.s015.tif]

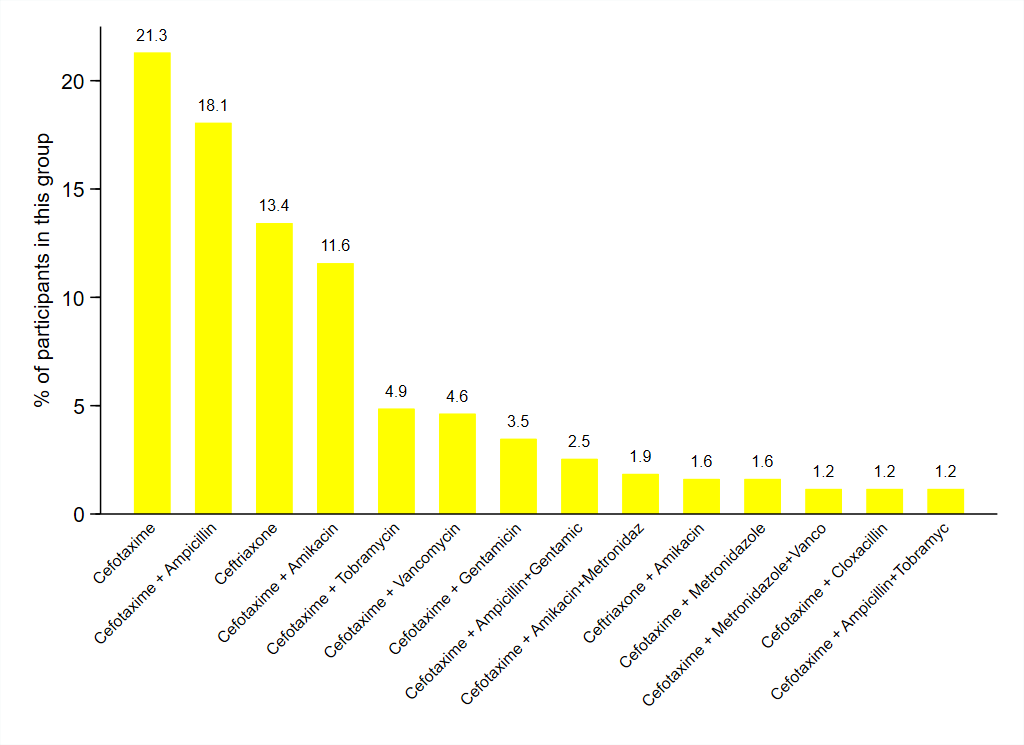

Supplement: S11 Fig — Group 2: Third-generation cephalosporin-based WHO regimens (“Low” Watch), most common antibiotics used as initial regimen. (TIF) [file pmed.1004179.s016.tif]

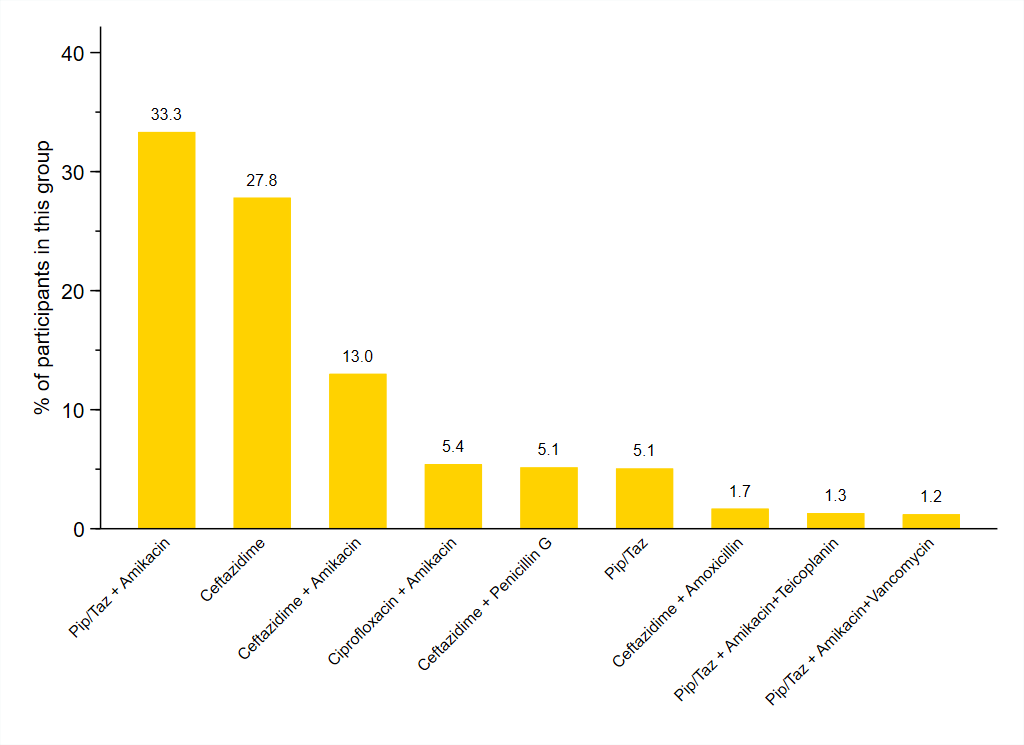

Supplement: S12 Fig — Group 3: Regimens with partial anti-extended-spectrum beta-lactamase (ESBL) or pseudomonal activity (“Medium” Watch), most common antibiotics used as initial regimen. Pip/taz = piperacillin/tazobactam. (TIF) [file pmed.1004179.s017.tif]

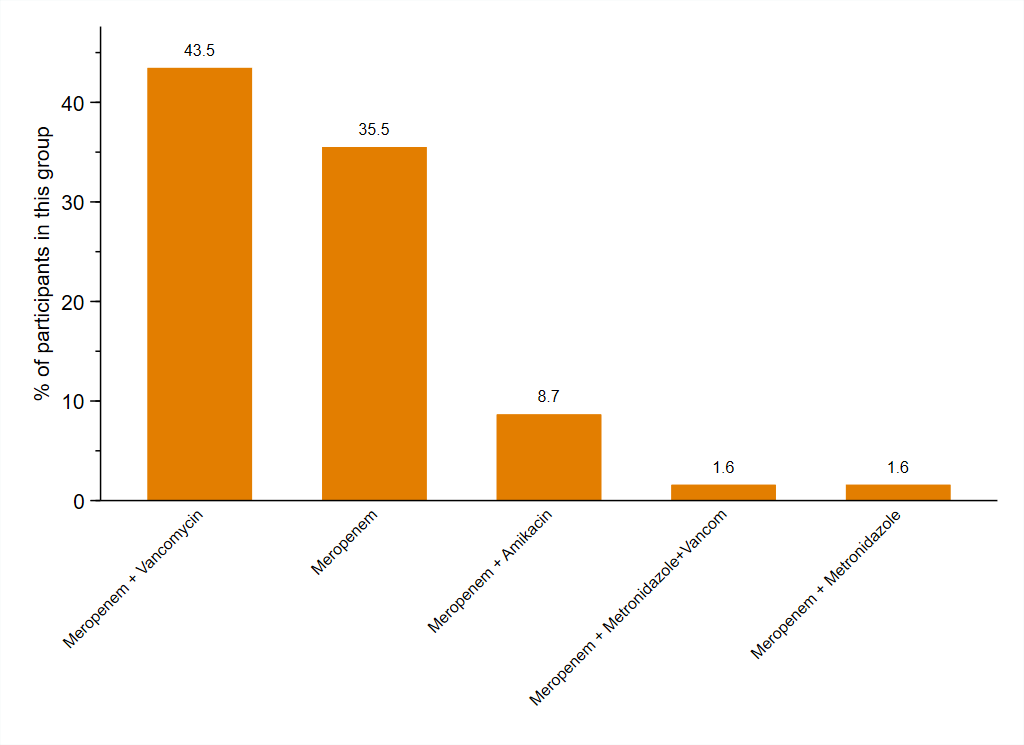

Supplement: S13 Fig — Group 4: Carbapenems (“High” Watch), most common antibiotics used as initial regimen. (TIF) [file pmed.1004179.s018.tif]

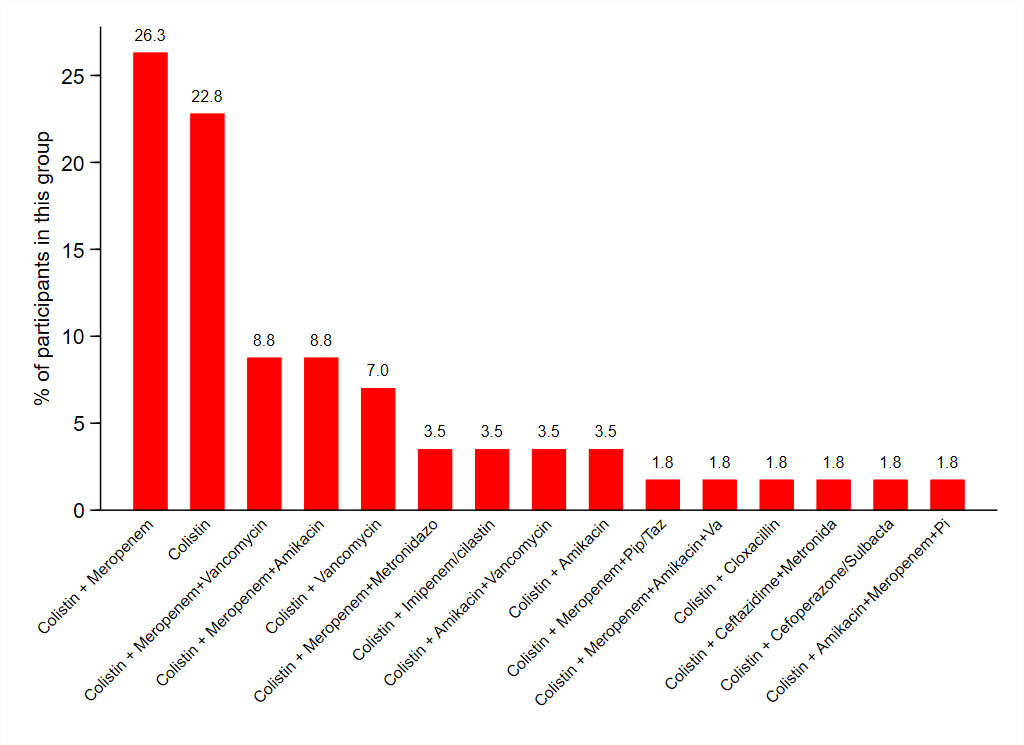

Supplement: S14 Fig — Group 5: Reserve antibiotics targeting carbapenem resistant organisms (e.g., colistin), most common antibiotics used as initial regimen. (TIF) [file pmed.1004179.s019.tif]

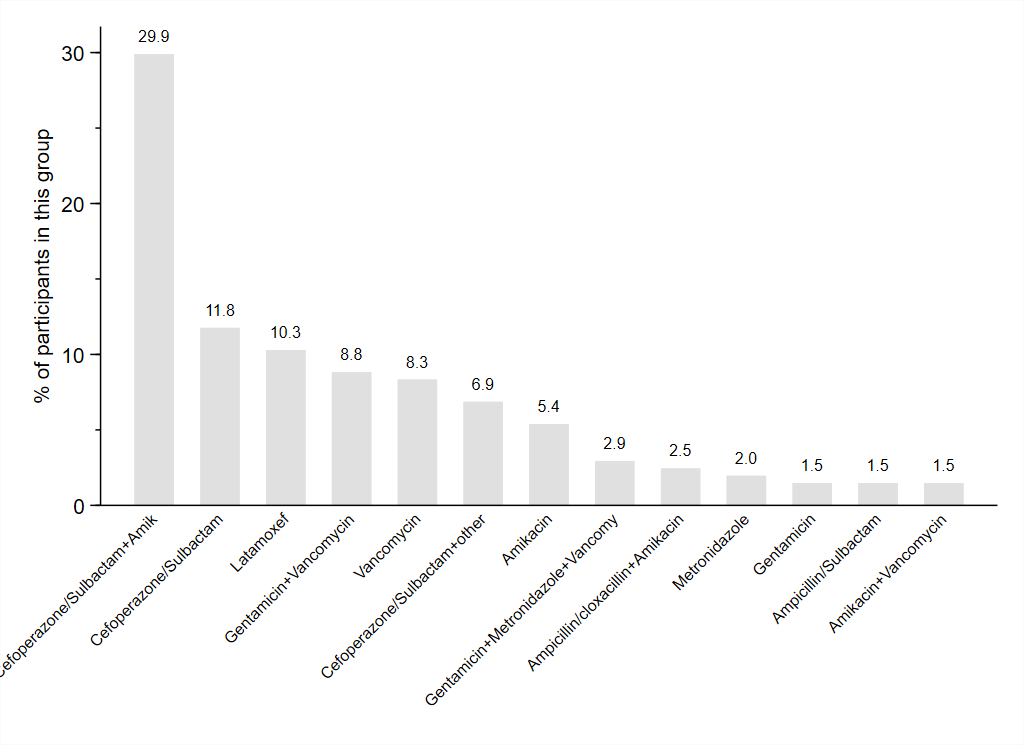

Supplement: S15 Fig — Other antibiotics used as initial regimen. (TIF) [file pmed.1004179.s020.tif]

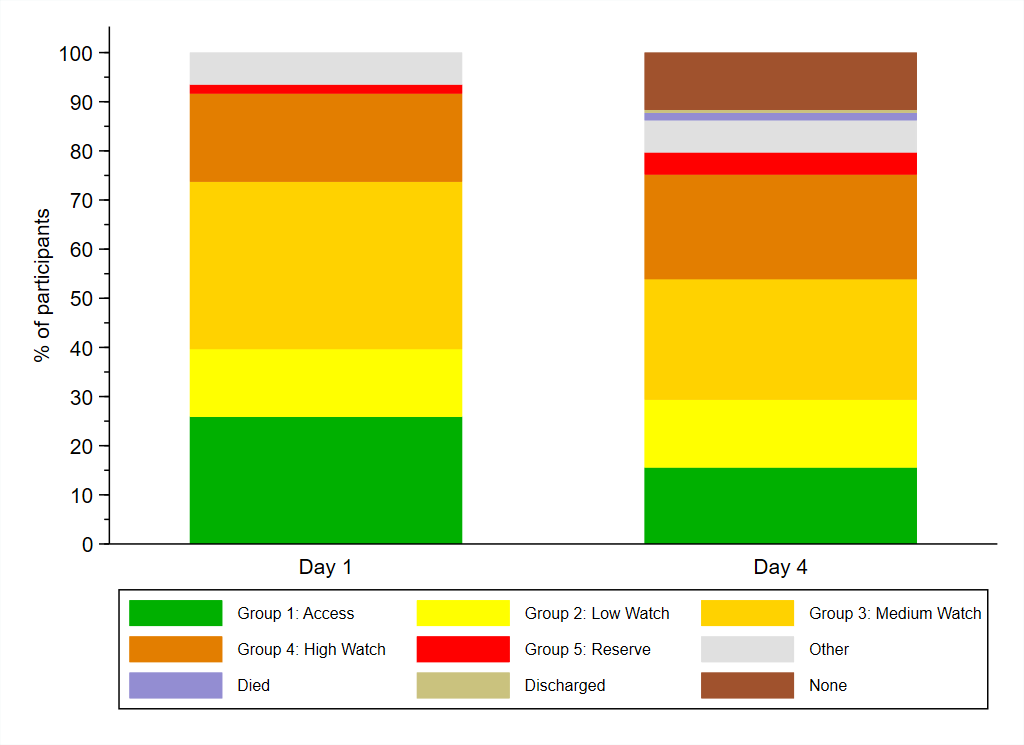

Supplement: S16 Fig — (TIF) [file pmed.1004179.s021.tif]

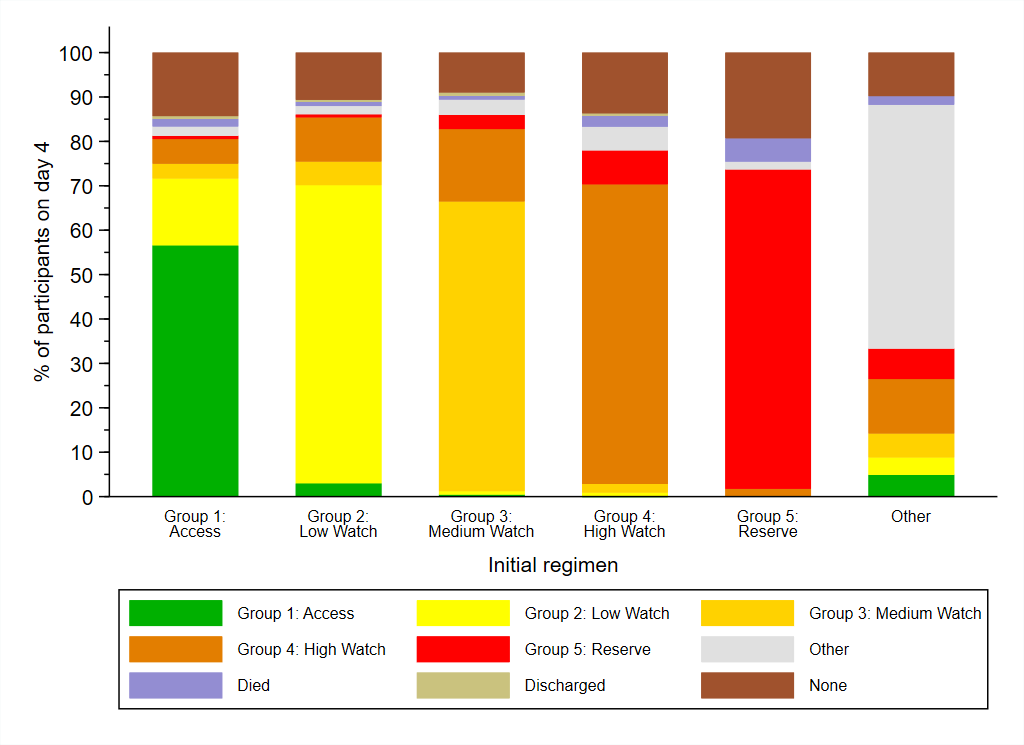

Supplement: S17 Fig — (TIF) [file pmed.1004179.s022.tif]

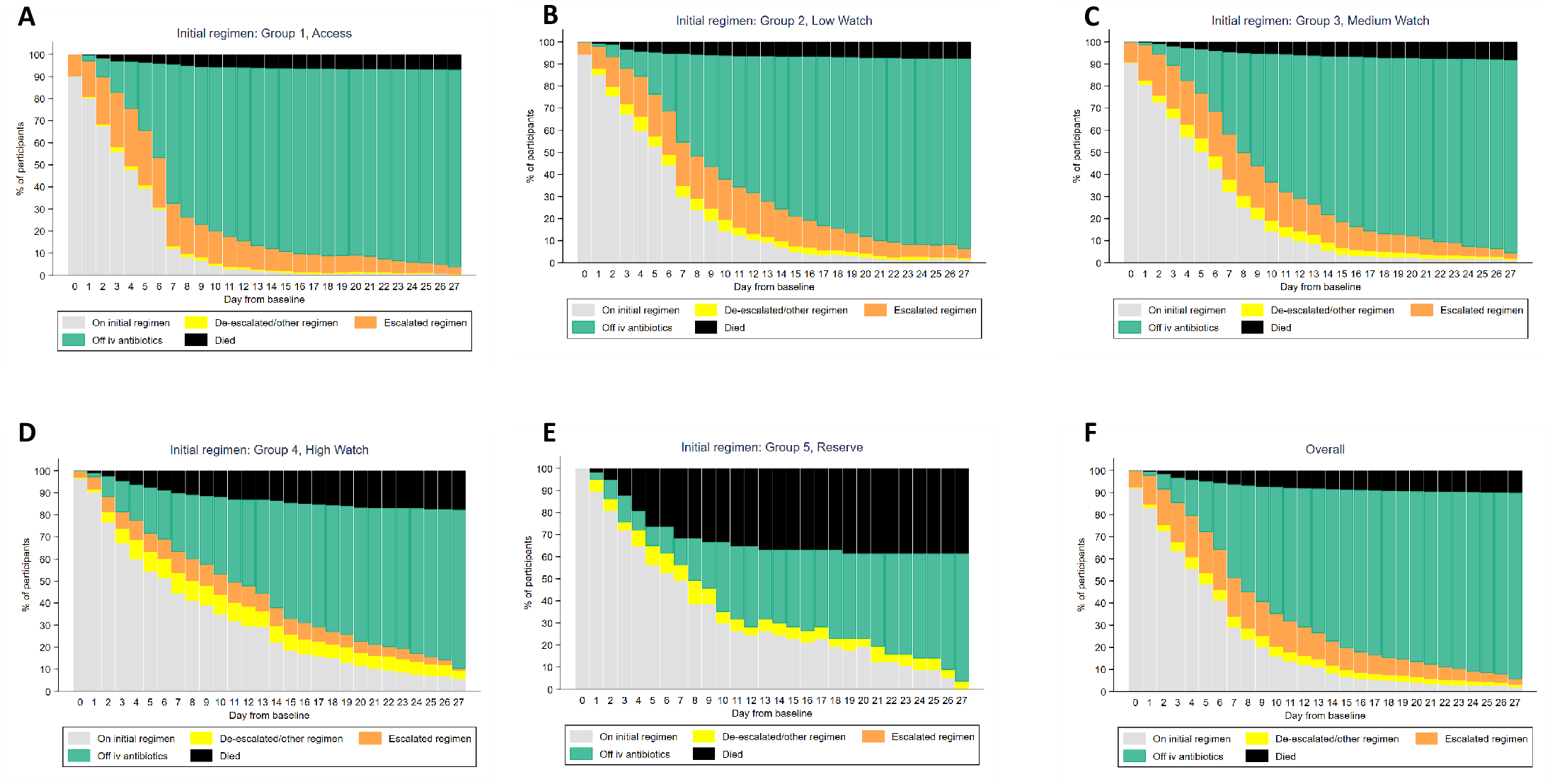

Supplement: S18 Fig — Cross-sectional analysis. Ignoring (unknown) treatment after transfer/readmission to another hospital. (TIF) [file pmed.1004179.s023.tif]

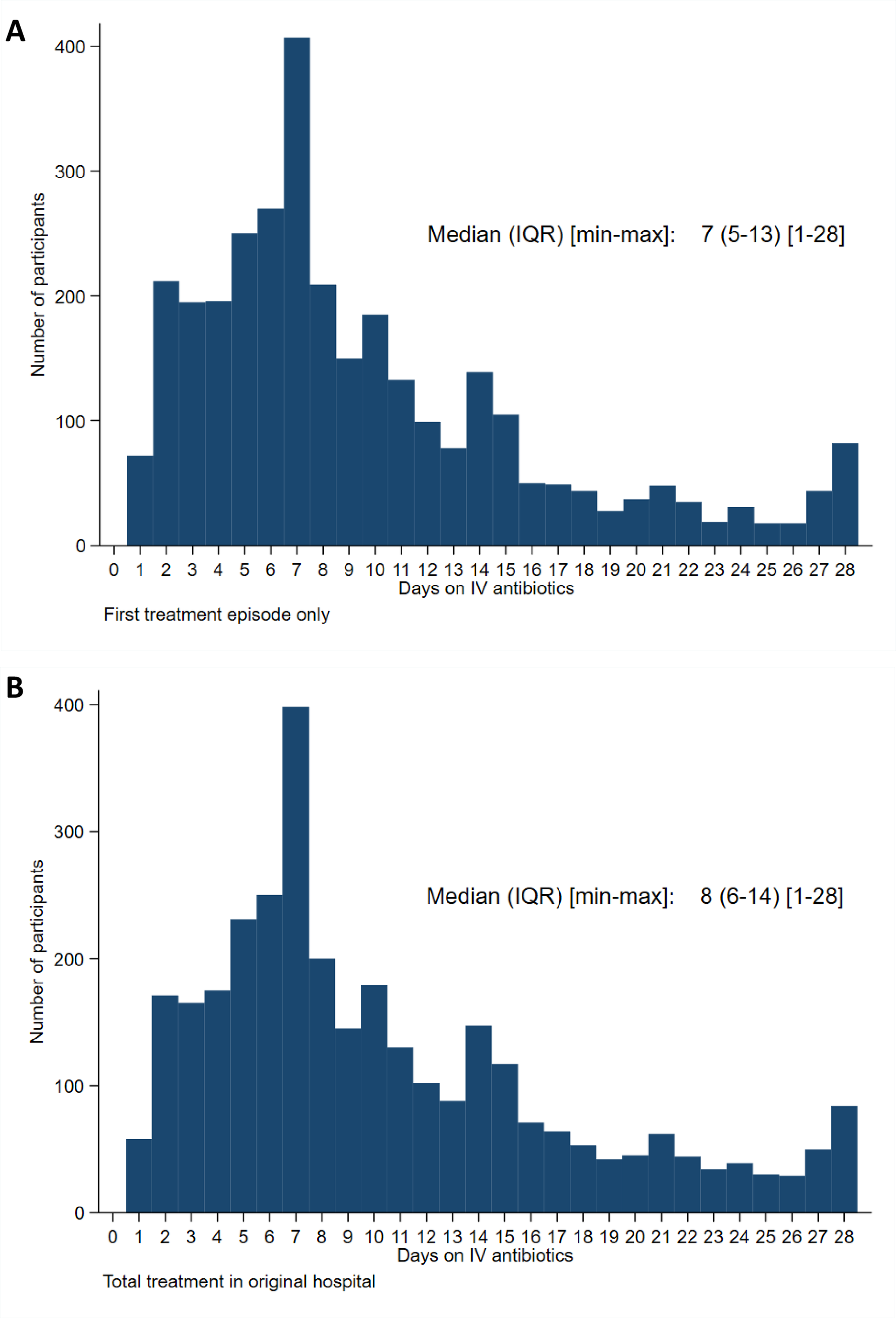

Supplement: S19 Fig — Peak at days 27/28 due to infants still on IV antibiotics at the end of follow-up. (TIF) [file pmed.1004179.s024.tif]

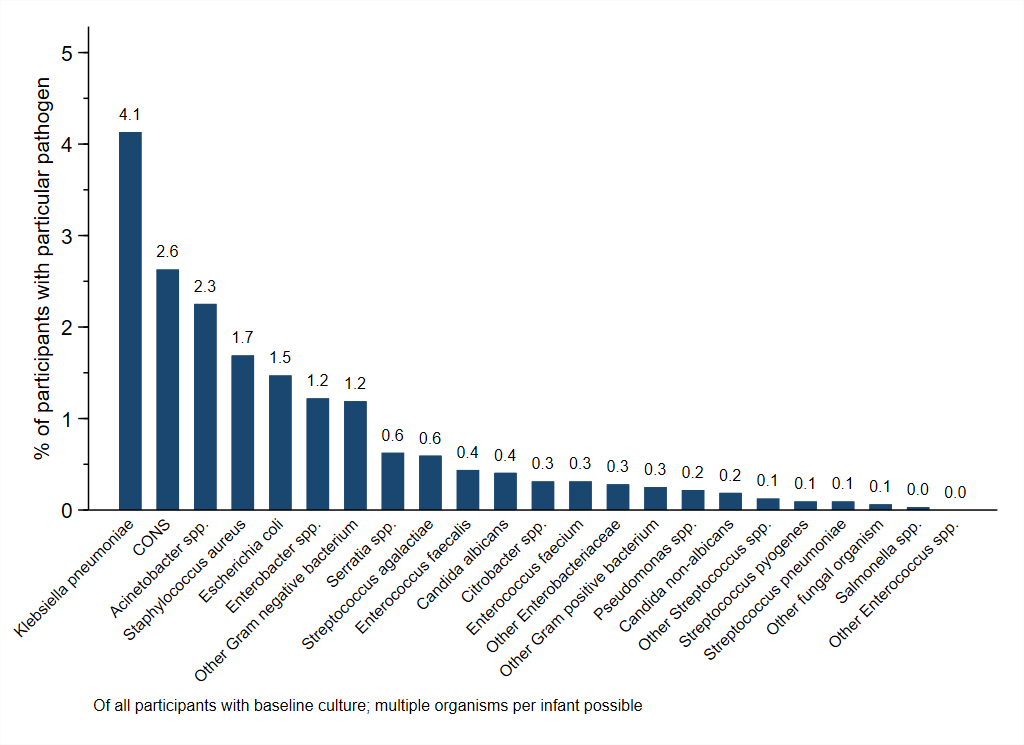

Supplement: S20 Fig — Prevalence of pathogens in participants with a baseline blood culture. (TIF) [file pmed.1004179.s025.tif]

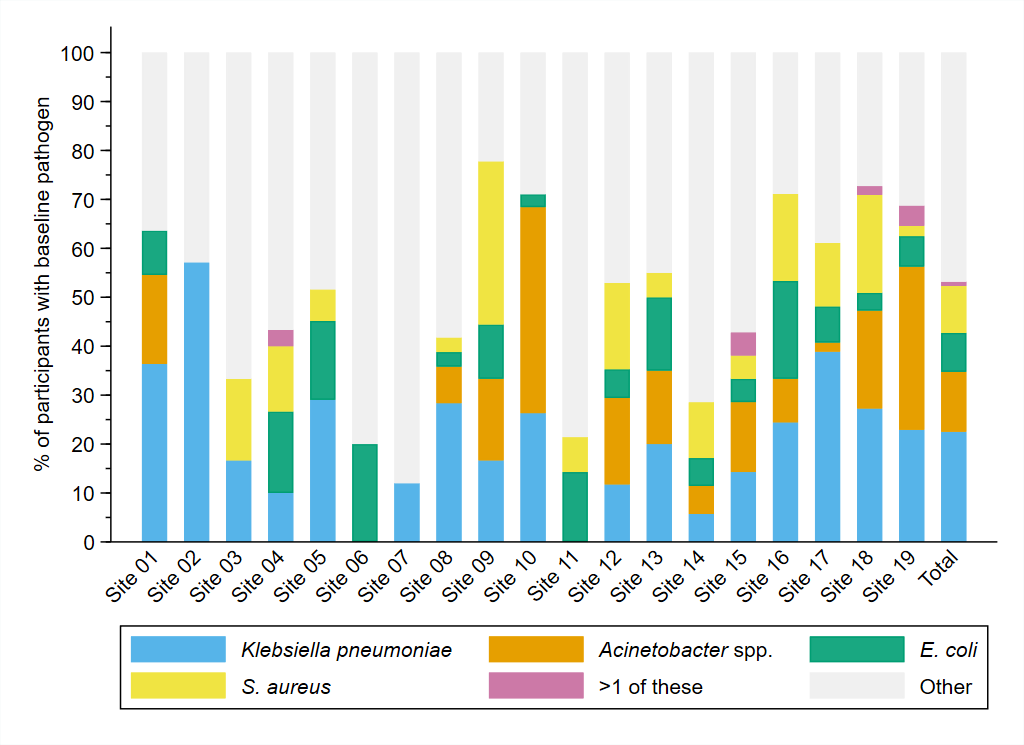

Supplement: S21 Fig — (TIF) [file pmed.1004179.s026.tif]

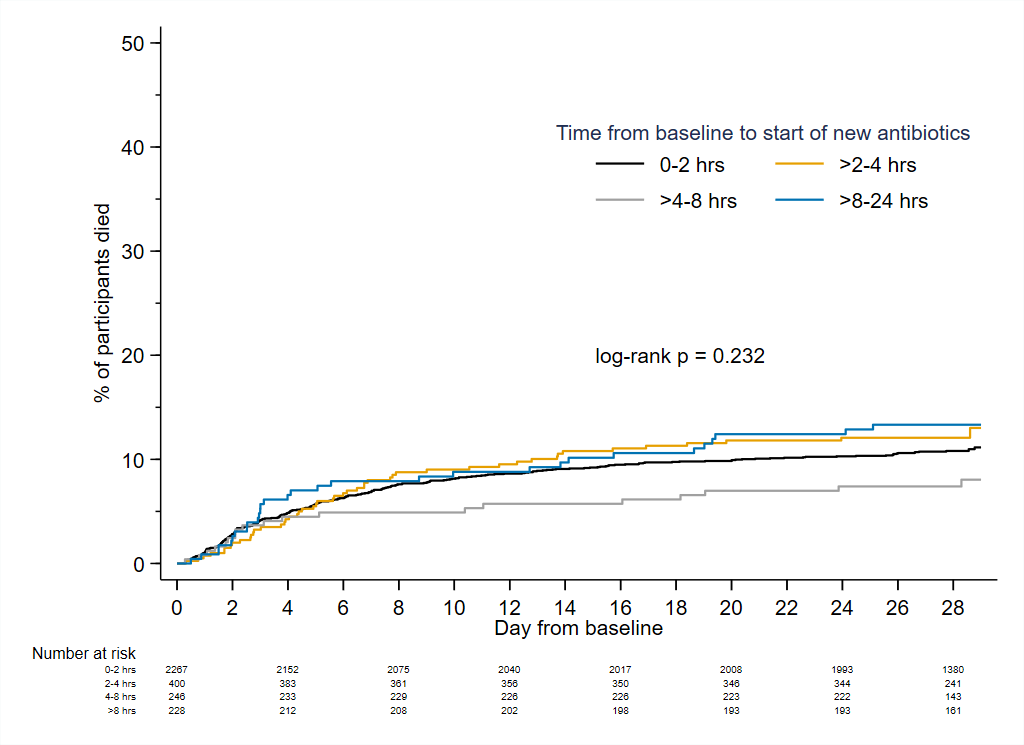

Supplement: S22 Fig — P-value derived from log-rank test. (TIF) [file pmed.1004179.s027.tif]

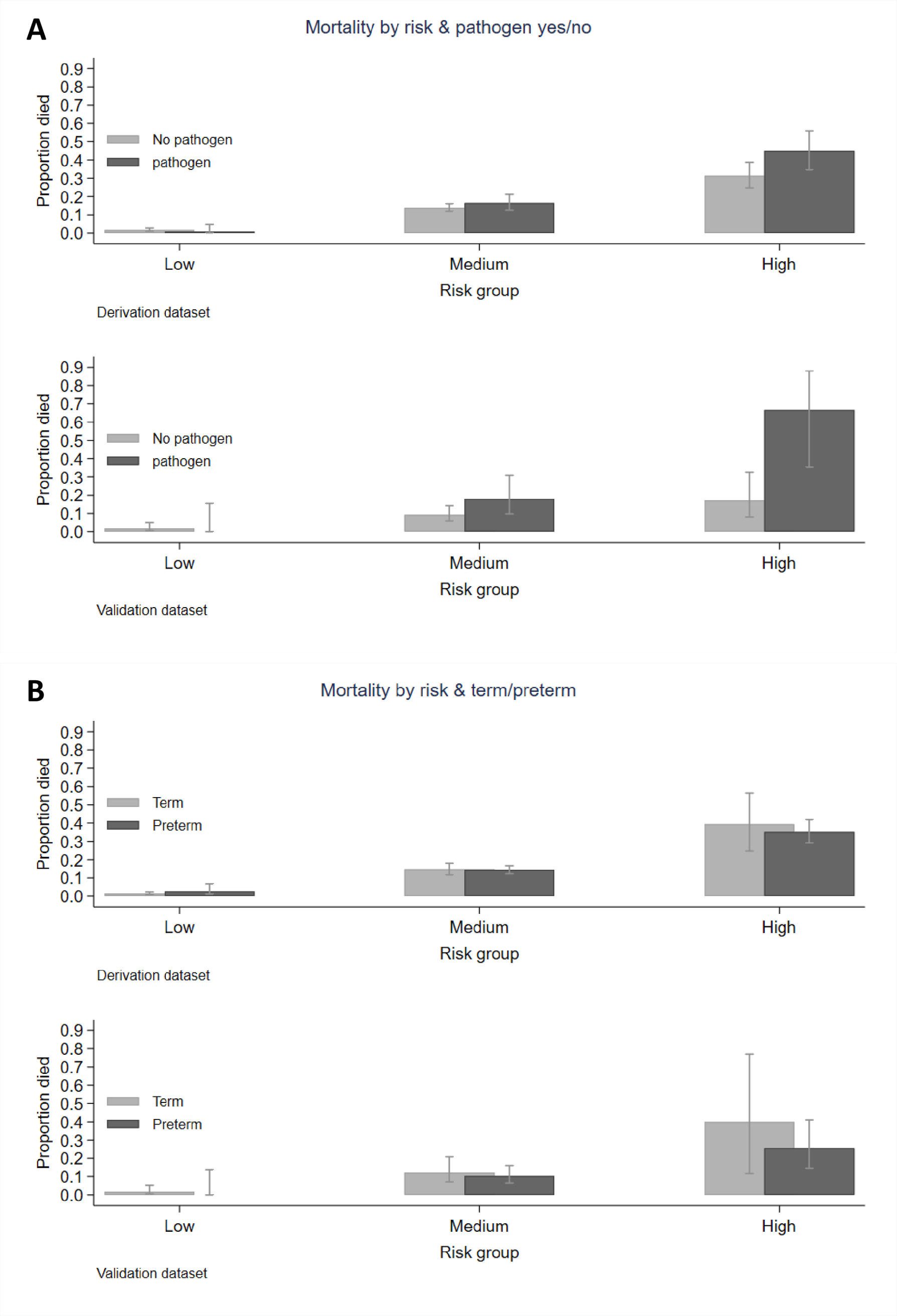

Supplement: S23 Fig — (A) Mortality (95% CI) in risk groups based on the Severity Score and pathogen/no pathogen at baseline. (B) Mortality (95% CI) in risk groups based on the Severity Score and preterm/term birth. (TIF) [file pmed.1004179.s028.tif]

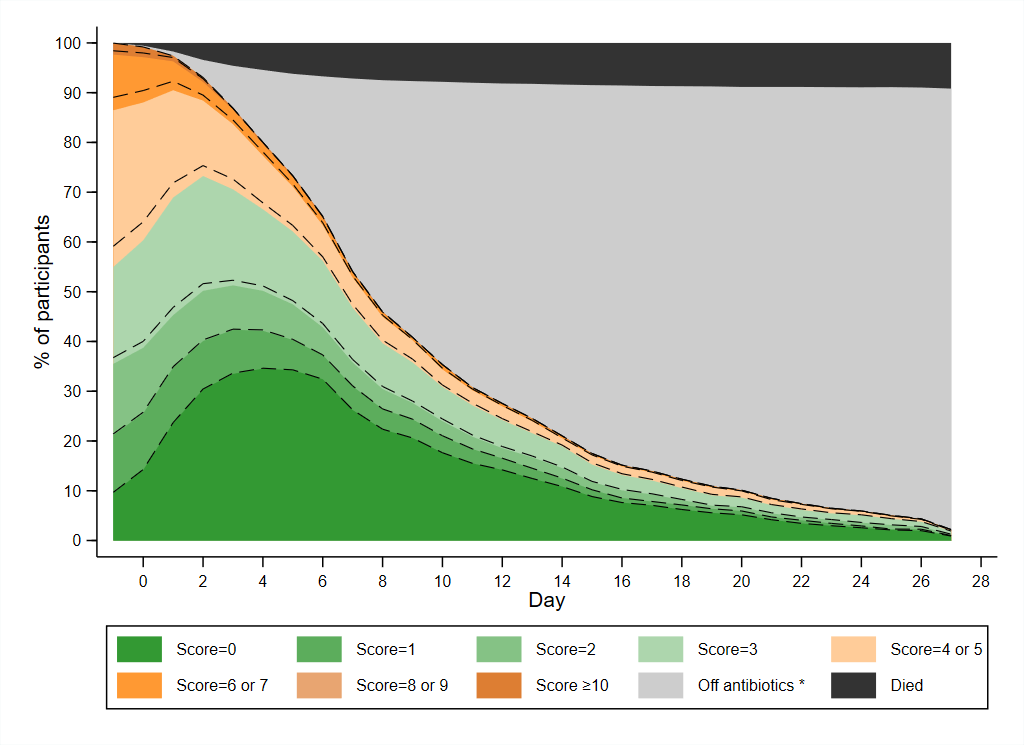

Supplement: S25 Fig — Dashed line: for each score point, indicates those babies who eventually die on antibiotics; * Restart of antibiotics ignored. (TIF) [file pmed.1004179.s030.tif]

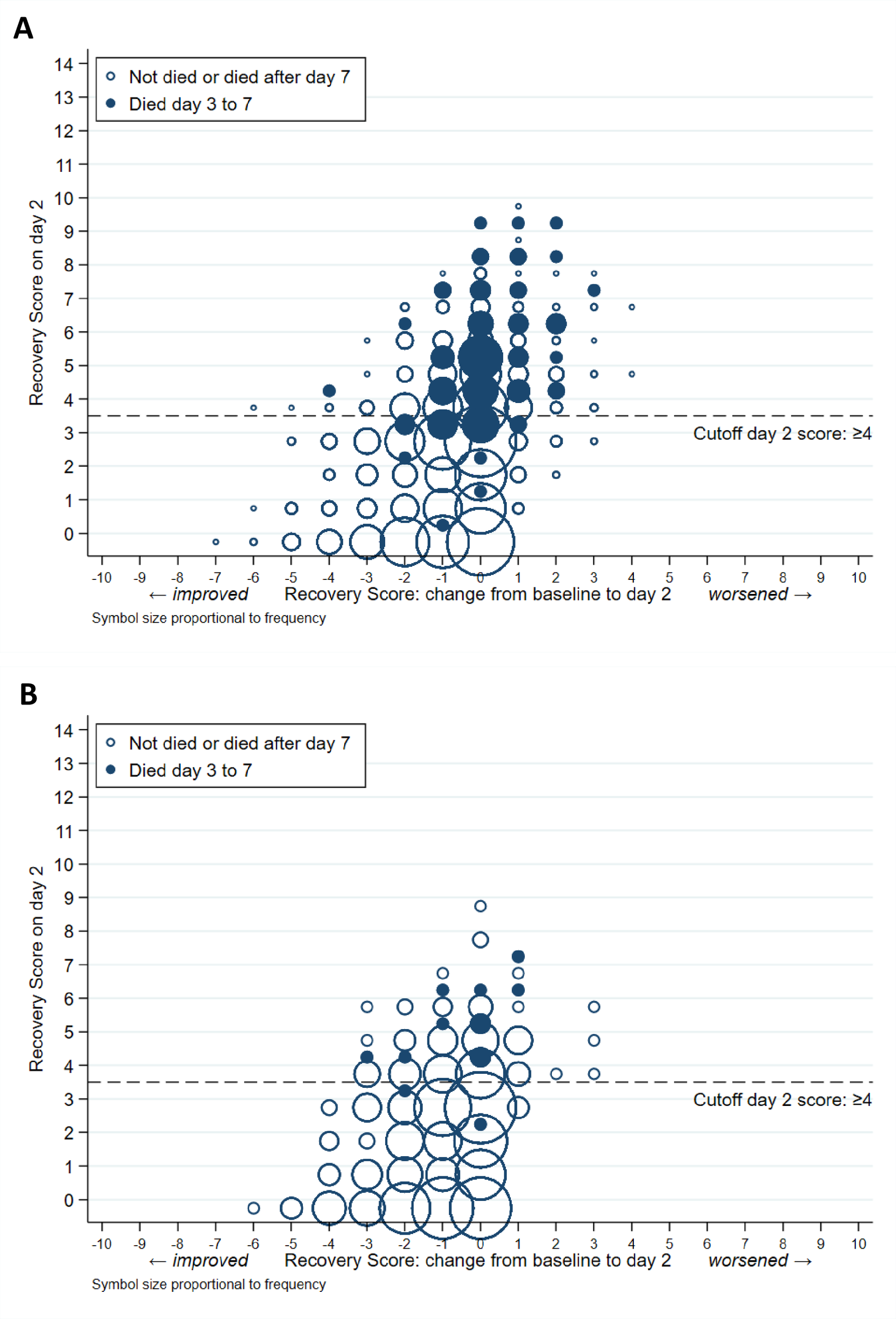

Supplement: S26 Fig — (A) Derivation data. (B) Validation data. (TIF) [file pmed.1004179.s031.tif]
