# Supplementary material for: Patterns of antibiotic use, pathogens, and prediction of mortality in hospitalized neonates and young infants with sepsis: A global neonatal sepsis observational cohort study (NeoOBS)
Source: PLoS Med. 2023 Jun 8;20(6):e1004179. doi: 10.1371/journal.pmed.1004179 (PMC10249878; doi:10.1371/journal.pmed.1004179)
Supplement: S24 Fig — Score based on WHO signs of Clinical Severe Infection, compared with a modified NeoSep Severity Score excluding unmodifiable infant/birth characteristics. pSBI = Possible serious bacterial infection. (PDF) [file pmed.1004179.s029.pdf]

S24 Fig. Comparison between WHO pSBI signs and NeoSep Severity Score for predicting 28-day mortality.

<table>
<tr><th colspan="2">Score based on WHO signs of Clinical Severe Infection</th><th colspan="2">Modified NeoSep Severity Score</th></tr>
<tr><th>Derivation data</th><th>Validation data</th><th>Derivation data</th><th>Validation data</th></tr>
<tr>
<td>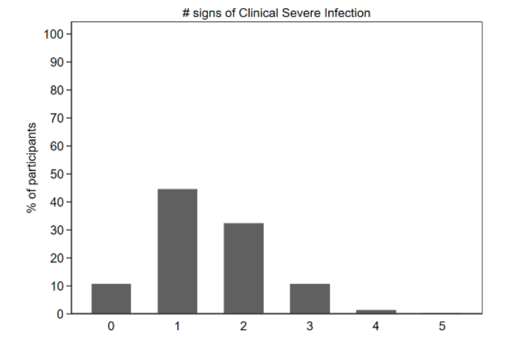
</td>
<td>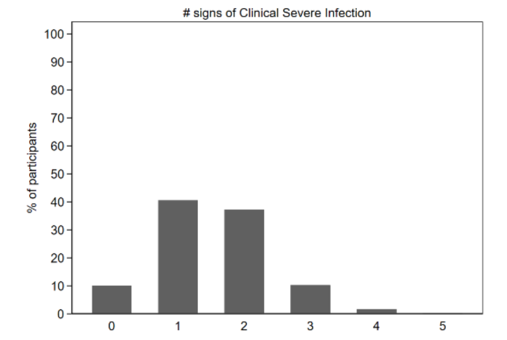
</td>
<td>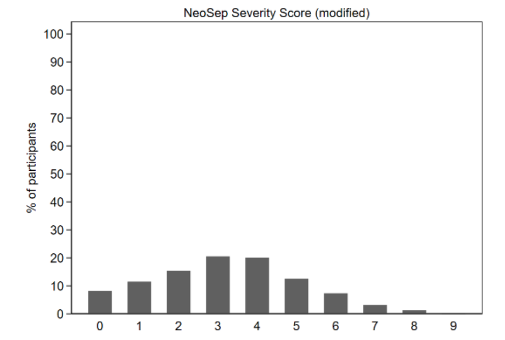
</td>
<td>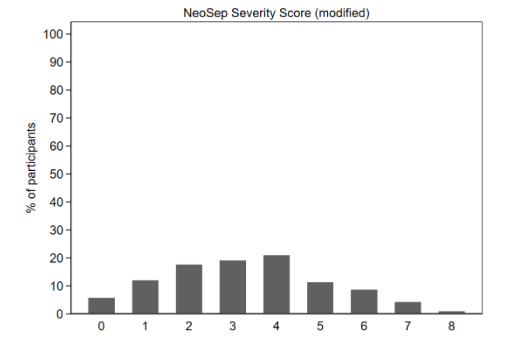
</td>
</tr>
<tr>
<td>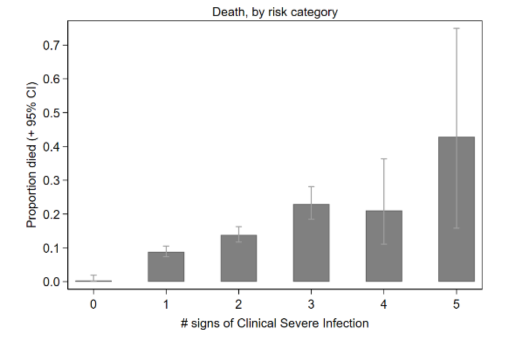
</td>
<td>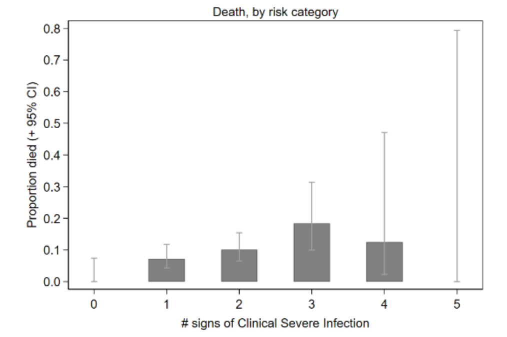
</td>
<td>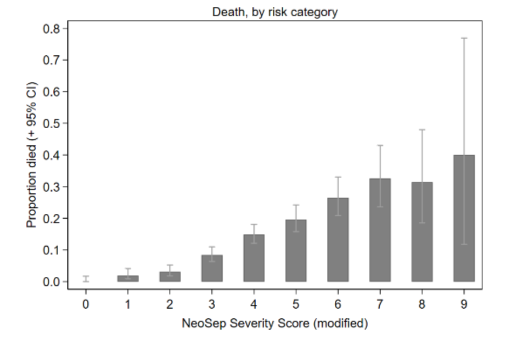
</td>
<td>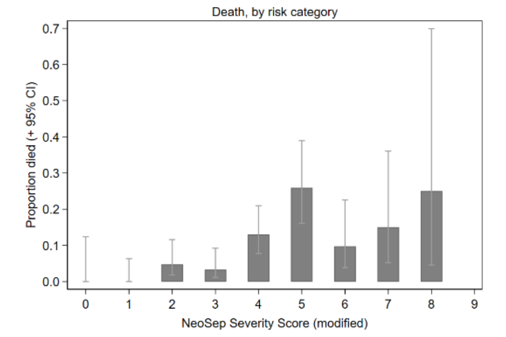
</td>
</tr>
<tr><td>Harrell C: 0.649 (0.621-0.675)</td><td>Harrell C: 0.629 (95% CI 0.559-0.699)</td><td>Harrell C: 0.741 (0.717-0.765)</td><td>Harrell C: 0.716 (0.651-0.774)</td></tr>
</table>
