# Supplementary material for: Patterns of antibiotic use, pathogens, and prediction of mortality in hospitalized neonates and young infants with sepsis: A global neonatal sepsis observational cohort study (NeoOBS)
Source: PLoS Med. 2023 Jun 8;20(6):e1004179. doi: 10.1371/journal.pmed.1004179 (PMC10249878; doi:10.1371/journal.pmed.1004179)
Supplement: S1 Table — * Severe chest wall in-drawing, increased requirement for oxygen or respiratory support; ͳ Observed or reported, including feeding intolerance. (PDF) [file pmed.1004179.s032.pdf]

S1 Table. Candidate factors for the development of the NeoObs Severity Score.

**Infant characteristics**	**Age at baseline**	
	Sex	Male
		Female
	Birth weight	
	Gestational age at birth	
**Birth history**	Birth status	Hospitalized since birth
		Admitted from home/community
	Time from admission to enrolment	
	Congenital anomalies	
**Supportive Care at baseline**	IV fluid	
	Parenteral nutrition (TPN)	
	Maximum respiratory support	None
		Oxygen supplementation
		Non-invasive ventilation
		Invasive ventilation
	Nasogastric tube	
**Vital parameters**	Oxygen saturation	
	Respiratory rate	
	Heart rate	
	Temperature	
**Clinical signs at baseline (≥5%)**	Respiratory signs*	
	Difficulty feeding [Ŧ]	
	Lethargy/reduced movement	Neither
		Lethargy only
		Reduced/no movement
	Abdominal distension	
	Evidence of shock	
	Apnoea	
	Jaundice requiring phototherapy	
	Grunting	
	Hypotonia/floppiness	
	Cyanosis	
	Irritability	
	Vomiting	
	Convulsions	
