# Supplementary material for: Patterns of antibiotic use, pathogens, and prediction of mortality in hospitalized neonates and young infants with sepsis: A global neonatal sepsis observational cohort study (NeoOBS)
Source: PLoS Med. 2023 Jun 8;20(6):e1004179. doi: 10.1371/journal.pmed.1004179 (PMC10249878; doi:10.1371/journal.pmed.1004179)
Supplement: S2 Table — (PDF) [file pmed.1004179.s033.pdf]

S2 Table. Numbers of births and admissions in each site (per 6 months).

Site Number	Live Births per 6 months	Neonatal admissions per 6 months
Site 1	0	821
Site 2	410	193
Site 3	857	274
Site 4	0	1372
Site 5	0	1850
Site 6	7146	816
Site 7	506	167
Site 8	8758	1617
Site 9	6277	1628
Site 10	3275	669
Site 11	0	422
Site 12	3063	731
Site 13	358	432
Site 14	2678	1303
Site 15	0	690
Site 16	0	2031
Site 17	4036	1819
Site 18	4338	838
Site 19	1346	634
