# Supplementary material for: Patterns of antibiotic use, pathogens, and prediction of mortality in hospitalized neonates and young infants with sepsis: A global neonatal sepsis observational cohort study (NeoOBS)
Source: PLoS Med. 2023 Jun 8;20(6):e1004179. doi: 10.1371/journal.pmed.1004179 (PMC10249878; doi:10.1371/journal.pmed.1004179)
Supplement: S3 Table — Proportion of infants with congenital anomalies at enrolment. (PDF) [file pmed.1004179.s034.pdf]

S3 Table. Congenital anomalies.

**Congenital anomalies**	**N=3204**
Any congenital anomalies	265 (8.3%)
of which >1 congenital anomaly	44 (16.6%)
Respiratory	14 (0.4%)
Genito-urinary	37 (1.2%)
Heart	84 (2.6%)
Gastrointestinal system	80 (2.5%)
Brain and spinal cord	25 (0.8%)
Musculoskeletal	27 (0.8%)
Cleft lip +/- palate	12 (0.4%)
Congenital diaphragmatic hernia	8 (0.2%)
Cranio-facial	9 (0.3%)
Genetic syndrome	12 (0.4%)
Other	15 (0.5%)
