# Supplementary material for: Patterns of antibiotic use, pathogens, and prediction of mortality in hospitalized neonates and young infants with sepsis: A global neonatal sepsis observational cohort study (NeoOBS)
Source: PLoS Med. 2023 Jun 8;20(6):e1004179. doi: 10.1371/journal.pmed.1004179 (PMC10249878; doi:10.1371/journal.pmed.1004179)
Supplement: S4 Table — IQR = interquartile range, CRP = C-reactive protein. (PDF) [file pmed.1004179.s035.pdf]

S4 Table. Laboratory results from blood at baseline.

	N=3204
White blood cell count analysed	2800 (87.4%)
White blood cells (10^9/L), median (IQR)	12.4 (7.9, 19.4)
Abnormal white blood cells (<4 or >20 10^9 cells/L)	875/2800 (31.3%)
Neutrophils analysed	2368 (73.9%)
Neutrophils (10^9/L), median (IQR)	6.8 (3.3, 12.4)
Neutropenia (<1.5 10^9 cells/L)	235/2368 (9.9%)
Platelets analysed	2776 (86.6%)
Platelets (10^9/L), median (IQR)	241.0 (158.4, 335.0)
Thrombocytopenia (<150 10^9/L)	619/2776 (22.3%)
Haemoglobin analysed	2802 (87.5%)
Haemoglobin (g/dL), median (IQR)	14.6 (12.3, 16.7)
Immature-to-total polymorph ratio analysed	89 (2.8%)
Abnormal Immature-to-total polymorph ratio	17/89 (19%)
CRP analysed	2286 (71.3%)
CRP (mg/L), median (IQR)	15 (4, 43)
Abnormal CRP (>10mg/L)	1306/2286 (57.1%)
Base excess analysed	1492 (46.6%)
Base excess	352/1492 (23.6%)
Lactate analysed	1283 (40.0%)
Abnormal lactate	1034/1283 (80.6%)
Acidosis analysed	1611 (50.3%)
Acidosis	1144/1611 (71.0%)
Glucose analysed	1816 (56.7%)
Glucose (mmol/L), median (IQR)	4.9 (3.7, 6.2)
Bilirubin analysed	1187 (37.0%)
Bilirubin (µmol/L), median (IQR)	123 (63, 184)
