# Supplementary material for: Patterns of antibiotic use, pathogens, and prediction of mortality in hospitalized neonates and young infants with sepsis: A global neonatal sepsis observational cohort study (NeoOBS)
Source: PLoS Med. 2023 Jun 8;20(6):e1004179. doi: 10.1371/journal.pmed.1004179 (PMC10249878; doi:10.1371/journal.pmed.1004179)
Supplement: S5 Table — Most common initial regimens in infants who started new IV antibiotics within 24 h from baseline blood culture. (PDF) [file pmed.1004179.s036.pdf]

S5 Table. Most common initial antibiotic regimens.

Initial regimen	N=3141
Ampicillin + gentamicin	403 (12.8%)
Piperacillin/tazobactam + amikacin	356 (11.3%)
Ceftazidime	297 (9.5%)
Meropenem + vancomycin	246 (7.8%)
Meropenem	201 (6.4%)
Ceftazidime + amikacin	139 (4.4%)
Cefotaxime	92 (2.9%)
Ampicillin + amikacin	87 (2.8%)
Cefotaxime + ampicillin	78 (2.5%)
Benzylpenicillin (penicillin G) + gentamicin	61 (1.9%)
Cefoperazone/sulbactam+amikacin	61 (1.9%)
Amoxicillin/clavulanic acid + amikacin	59 (1.9%)
Ciprofloxacin + amikacin	58 (1.9%)
Ceftriaxone	58 (1.9%)
Colistin (± other drug)	57 (1.8%)
Amoxicillin/clavulanic acid	55 (1.8%)
Ceftazidime + benzylpenicillin (penicillin G)	55 (1.8%)
Piperacillin/tazobactam	54 (1.7%)
Cefotaxime + amikacin	50 (1.6%)
Meropenem + amikacin	49 (1.6%)
Ampicillin + tobramycin	37 (1.2%)
Other	588 (18.7%)
