# Supplementary material for: Patterns of antibiotic use, pathogens, and prediction of mortality in hospitalized neonates and young infants with sepsis: A global neonatal sepsis observational cohort study (NeoOBS)
Source: PLoS Med. 2023 Jun 8;20(6):e1004179. doi: 10.1371/journal.pmed.1004179 (PMC10249878; doi:10.1371/journal.pmed.1004179)
Supplement: S6 Table — HAI = healthcare-associated infection (occurring ≥48 h after admission). (PDF) [file pmed.1004179.s037.pdf]

S6 Table. Most common initial antibiotic regimens, by time from admission.

a) **Initial regimens for sepsis within 48hrs of admission (non-HAI)**	N=1861	b) **Initial regimen for sepsis >48hrs after admission (HAI)**	N=1280
Ampicillin + Gentamicin	366 (19.7%)	Meropenem + Vancomycin	224 (17.5%)
Ceftazidime	248 (13.3%)	Piperacillin/Tazobactam + Amikacin	197 (15.4%)
Piperacillin/Tazobactam + Amikacin	159 (8.5%)	Meropenem	137 (10.7%)
Ceftazidime + Amikacin	102 (5.5%)	Colistin (± other drug)	55 (4.3%)
Ampicillin + Amikacin	86 (4.6%)	Cefoperazone/Sulbactam+Amikacin	50 (3.9%)
Cefotaxime	73 (3.9%)	Ceftazidime	49 (3.8%)
Cefotaxime + Ampicillin	69 (3.7%)	Ceftazidime + Amikacin	37 (2.9%)
Meropenem	64 (3.4%)	Ampicillin + Gentamicin	37 (2.9%)
Benzylpenicillin (Penicillin G) + Gentamicin	57 (3.1%)	Meropenem + Amikacin	34 (2.7%)
Amoxicillin/Clavulanic acid	44 (2.4%)	Piperacillin/Tazobactam	27 (2.1%)
Ceftriaxone	44 (2.4%)	Amoxicillin/Clavulanic acid + Amik.	20 (1.6%)
Ceftazidime + Benzylpenicillin (Penicillin G)	44 (2.4%)	Cefotaxime	19 (1.5%)
Ciprofloxacin + Amikacin	40 (2.2%)	Ciprofloxacin + Amikacin	18 (1.4%)
Amoxicillin/Clavulanic acid + Amikacin	39 (2.1%)	Ampicillin + Netilmicin	18 (1.4%)
Cefotaxime + Amikacin	37 (2.0%)	Gentamicin+Vancomycin	17 (1.3%)
Ampicillin + Tobramycin	30 (1.6%)	Vancomycin	16 (1.3%)
