# Supplementary material for: Patterns of antibiotic use, pathogens, and prediction of mortality in hospitalized neonates and young infants with sepsis: A global neonatal sepsis observational cohort study (NeoOBS)
Source: PLoS Med. 2023 Jun 8;20(6):e1004179. doi: 10.1371/journal.pmed.1004179 (PMC10249878; doi:10.1371/journal.pmed.1004179)
Supplement: S7 Table — Note: OR = odds ratio, derived from logistic regression models adjusted for center. Time in hospital was analyzed as continuous variable using fractional polynomials with powers 1 1. For illustrative reasons, in this table we report odds ratios for specific values. (PDF) [file pmed.1004179.s038.pdf]

S7 Table. Factors associated with Non-WHO recommended Regimens (Groups 3-5).

		**Univariable models**		**Multivariable model**	
**Factors at presentation**		**OR (95% CI)**	**p-value**	**OR (95% CI)**	**p-value**
Birth weight	Per additional kg	0.39 (0.34-0.44)	<0.001	0.57 (0.47-0.69)	<0.001
Time in hospital	0 hours<br>24 hours<br>48 hours<br>1 week	reference<br>2.66 (2.35-3.01)<br>5.75 (4.63-7.13)<br>39.98 (26.46-60.42)	<0.001	reference<br>2.28 (1.97-2.65)<br>4.41 (3.41-5.69)<br>24.7 (15.2-40.3)	<0.001
Central line / catheter		7.94 (4.94-12.76)	<0.001	3.48 (1.74-6.94)	<0.001
On iv antibiotics at baseline		12.30 (8.66-17.45)	<0.001	5.71 (3.73-8.77)	<0.001
Previously positive culture		10.46 (2.98-36.81)	<0.001	25.71 (3.00-220.7)	0.003
Previous surgery		15.88 (5.97-42.29)	<0.001	5.18 (1.65-16.28)	0.005
Sepsis score	Per additional score point	1.40 (1.31-1.50)	<0.001	1.27 (1.16-1.40)	<0.001
