# Supplementary material for: Patterns of antibiotic use, pathogens, and prediction of mortality in hospitalized neonates and young infants with sepsis: A global neonatal sepsis observational cohort study (NeoOBS)
Source: PLoS Med. 2023 Jun 8;20(6):e1004179. doi: 10.1371/journal.pmed.1004179 (PMC10249878; doi:10.1371/journal.pmed.1004179)
Supplement: S8 Table — Note: * Organisms isolated in only 1 infant per group. (PDF) [file pmed.1004179.s039.pdf]

S8 Table. Organisms isolated from blood at baseline, by time from admission.

**Organisms isolated within 48hrs of admission (non-HAI)**	N=1883	**Organisms isolated >48hrs after admission (HAI)**	N=1312
Escherichia coli	32 (1.7%)	*Klebsiella pneumoniae*	114 (8.7%)
Acinetobacter spp.	21 (1.1%)	*Coagulase-negative Staphylococci*	70 (5.3%)
Klebsiella pneumoniae	18 (1.0%)	*Acinetobacter* spp.	51 (3.9%)
Staphylococcus aureus	17 (0.9%)	*Staphylococcus aureus*	37 (2.8%)
Coagulase-negative staphylococci	14 (0.7%)	*Enterobacter* spp.	32 (2.4%)
Streptococcus agalactiae	13 (0.7%)	*Serratia* spp.	19 (1.5%)
Enterobacter spp.	7 (0.4%)	*Elizabethkingia meningoseptica*	15 (1.1%)
Burkholderia spp.	6 (0.3%)	*Escherichia coli*	15 (1.1%)
Enterococcus faecalis	3 (0.2%)	*Candida albicans*	13 (1.0%)
Pseudomonas spp.	3 (0.2%)	*Enterococcus faecalis*	11 (0.8%)
Streptococcus pyogenes	3 (0.2%)	*Citrobacter* spp.	8 (0.6%)
Citrobacter spp.	2 (0.1%)	*Enterococcus faecium*	8 (0.6%)
Enterococcus faecium	2 (0.1%)	*Klebsiella oxytoca*	7 (0.5%)
Elizabethkingia anophelis	2 (0.1%)	*Candida* non-*albicans*	6 (0.5%)
Other*	8 (0.4%)	*Streptococcus agalactiae*	6 (0.5%)
		Burkholderia spp.	6 (0.5%)
		Candida non-albicans	6 (0.5%)
		Elizabethkingia anophelis	5 (0.4%)
		Bacillus spp.	4 (0.3%)
		Pseudomonas spp.	4 (0.3%)
		Streptococcus pneumoniae	2 (0.2%)
		Other*	12 (0.9%)
