# Supplementary material for: Patterns of antibiotic use, pathogens, and prediction of mortality in hospitalized neonates and young infants with sepsis: A global neonatal sepsis observational cohort study (NeoOBS)
Source: PLoS Med. 2023 Jun 8;20(6):e1004179. doi: 10.1371/journal.pmed.1004179 (PMC10249878; doi:10.1371/journal.pmed.1004179)
Supplement: S9 Table — Note: S = susceptible; I = intermediate; R = resistant. Denominators across drugs variables due to available susceptibility results. (PDF) [file pmed.1004179.s040.pdf]

S9 Table. Susceptibility result for pathogens in baseline blood culture.

		Klebsiella pneumoniae	*Acinetobacter* spp.	*Escherichia coli*	*Staphylococcus aureus*	*Serratia* spp.	*Burkholderia* spp.	*Elizabethkingia meningoseptica*
**ampicillin**	S	0	0	0	21 (39%	0	0	0
	R	132 (100%)	72 (100%)	47 (100%)	33 (61%)	20 (100%)	12 (100%)	15 (100%)
**gentamicin**	S	50 (38%)	19 (27%)	37 (80%)	24 (57%)	18 (90%)	0	0
	I	5 (4%)	2 (3%)	0	3 (7%)	0	0	0
	R	75 (58%)	50 (70%)	9 (20%)	15 (36%)	2 (10%)	12 (100%)	15 (100%)
**ceftriaxone**	S	29 (23%)	0	27 (63%)	21 (39%)	7 (64%)	0	0
	I	2 (2%)	0	0	0	0	0	0
	R	96 (76%)	72 (100%)	16 (37%)	33 (61%)	4 (36%)	12 (100%)	15 (100%)
**cefotaxime**	S	30 (23%)	0	29 (64%)	21 (39%)	7 (64%)	0	0
	I	2 (2%)	0	0	0	0	0	0
	R	96 (75%)	72 (100%)	16 (36%)	33 (61%)	4 (36%)	12 (100%)	15 (100%)
**ceftazidime**	S	26 (28%)	0	29 (78%)	0	7 (64%)	12 (100%)	0
	I	2 (2%)	0	0	0	0	0	0
	R	66 (70%)	72 (100%)	8 (22%)	54 (100%)	4 (36%)	0	15 (100%)
**ciprofloxacin**	S	51 (50%)	14 (24%)	11 (44%)	21 (53%)	13 (93%)	1 (13%)	4 (31%)
	I	4 (4%)	0	2 (8%)	0	1 (7%)	2 (25%)	9 (69%)
	R	48 (47%)	45 (76%)	12 (48%)	19 (48%)	0	5 (63%)	0
**levofloxacin**	S	20 (56%)	13 (48%)	10 (53%)	16 (64%)	4 (80%)	3 (27%)	13 (100%)
	I	2 (6%)	3 (11%)	3 (16%)	0	1 (20%)	1 (9%)	0
	R	14 (39%)	11 (41%)	6 (32%)	9 (36%)	0	7 (64%)	0
**piperacillin-tazobactam**	S	50 (44%)	0	38 (90%)	21 (39%)	6 (75%)	0	1 (7%)
	I	11 (10%)	0	2 (5%)	0	1 (13%)	0	0
	R	53 (46%)	72 (100%)	2 (5%)	33 (61%)	1 (13%)	12 (100%)	13 (93%)
**meropenem**	S	88 (67%)	19 (27%)	38 (97%)	21 (39%)	18 (90%)	10 (91%)	0
	I	1 (1%)	1 (1%)	0	0	0	1 (9%)	0
	R	43 (33%)	50 (71%)	1 (3%)	33 (61%)	2 (10%)	0	15 (100%)
**colistin**	S	39 (100%)	43 (98%)	4 (100%)	0	0	0	0
	I	0	1 (2%)	0	0	0	0	0
	R	0	0	0	54 (100%)	19 (100%)	12 (100%)	15 (100%)
**amikacin**	S	80 (62%)	19 (27%)	43 (93%)	24 (57%)	15 (75%)	0	0
	I	10 (8%)	1 (1%)	1 (2%)	3 (7%)	1 (5%)	0	0
	R	40 (31%)	51 (72%)	2 (4%)	15 (36%)	4 (20%)	12 (100%)	15 (100%)
**vancomycin**	S	0	0	0	42 (100%)	0	0	0
	R	132 (100%)	72 (100%)	47 (100%)	0	20 (100%)	12 (100%)	15 (100%)
**methicillin**	S	31 (23%)	0	24 (51%)	21 (39%)	1 (5%)	0	0
	I	0	0	1 (2%)	0	0	0	0
	R	101 (77%)	0	22 (47%)	33 (61%)	19 (95%)	0	0
