# Supplementary material for: Patterns of antibiotic use, pathogens, and prediction of mortality in hospitalized neonates and young infants with sepsis: A global neonatal sepsis observational cohort study (NeoOBS)
Source: PLoS Med. 2023 Jun 8;20(6):e1004179. doi: 10.1371/journal.pmed.1004179 (PMC10249878; doi:10.1371/journal.pmed.1004179)
Supplement: S10 Table — Note: n = 73 with positive CSF culture in first 7 days. (PDF) [file pmed.1004179.s041.pdf]

S10 Table. Organisms isolated from CSF in first 7 days from baseline.

Class	Organism	Pathogen	Contaminant	Unclassified
Gram-positive	*Streptococcus agalactiae*	3	0	0
	Coagulase-negative Staphylococci	2	7	1
	Enterococcus faecalis	2	1	0
	Enterococcus faecium	2	0	0
	Bacillus spp.	0	12	1
	Micrococcus spp.	0	1	0
	Streptococcus oralis	0	1	0
	Corynebacterium spp.	0	1	0
Gram-negative	*Acinetobacter baumannii*	11	0	0
	Escherichia coli	9	0	0
	Klebsiella pneumoniae	9	0	0
	Elizabethkingia meningoseptica	4	0	0
	Elizabethkingia anophelis	3	0	0
	Enterobacter spp.	2	0	0
	Burkholderia spp.	1	0	0
	Proteus mirabilis	1	0	0
	Sphingomonas paucimobilis	1	0	0
	Acinetobacter ursingii	0	1	0
