# Supplementary material for: Patterns of antibiotic use, pathogens, and prediction of mortality in hospitalized neonates and young infants with sepsis: A global neonatal sepsis observational cohort study (NeoOBS)
Source: PLoS Med. 2023 Jun 8;20(6):e1004179. doi: 10.1371/journal.pmed.1004179 (PMC10249878; doi:10.1371/journal.pmed.1004179)
Supplement: S11 Table — Note: Results are hazard ratio (95% confidence interval). All models are adjusted for site (random effect). * Adjusted for birth weight, gestational age, time in hospital, congenital anomalies, and site; ** adjusted for all factors in NeoSep Severity Score (as in * plus abdominal distension, difficulty in feeding, evidence of shock, lethargy/no movement, temperature, and level of respiratory support); *** including Coagulase-negative staphylococcus. ref. = reference category. (PDF) [file pmed.1004179.s042.pdf]

S11 Table. Pathogens and mortality.

**Factor**	**Univariable**	**Adjusted for unmodifiable baseline predictors***	**Adjusted for all baseline predictors****
Pathogen at baseline	1.65 (1.30 – 2.10)<br>*p<0.001*	1.54 (1.21 – 1.97)<br>*p=0.001*	1.57 (1.22 – 2.02)<br>*p<0.001*
Pathogen type at baseline			
No pathogen	ref.	ref.	ref.
Gram positive pathogen	0.96 (0.57 – 1.62)	1.06 (0.63 – 1.80)	1.35 (0.79 – 2.30)
Gram negative pathogen	1.80 (1.37 – 2.35)	1.62 (1.24 – 2.12)	1.55 (1.17 – 2.04)
Fungal pathogen	4.55 (2.21 – 9.36)<br>*p<0.0001*	3.84 (1.79 – 8.20)<br>*p=0.0003*	4.24 (1.94 – 9.30)<br>*p=0.0007*
Pathogen at baseline			
No pathogen	ref.	ref.	ref.
Acinetobacter spp.	2.70 (1.76 – 4.14)	2.01 (1.30 – 3.10)	1.96 (1.27 – 3.05)
Klebsiella pneumoniae	1.92 (1.29 – 2.86)	1.77 (1.18 – 2.66)	1.49 (0.99 – 2.25)
E. coli	1.77 (0.79 – 4.00)	2.13 (0.94 – 4.81)	2.33 (1.01 – 5.37)
S. aureus	1.03 (0.46 – 2.33)	1.02 (0.45 – 2.31)	1.22 (0.53 – 2.80)
Streptococcus agalactiae	2.65 (0.98 – 7.18)	3.30 (1.20 – 9.03)	3.40 (1.22 – 9.48)
Other pathogen***	1.17 (0.80 – 1.73)<br>*p=0.0001*	1.14 (0.77 – 1.69)<br>*p=0.0032*	1.28 (0.86 – 1.92)<br>*p=0.0098*
