# Supplementary material for: Patterns of antibiotic use, pathogens, and prediction of mortality in hospitalized neonates and young infants with sepsis: A global neonatal sepsis observational cohort study (NeoOBS)
Source: PLoS Med. 2023 Jun 8;20(6):e1004179. doi: 10.1371/journal.pmed.1004179 (PMC10249878; doi:10.1371/journal.pmed.1004179)
Supplement: S12 Table — 1 Outcome for developing NeoSep Severity Score; 2 outcome for developing NeoSep Recovery Score. (PDF) [file pmed.1004179.s043.pdf]

**S12 Table. Characteristics of participants in derivation and validation samples.**

| | **Derivation sample** N=2726 | **Validation sample** N=478 |
|---|---|---|
| WHO region of enrolment | | |
| Africa | 849 (31.1%) | 149 (31.2%) |
| Americas | 68 (2.5%) | 11 (2.3%) |
| South-East Asia | 1021 (37.5%) | 180 (37.7%) |
| Europe | 103 (3.8%) | 18 (3.8%) |
| Western Pacific | 685 (25.1%) | 120 (25.1%) |
| Age at baseline (days), median (IQR) | 5 (1, 15) | 4 (1, 14) |
| Birth weight (grams), median (IQR) | 2497.5 (1400, 3187.5) | 2500 (1400, 3200) |
| Gestational age at birth (weeks), median (IQR) | 37 (31, 39) | 37 (31, 39) |
| Time from admission to enrolment (hours), median (IQR) | 21 (1, 128) | 27 (1, 117) |
| Congenital anomalies | 228 (8.4%) | 37 (7.7%) |
| Sepsis group | | |
| Early onset (age <48 hrs) | 898 (32.9%) | 168 (35.1%) |
| Late onset community associated, term | 619 (22.7%) | 89 (18.6%) |
| Late onset healthcare associated, pre-term | 799 (29.3%) | 137 (28.7%) |
| Other | 410 (15.0%) | 84 (17.6%) |
| Maximum ventilation support at baseline | | |
| None | 993 (36.4%) | 172 (36.0%) |
| Oxygen supplementation | 517 (19.0%) | 102 (21.3%) |
| CPAP, BiPAP, high flow nasal cannulae | 615 (22.6%) | 104 (21.8%) |
| Invasive ventilation | 601 (22.0%) | 100 (20.9%) |
| Temperature at baseline | | |
| <35.5C | 50 (1.8%) | 8 (1.7%) |
| 35.5-37.9C | 2332 (85.7%) | 404 (84.5%) |
| >=38-<39C | 268 (9.9%) | 52 (10.9%) |
| >=39C | 70 (2.6%) | 14 (2.9%) |
| Difficulty feeding at baseline | 1226 (45.0%) | 239 (50.0%) |
| Lethargy, no/reduced movement at baseline | | |
| Neither | 1772 (65.0%) | 301 (63.0%) |
| Lethargy only | 674 (24.7%) | 129 (27.0%) |
| Reduced/no movement | 280 (10.3%) | 48 (10.0%) |
| Abdominal distension at baseline | 665 (24.4%) | 112 (23.4%) |
| Evidence of shock including cold peripheries at baseline | 578 (21.2%) | 105 (22.0%) |
| Cyanosis at baseline | 329 (12.1%) | 55 (11.5%) |
| Baseline blood culture positive (pathogen) | 484 (17.8%) | 80 (16.7%) |
| Died, overall [1] | 308 (11.3%) | 42 (8.8%) |
| Died on IV antibiotics [2] | 248 (9.1%) | 38 (7.9%) |
