# Supplementary material for: Patterns of antibiotic use, pathogens, and prediction of mortality in hospitalized neonates and young infants with sepsis: A global neonatal sepsis observational cohort study (NeoOBS)
Source: PLoS Med. 2023 Jun 8;20(6):e1004179. doi: 10.1371/journal.pmed.1004179 (PMC10249878; doi:10.1371/journal.pmed.1004179)
Supplement: S13 Table — Notes: This table shows results of unadjusted Cox proportional hazards models for selected factors with site-level random effects: (1) for a baseline NeoSep Severity Score to predict 28-day mortality from factors known at sepsis presentation; and (2) for a NeoSep Recovery Score to predict the daily risk of death while treated with IV antibiotics from daily updated assessments of clinical status.‡ Birth weight and temperature were analyzed as continuous variables using fractional polynomials with powers −2 for birth weight, and powers 2 2 for temperature. For illustrative reasons, in this table we report HRs for specific values. Shaded factors: deliberately not included in model (unmodifiable factors excluded from recovery score: birth weight, time in hospital, gestational age, congenital anomalies). CPAP = Continuous Positive Airway Pressure, BiPAP = Bilevel Positive Airway Pressure, HFNC = High Flow Nasal Cannula, HR = hazard ratio, CI = confidence interval, coef = coefficient. (PDF) [file pmed.1004179.s044.pdf]

S13 Table. Unadjusted analyses.

Factor		NeoSep Severity Score (at presentation) HR (95% CI)	p-value	Coefficient	NeoSep Recovery Score (daily on IV antibiotics) HR (95% CI)	p-value	Coefficient
**Birth weight (kg) ‡**	1.0	3.37 (2.75,4.13)	<0.001	1.21			
	2.0	1.35 (1.29,1.43)		0.30			
	3.0	ref		0			
**Time in hospital**	per additional 24 hours	1.00 (0.98,1.02)	0.96	0.00			
**Gestational age**	per additional week	0.86 (0.84,0.89)	<0.001	-0.15			
**Congenital anomalies**		1.73 (1.22,2.44)	0.002	0.55			
**Maximum respiratory support:**	None	ref	<0.001	0	ref	<0.001	0
	Oxygen supplementation	3.51 (2.16,5.70)		1.26	11.9 (5.50,25.7)		2.47
	CPAP, BiPAP, HFNC	7.48 (4.58,12.2)		2.01	19.7 (9.00,43.2)		2.98
	Invasive ventilation	17.3 (10.8,27.8)		2.85	94.5 (44.4,201.4)		4.55
**Temperature (°C) ‡**	35.5	2.10 (1.47,2.99)		0.74	1.62 (1.23,2.13)	<0.001	0.48
	37	ref		0	ref		0
	38	0.91 (0.77,1.08)		-0.09	1.47 (1.08,2.00)		0.39
	39	1.15 (0.70,1.87)		0.14	3.81 (1.83,7.96)		1.34
**Abdominal distension**		1.73 (1.36,2.20)	<0.001	0.55	2.74 (2.08,3.61)	<0.001	1.01
**Lethargy, no or reduced movement:**	None	ref	<0.001	0	ref	<0.001	0
	Lethargy only	1.49 (1.14,1.95)		0.40	4.27 (3.08,5.91)		1.45
	No/reduced movement (± lethargy)	3.44 (2.42,4.88)		1.23	15.2 (10.6,21.8)		2.72
**Difficulty feeding**		1.68 (1.32,2.14)	<0.001	0.52	3.95 (2.96,5.27)	<0.001	1.37
**Evidence of shock**		3.11 (2.40,4.04)	<0.001	1.14	8.54 (6.28,11.6)	<0.001	2.15
**Cyanosis**		2.08 (1.50,2.87)	<0.001	0.73	5.91 (4.04,8.65)	<0.001	1.78
