# Supplementary material for: Patterns of antibiotic use, pathogens, and prediction of mortality in hospitalized neonates and young infants with sepsis: A global neonatal sepsis observational cohort study (NeoOBS)
Source: PLoS Med. 2023 Jun 8;20(6):e1004179. doi: 10.1371/journal.pmed.1004179 (PMC10249878; doi:10.1371/journal.pmed.1004179)
Supplement: S14 Table — CRIB = Clinical Risk Index for Babies Score; NMR score = Neonatal Mortality Risk score; SAWS = Simplified Age-Weight-Sex score; HR = Heart Rate; LBW = Low birth weight; SpO2 = Oxygen Saturation. (PDF) [file pmed.1004179.s045.pdf]

S14 Table. Comparison of factors in NeoOBS Sepsis Severity score with existing scores and sepsis criteria.

Mortality prediction, sepsis criteria & sepsis severity scores in neonates										
		**General mortality prediction scores**				**Existing Sepsis Criteria**			**Sepsis severity scoring**	
		**High income example**	**LMIC based**							
		**CRIB II mortality prediction score** *(& CRIB)*	**NMR 2000**[3]	**SAWS score**[4]	**Modified sick neonate score**[5]	**pSBI criteria**[6] *(*****=critical illness sign)*	**EMA**[7]	**NeoOBS inclusion criteria**	**nSOFA sepsis severity score**[1,2]	**NeoSep Sepsis severity & recovery scores**
**Purpose of score/criteria**		Mortality prediction in LBW neonates	Mortality prediction in LBW neonates	Mortality prediction in LBW neonates	Mortality prediction in neonates	Clinical diagnosis of sepsis	Enrolment in clinical trials for sepsis	Enrolment in observational study of sepsis	Severity of organ failure and mortality prediction in sepsis	
**Number of neonates from LMIC context included to generate score**		-	550 *(110,176 from high income)*	428	585	-	-	-	None *(679 from high income)*	3204
**Relevant age for use of score**		<12 hrs	<24hrs	<72hrs	Majority <2 days	<60 days	<28 days	<60 days	4-120 days (95% <60d)	<60 days
**Weight category of included babies**		LBW	<2kg	<1.5kg	Majority <2kg	>1.5kg	Any	Any	Majority <1.5kg (88%)	Any
**Specifically developed for sepsis?**		No	No	No	No	Yes	Yes	Yes	Yes	Yes
**Adapted to LMIC context**		No	Yes	Yes	Yes	Yes	No	Yes	No	Yes
**Predominantly community or hospital based**		Hosp	Hosp	Hosp	Hosp	Comm	Hosp	Hosp	Hosp	Hosp
**Factors in score / criteria**	**Baby characteristics**	Birth weight, Sex, *Congenital malformation*	Gestation, Weight, Sex	Sex, Age, Weight, Gestation	Birth weight, Gestation	-	-	-	-	Birth weight, Time in Hospital, Gestational age, Congenital anomalies
	**Vital signs**	Temp	SpO2 at admission	-	HR, SpO2, Glucose, CRT,	Temp <35.5C ≥38C	Temp <35.5C ≥38C, Temp instability,	Temp <35.5C ≥38C,	-	Abnormal temp (<35.5, ≥38 – <39 °C, ≥39°C)

Mortality prediction, sepsis criteria & sepsis severity scores in neonates									
	**General mortality prediction scores**				**Existing Sepsis Criteria**			**Sepsis severity scoring**	
	**High income example**	**LMIC based**							
	**CRIB II mortality prediction score** *(& CRIB)*	**NMR 2000**[3]	**SAWS score**[4]	**Modified sick neonate score**[5]	**pSBI criteria**[6] *(*=critical illness sign)*	**EMA**[7]	**NeoOBS inclusion criteria**	**nSOFA sepsis severity score**[1,2]	**NeoSep Sepsis severity & recovery scores**
				Temp,		HR<10th centile<br>HR >2SD above normal	Temp instability,<br>HR >180<br>HR <100		
**Respiratory signs**	Min/Max FiO2 <12hrs	Max respiratory support	-	Increased respiratory effort	Fast breathing,<br>Severe indrawing	Apnoea,<br>Tachypnoea,<br>Increased O2 requirement,<br>Ventilatory support	Apnoea,<br>Grunting,<br>Cyanosis,<br>Severe chest indrawing,<br>Increased O2 requirement,<br>Ventilatory support	SpO2/FiO2 ratio,<br>Requirement for Invasive ventilation	O2 or ventilation support,<br>Cyanosis (recovery score only)
**Circulatory signs**	-	-	-	-	-	Bradycardia,<br>Tachycardia,<br>Rhythm instability,<br>Reduced urine output,<br>Hypotension,<br>Mottled skin,<br>Impaired perfusion	CRT >3<br>or<br>Mottled skin<br>or<br>Other evidence of shock	Requirement for inotropes,<br>Requirement for steroids for hypotension	Evidence of shock (including any of: CRT >3secs, mottled skin, cold peripheries or 'other')
**Gastrointestinal signs**	-	-	-	-	Poor feeding<br>*Not feeding at all**	Feeding intolerance,<br>Poor sucking,<br>Abdominal distension	Difficulty feeding<br>Or<br>Feeding intolerance,<br>Abdominal distension	-	Difficulty feeding/ feeding intolerance,<br>Abdominal distension

Mortality prediction, sepsis criteria & sepsis severity scores in neonates										
		**General mortality prediction scores**				**Existing Sepsis Criteria**			**Sepsis severity scoring**	
		**High income example**	**LMIC based**							
		**CRIB II mortality prediction score** *(& CRIB)*	**NMR 2000**[3]	**SAWS score**[4]	**Modified sick neonate score**[5]	**pSBI criteria**[6] *(*****=critical illness sign)*	**EMA**[7]	**NeoOBS inclusion criteria**	**nSOFA sepsis severity score**[1,2]	**NeoSep Sepsis severity & recovery scores**
	**Neurological signs**	-	-	-	-	Movement only when stimulated Or *no movement at all*, Convulsions**	Irritability, Lethargy, Hypotonia	No movement or Movement only when stimulated, Bulging fontanelle, Lethargy or drowsiness, Hypotonia / floppiness, Abnormal posturing, Convulsions, Irritability	-	Lethargy, Reduced or no movement
	**Skin / umbilicus**	-	-	-	-	-	Petechial rash, Sclerema	Petechial rash, Multiple or severe skin pustules, Pus from umbilical stump	-	
	**Laboratory signs**	Max base deficit <12h	-	-	-	-	WBC <4 x10^9 >20 x10^9 cells/mL<br><br>IT ratio >0.2<br><br>Platelets <100x10^9 cells/mL	WBC <4 x10^9 >20 x10^9 cells/mL<br><br>Neutrophils <1.5x10^9 cells/mL<br><br>IT ratio >0.2	Severity of thrombocyto-paenia	-

Mortality prediction, sepsis criteria & sepsis severity scores in neonates										
		**General mortality prediction scores**				**Existing Sepsis Criteria**			**Sepsis severity scoring**	
		**High income example**	**LMIC based**							
		**CRIB II mortality prediction score**<br>*(& CRIB)*	**NMR 2000**[3]	**SAWS score**[4]	**Modified sick neonate score**[5]	**pSBI criteria**[6]<br>*(*******=critical illness sign)*	**EMA**[7]	**NeoOBS inclusion criteria**	**nSOFA sepsis severity score**[1,2]	**NeoSep Sepsis severity & recovery scores**
							CRP >15mg/L<br>Procalcitonin ≥2 ng/ml<br><br>Glucose intolerance<br><br>Metabolic acidosis (BE <-10 mEq/L, Lactate >2mMol/L)	CRP >10mg/L<br><br>Metabolic acidosis (BE <-10 mEq/L, Lactate >2mMol/L)		

References

1. Wynn, J. L. & Polin, R. A. A neonatal sequential organ failure assessment score predicts mortality to late-onset sepsis in preterm very low birth weight infants. *Pediatr. Res.* **88**, 85–90 (2020).
2. Wynn, J. L. *et al.* Timing of Multiorgan Dysfunction among Hospitalized Infants with Fatal Fulminant Sepsis. *Am. J. Perinatol.* **34**, 633–639 (2017).
3. Medvedev, M. M. *et al.* Development and validation of a simplified score to predict neonatal mortality risk among neonates weighing 2000 g or less (NMR-2000): an analysis using data from the UK and The Gambia. *Lancet Child Adolesc. Heal.* **4**, 299–311 (2020).
4. Rosenberg, R. E. *et al.* Simplified Age-Weight Mortality Risk Classification for Very Low Birth Weight Infants in Low-Resource Settings. *J. Pediatr.* **153**, 519-524.e3 (2008).
5. Mansoor, K. P. *et al.* Modified Sick Neonatal Score (MSNS): A Novel Neonatal Disease Severity Scoring System for Resource-Limited Settings. *Crit. Care Res. Pract.* **2019**, 9059073 (2019).
6. WHO. *Managing possible serious bacterial infection in young infants when referral is not feasible*. (2015).
7. Tuzun, F. *et al.* Is European Medicines Agency (EMA) sepsis criteria accurate for neonatal sepsis diagnosis or do we need new criteria? *PLoS One* **14**, e0218002–e0218002 (2019).
